# Patient‐derived acellular ascites fluid affects drug responses in ovarian cancer cell lines through the activation of key signalling pathways

**DOI:** 10.1002/1878-0261.13726

**Published:** 2024-09-08

**Authors:** Katharina Bischof, Andrea Cremaschi, Lena Eroukhmanoff, Johannes Landskron, Lise‐Lotte Flage‐Larsen, Alexandra Gade, Line Bjørge, Alfonso Urbanucci, Kjetil Taskén

**Affiliations:** ^1^ Department of Cancer Immunology, Institute for Cancer Research University of Oslo Norway; ^2^ Division of Cancer Medicine, Department of Gynecological Oncology Oslo University Hospital Norway; ^3^ Centre for Molecular Medicine Norway (NCMM) Nordic EMBL Partnership, University of Oslo Norway; ^4^ Oslo Centre for Biostatistics and Epidemiology University of Oslo Norway; ^5^ Singapore Institute for Clinical Sciences, A*STAR Singapore; ^6^ Yong Loo Lin School of Medicine National University of Singapore Singapore; ^7^ Department of Obstetrics and Gynaecology Haukeland University Hospital Bergen Norway; ^8^ Department of Clinical Science, Centre for Cancer Biomarkers CCBIO University of Bergen Norway; ^9^ Faculty of Medicine and Health Technology TAYS Cancer Centre and FICAN Mid, Tampere University Finland; ^10^ Department of Tumor Biology, Institute for Cancer Research University of Oslo Norway; ^11^ Institute of Clinical Medicine University of Oslo Norway

**Keywords:** ascites, chemoresistance, drug sensitivity, intracellular signalling, ovarian cancer, phospho flow

## Abstract

Malignant ascites is commonly produced in advanced epithelial ovarian cancer (EOC) and serves as unique microenvironment for tumour cells. Acellular ascites fluid (AAF) is rich in signalling molecules and has been proposed to play a role in the induction of chemoresistance. Through *in vitro* testing of drug sensitivity and by assessing intracellular phosphorylation status in response to mono‐ and combination treatment of five EOC cell lines after incubation with AAFs derived from 20 different patients, we investigated the chemoresistance‐inducing potential of ascites. We show that the addition of AAFs to the culture media of EOC cell lines has the potential to induce resistance to standard‐of‐care drugs (SCDs). We also show that AAFs induce time‐ and concentration‐dependent activation of downstream signalling to signal transducer and activator of transcription 3 (STAT3), and concomitantly altered phosphorylation of mitogen‐activated protein kinase kinase (MEK), phosphoinositide 3‐kinase (PI3K)–protein kinase B (AKT) and nuclear factor NF‐kappa‐B (NFκB). Antibodies targeting the interleukin‐6 receptor (IL6R) effectively blocked phosphorylation of STAT3 and STAT1. Treatments with SCDs were effective in reducing cell viability in only a third of 30 clinically relevant conditions examined, defined as combinations of drugs, different cell lines and AAFs. Combinations of SCDs and novel therapeutics such as trametinib, fludarabine or rapamycin were superior in another third. Notably, we could nominate effective treatment combinations in almost all conditions except in 4 out of 30 conditions, in which trametinib or fludarabine showed higher efficacy alone. Taken together, our study underscores the importance of the molecular characterisation of individual patients' AAFs and the impact on treatment resistance as providing clinically meaningful information for future precision treatment approaches in EOC.

## Graphical abstract

We systematically and comprehensively investigated the effects of adding patient‐derived acellular ascites fluid (AAF) to ovarian cancer cell lines. By performing high‐throughput drug sensitivity screens together with phospho flow cytometry, we show that the incubation with AAFs affects chemosensitivity by activating key signalling pathways. We could nominate novel therapeutics or combinations as potential treatment alternatives for most ovarian cancers.

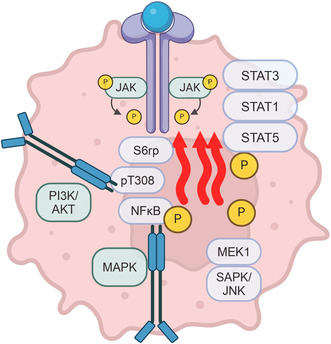

**Abbrevations**
AAF: acellular ascites fluid
AUC: area under the curve
CCL18: chemokine‐ligand 18
CR: combination ratio
DSS: drug sensitivity score
EOC: epithelial ovarian cancer
IL‐10: interleukin‐10
IL‐6: interleukin 6
IL6R: interleukin‐6 receptor
IL‐8: interleukin‐8
MEK: mitogen‐activated protein kinase kinase
NFκB: nuclear factor NF‐kappa‐B
RFU: relative fluorescence unit
SCD: standard‐of‐care drug
STAT3: signal transducer and activator of transcription 3
Tregs: regulatory T cells
VUS: volume under the surface
ZIP: zero‐interaction potency

## 1 Introduction

Epithelial ovarian cancer (EOC) remains the most fatal gynaecological malignancy with a 5‐year survival rate of 30% for advanced stages [1]. Standard front‐line therapy of EOC typically consists of radical debulking surgery and cytotoxic treatment with standard‐of‐care drugs (SCD) carboplatin and paclitaxel administered in the neoadjuvant or adjuvant setting. Maintenance regimens after front‐line treatment include VEGF—or PARP—inhibitors [2, 3, 4, 5]. The term ‘epithelial ovarian cancer’ has been shown to comprise a heterogeneous group of tumours, that can be categorised into distinct phenotypic and molecular subtypes. The high‐grade serous carcinoma subtype accounts for around 70% of EOC and thereby represents the most common subtype besides low‐grade serous carcinoma, mucinous carcinoma, clear‐cell carcinoma and endometrioid carcinoma [2, 5, 6]. Ovarian tumours containing both sarcomatous and epithelial components, so called carcinosarcomas, are known to constitute a rare and aggressive entity [7, 8]. The last decade has brought substantial progress in the molecular characterisation of EOC [9, 10, 11, 12]. However, most studies focused on drivers of innate and acquired chemoresistance in EOC cells [9, 13] with, to date, limited success in clinical translation. Interestingly, the build‐up of malignant abdominal fluid, termed malignant ascites, is seen throughout EOC subtypes in advanced stages at the time of diagnosis and in the recurrent setting and has been assigned a pivotal role in developing recurrent and chemoresistant disease [14]. As such, the role of ascites as a unique microenvironment for EOC cells has been intensively studied. Ascites is known to contain soluble signalling mediators acting as pro‐tumourigenic and anti‐tumourigenic factors, cytokines, growth factors, and bioactive lipids, as well as cellular components like fibroblasts, mesothelial cells, adipocytes, adipose tissue‐derived stromal cells, bone marrow‐derived stem cells and immune cells [15, 16, 17, 18, 19, 20]. Ascites‐derived stromal cells and inflammation‐mediating cytokines present in acellular ascites fluid (AAF) such as interleukin‐6 (IL‐6), interleukin‐8 (IL‐8), interleukin‐10 (IL‐10) and chemokine‐ligand 18 (CCL18) have previously been shown to promote migration, angiogenesis, tumour growth and the development of chemoresistance [18, 19, 20, 21, 22, 23, 24, 25] but this has not been investigated systematically. The presence of high levels of regulatory T cells (Tregs) in ascites has been linked to the inhibition of anti‐tumour immunity and impaired prognosis [16, 17], while the presence of tumour infiltrating lymphocytes inside the cancer tissue is established to be prognostically favourable [26, 27]. Ascites‐derived EOC cells are often present as cell clusters, so‐called spheroids, and have been shown to present inflammation‐associated as well as anti‐apoptotic gene expression programmes, indicating that soluble and cellular ascites components directly impact tumour cells [11, 28, 29, 30]. The study of downstream pathways in EOC cells after the addition of AAF has further assigned soluble factors a central role in key regulatory cell signalling hubs like JAK/STAT3, ERK1/2‐Elk‐1, PyK2, PI3K/Akt, WNT, as well as TRAIL‐induced apoptosis [31, 32, 33, 34]. Therefore, understanding the effect of AAFs on key downstream pathways can reveal their chemoresistance‐inducing potential on EOC cells.

In the present study, we acquired over 10 000 data points on the viability of well‐characterised EOC cell lines [35] by high‐throughput drug sensitivity screening to systematically and comprehensively investigate the effects of AAF, derived from a cohort of 20 patients with treatment‐naïve EOC, on drug sensitivity and signalling. We show that the incubation with AAF affects chemosensitivity in EOC cell lines by activating key signalling pathways. We further test and confirm novel therapeutics as valid treatment alternatives to SCD and in synergistic combinations.

## 2 Materials and methods

### 2.1 Patient materials

The study was approved by the Regional Ethics Committee for Medical and Health for South‐Eastern Norway (permit no. 2018/1489, REK SØ‐C), and written informed consent was obtained from all patients before enrolment. The study methodologies conformed to the standards set by the Declaration of Helsinki. Ascites were prospectively collected from 20 women undergoing surgical treatment for EOC at Haukeland University Hospital, Bergen, Norway in the time between October 2008 and September 2010. Routine histopathological analysis of ovarian tumours was performed at Haukeland University Hospital, Department of Pathology, Bergen, Norway, by experienced pathologists. Clinicopathological data from all patients were collected (Table S1) including age at primary diagnosis, FIGO tumour stages [36], preoperative CA125 levels, histology subtypes and level of macroscopic complete cytoreduction.

### 2.2 Preparation of acellular ascites fluid

Acellular ascites fluid at a volume between 0.1 and 0.5 L was processed by filtration through a 40 μm strainer (Corning Inc., Corning, NY, USA). Resulting suspensions were centrifuged at 350 ***g*** for 10 min in 50 mL tubes to remove cellular components. Resulting AAF was stored at −80 °C until use.

### 2.3 Ovarian cancer cell line culture and trypsinisation protocol

The human ovarian cancer cell lines OVCAR3 (RRID: CVCL_0465), OVCAR5 (RRID: CVCL_1628), OVCAR8 (RRID: CVCL_1629) and NCI/ADR‐RES (hereafter designated as NCI) (RRID: CVCL_1452) were obtained from the National Institute of Health as a part of the National Cancer Institute (NCI)‐60 cancer cell line panel, the SKOV3 cell line (RRID: CVCL_0532) was provided through collaboration with the University of Bergen. Cells were authenticated by Eurofins Genomics (Ebersberg, Germany), cultured according to the supplier's instructions in RPMI‐1640 media, supplemented with 2 mm glutamine, 100 U·mL^−1^ penicillin G, 100 μg·mL^−1^ streptomycin and 10% heat‐inactivated foetal calf serum (FCS). All cells were cultured at 37 °C in a humidified atmosphere containing 5% CO_2_. All experiments were performed with mycoplasma‐free cells. For the experiments with phospho flow, cells were washed twice with ice‐cold PBS and then incubated with ice‐cold trypsin (Gibco®Trypsin 2.5%; Thermofisher, Waltham, MA, USA) until detachment as previously described [37]. FCS‐containing culture media was added to quench the trypsin reaction.

### 2.4 Drug screening

All drug screens were performed using 384‐well plates (Greiner Bio‐One #781098; Merck, Darmstadt, Germany). All compounds were stored at a concentration of 10 mm in DMSO (except carboplatin which was stored in water) either at −80 °C or under a humidity‐controlled nitrogen atmosphere at room temperature. Cells were plated (500 cells/well) in 10 μL culture media (alone or in the presence of AAFs) and cultured overnight to allow attachment. Then respective volumes of compounds were transferred using an Echo 550 acoustic dispenser (Labcyte, San Jose, CA, USA) either from the 10 mm stock solution or from DMSO (carboplatin : water) diluted samples to reach the final assay concentration. After addition of 15 μL culture media, cells were incubated for 48 h followed by a cell viability assay using the CellTiter‐Glo luminescence ATP assay (Promega, Madison, WI, USA). Bioluminescence was measured on a Synergy Neo 2 plate reader (BioTek, Santa Clara, CA, USA).

### 2.5 Drug library, combinatorial drug screen

A drug library of 11 SCD or FDA‐approved drugs, novel for the treatment of EOC, was tested for single drug efficacy at different concentrations over a clinically relevant range from 1 μm to 10 mm. Drug combinations were selected according to acquired data on intracellular phosphorylation alterations. Combinations were tested with one compound at its fixed average IC_20_ concentration (priming drug) based on drug sensitivity data (listed in Table S2) after optimal curve fitting and outlier removal. To test the effect of drug combinations, the priming drug was combined with a less potent drug tested at six ascending concentrations.

### 2.6 Concentration‐response, drug sensitivity screening and cell viability assays

Concentration‐responses of cell lines, incubated in a 1 : 1 dilution of AAF in media or culture media alone, were assessed by treatment with paclitaxel or carboplatin (for concentrations, see Table S2) for a total of 48 h. Cell viability was assessed by the CellTiterGlo Luminescence ATP assay (Promega) according to the manufacturer's recommendations, and luminescence was measured with an Envision Xcite plate reader (Perkin Elmer, Shelton, CT, USA). Drug sensitivity scores (DSS) of cell lines SKOV3, OVCAR3, OVCAR5, OVCAR8 and NCI were calculated [38] after a quality control assessment, including calculation of the z‐prime factor, performed for each plate used in the screening. Relative percentage (%) cell viability was calculated by normalising to negative and positive control wells. Cell viability and proliferation of SKOV3, OVCAR3, OVCAR5, OVCAR8 and NCI cells were assessed after incubation with five different AAFs for 48 h. Three technical replicates and two biological replicates per data point were acquired.

### 2.7 Analysis of concentration‐response data

To normalise the concentration‐response data in presence of AAF or media, we used vehicle treated cells (DMSO for all the drugs except carboplatin that was dissolved in water). The percentage of vehicle varied according to the final concentration of drugs. Therefore, the fluorescence levels of each well were normalised against the percentage of vehicle used to dissolve the drugs (DMSO—or water for carboplatin) to control. The level of cell viability in absence of drugs (in both DMSO and water) here named ‘0 m’ was calculated using the highest and lowest percentage of DMSO. We calculated the means of the Relative Fluorescence Unit (RFU) for the high and low DMSO and used these as numerators in the ratio with the mean of all the RFUs signals for all the DMSO points. As carboplatin was dissolved in water, this ratio was equal to 1 (alias 100% viability). For the combination experiments, we computed the Combination Ratio (CR) between the drug sensitivity score (DSS) obtained from the combined and the monotherapy experiments as described by Kurtz et al. [39]. Each DSS score was first augmented by 100 in order to avoid unfeasible results when one or both the DSS scores are equal to zero. Values of the CR larger than 1 indicate improved efficacy of the combined drugs with respect to the one selected for the comparison. Synergy scores based on the zero‐interaction potency (ZIP) model were computed as earlier reported [40], to assess the synergistic behaviour of the drug combinations. The treatments were ranked based on CR or DSS measures, identifying the best treatment options for a specific condition, defined hereafter as combination of a specific patient‐derived AAF (or media) with one of the five cell lines. As a final measure of efficacy for combination experiments plotted as surface plots, we used volume under the surface (VUS) as a 3D‐measure analogous to area under the curve (AUC) [41]. This measure is computed using the trapezoid method along the two directions spanned by the concentrations of the two drugs combined, and it is normalised by the total volume of the cube containing the response surface. To obtain interpretable values (high = effective, low = non‐effective), we report the values of 100‐VUS. A ranking of the most effective drug combinations for individual conditions was performed based on CR.

### 2.8 Trypsinisation, stimulation and fluorescent cell barcoding

SKOV3 and OVCAR8 cells were cultured for 48 h in 6‐well plates, 1.5 × 10^5^ cells per well. Prior to stimulation, cells were serum starved for 24 h in order to synchronise cells to the same cell cycle phase and to create a low baseline phosphorylation state. Incubation was performed by adding AAF (*n* = 20) at an optimised ratio of 1 : 1 resulting from titrating experiments illustrated in Fig. S1, unless stated otherwise, with FCS‐free culture media for 20 min (see Fig. S2). After stimulation, cells were immediately placed on ice to quench cell signalling, trypsinised [37] and fixed [42] as described before. Cells were subsequently washed, resuspended in ice cold PBS and fixed for 10 min in pre‐warmed BD Phospho flow™ Fix Buffer Ι (BD Biosciences, Franklin Lakes, NJ, USA) at 37 °C, followed by two washes with PBS. Fixed cells were resuspended in PBS and incubated with different concentrations of each of the barcoding fluorochromes (Alexa Fluor 488, Cat#A‐20100, Pacific Orange Cat#P30253 and Pacific Blue Cat#P10163; Life Technologies, Carlsbad, CA, USA) diluted in DMSO, in a 96‐well plate. Final concentrations of the barcoding reagents were 100, 25, 6.25 and 0.69 ng·mL^−1^. After staining for 20 min at room temperature in the dark, cells were washed twice with flow wash (PBS, 10% FCS and 0.09% sodium azide), combined, permeabilized with BD Phospho flow™ Perm Buffer ΙΙΙ (BD Biosciences), and stored at −80 °C.

### 2.9 Phospho flow cytometry analysis

Permeabilized cells were washed three times with flow wash (BD Biosciences), centrifuged for 5 min at 500 ***g***, resuspended and distributed in 96‐well plates as previously described [43]. The cells were stained with phospho‐specific antibodies (listed in Table S3) and incubated in the dark at room temperature for 30 min. The samples were then washed once in PBS, resuspended with self‐made wash buffer, described above, and analysed with a BD FACS Canto (BD Biosciences) cytometer equipped with 405, 488 and 633 nm lasers. Data were analysed in Cytobank (https://www.cytobank.org/).

### 2.10 Blockage of IL‐6 receptor using a monoclonal antibody

A humanised, monoclonal antibody targeting IL‐6 receptor (IL6R) alpha (Cat#MAB227; Bio‐Techne, Minneapolis, MN, USA) was titrated at a concentration between 0.1 and 10 μg·mL^−1^ and cells were stimulated with AAF in the presence of the IL6R antibody for 20 min prior to read‐out.

### 2.11 Statistical analyses

Statistical analyses were performed using r software (https://www.r‐project.org) and prism 7 (GraphPad Software, San Diego, CA, USA). The fitting of the concentration‐response curves was performed by using the *drm* function of the r package *drc*, specifically by fitting a 4‐parameter logistic curve to each set of concentration‐response data. The output of the fitting provided estimates of IC20, IC50 and Slope parameters. To be able to better depict altered chemosensitivity, we calculated the DSS, which is a normalised measure of the area under the curve. When analysing the effect of SCDs, we performed 2‐way ANOVA tests for the computed DSS values using cell lines and the AAF stimulation as factors. Tukey *post‐hoc* tests were used to perform multiple comparisons among data obtained in exposure to AAF versus culture media. Furthermore, we provide *P*‐values for one‐way ANOVA tests when one of the factor values is fixed (e.g. for one specific cell line). When needed, heatmaps of the DSS are produced using the function *heatmap.2* of the r package *gplots*. This function allows the clustering of the DSS via a hierarchical clustering sub‐routine, which is here used with Euclidean distance and Ward‐d2 method specifications. We tested whether the correlations between different phospho‐protein levels were significantly different from zero via Pearson's *r* test (*P*‐values adjusted). Multiple testing was corrected for with the Holm method.

## 3 Results

### 3.1 Incubation of ovarian cancer cell lines with acellular ascites fluid induces chemoresistance

**Fig. 1:**
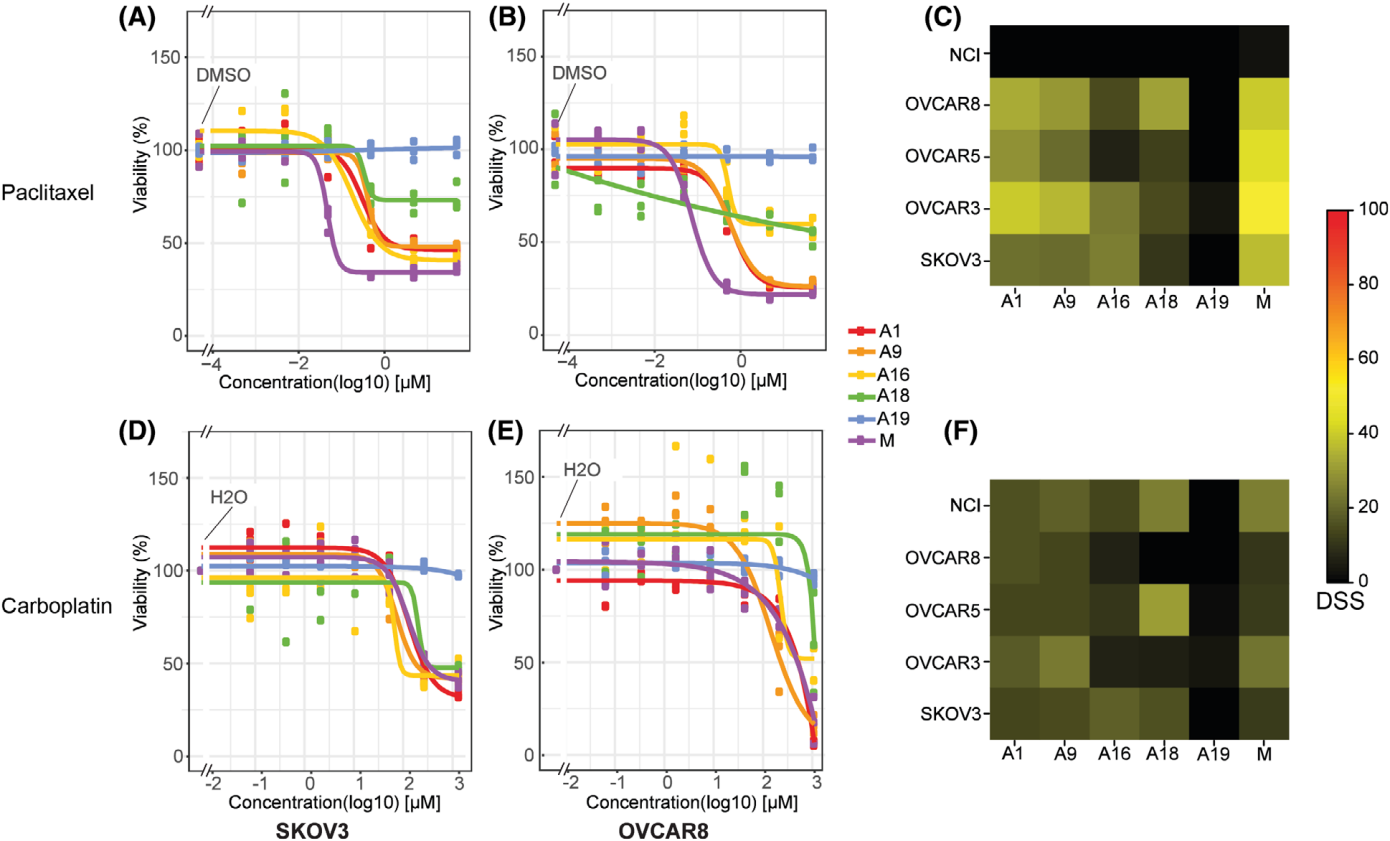
Concentration‐responses and drug sensitivity scores (DSS) to treatment with paclitaxel and carboplatin of cell lines SKOV3 and OVCAR8 after incubation with patient‐derived ascites. Concentration responses to treatment with paclitaxel (A, B) and carboplatin (D, E) in the ovarian cancer cell lines SKOV3 and OVCAR8, incubated in culture media alone (M) or with five different patient‐derived acellular ascites fluids (A) (*n* = 5). Concentration responses have been fitted to a 4‐parameter log‐logistic model for which the lower boundary was set to zero. Heatmap illustrating drug sensitivity scores (DSS) to treatment with Paclitaxel (C) and Carboplatin (F) of the SKOV3, OVCAR3, OVCAR5, OVCAR8 and NCI cell lines incubated in culture media alone or with five different ascites samples (A) (*n* = 5).

To evaluate chemosensitivity after treatment with SCDs paclitaxel and carboplatin, five well‐characterised epithelial ovarian cancer cell lines were cultured in the presence of five different AAFs (A) or culture media alone (Fig. 1 and Fig. S3) which yields 30 different conditions. We subjected each of the 30 conditions to treatment with increasing concentrations of paclitaxel (Fig. 1A,B and Fig. S3A–C) and carboplatin (Fig. 1D,E and Fig. S3D–F). The addition of AAF resulted in a variable increase in chemoresistance to SCDs compared to cells cultured in media. For example, incubation with A19 resulted in a consistent viability of both cell lines, even during treatment with high concentrations of SCDs paclitaxel and carboplatin, indicating complete induction of chemoresistance. Incubation with A18 also yielded a robust viability of the cell lines during treatment with paclitaxel compared to media alone (Fig. 1D,E and Fig. S3).

Variation of sensitivity to SCD for individual cell lines was also examined by calculating individual DSS for treatment with paclitaxel (Fig. 1C) and carboplatin (Fig. 1F) after incubation with AAF or media alone. The effects of A19 in inducing chemoresistance were consistent across cell lines. After incubation with A16 and A18, a decreased DSS was observed for OVCAR8, OVCAR5 and OVCAR3 cell lines. The NCI cell line is known to be multidrug resistant and showed resistance to paclitaxel and in part to carboplatin (Fig. 1C,F). Overall, we find that exposure to AAFs induces chemoresistance in EOC cells.

### 3.2 Acellular ascites fluid activates key intracellular signalling pathways

**Fig. 2:**
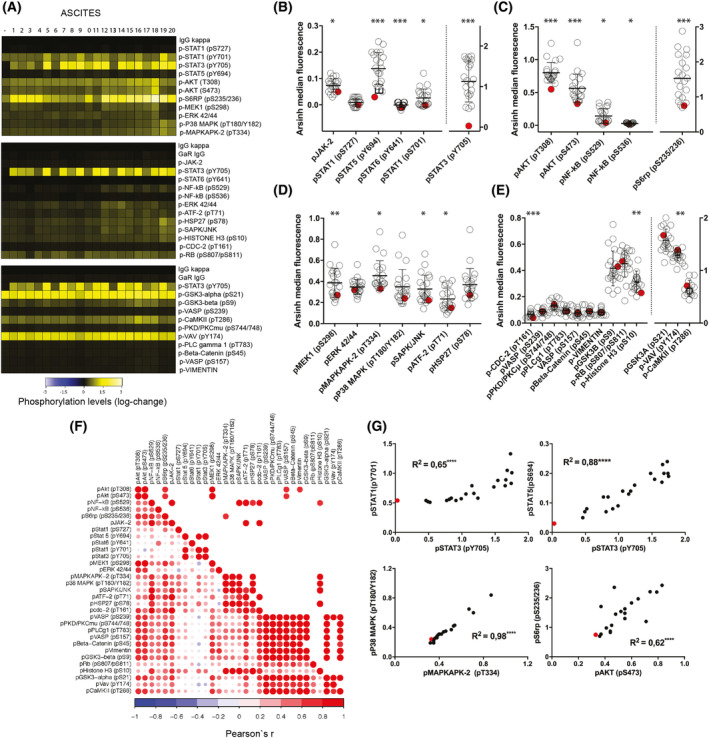
Intracellular pathway activation following incubation of SKOV3 cells with patient‐derived ascites. (A) Heatmap illustrating individual phospho‐signalling profiles for SKOV3 cells after incubation with acellular ascites fluid (*n* = 20, labelled #1–20) or media (−). The target pSTAT3 (pY705) was included in all three panels that were run. IgG served as a control and data are normalised to IgG control in each heatmap (representing different panel runs). (B–E) Variation in phosphorylation levels in downstream effectors in SKOV3 cells (red dots represent signal in cells in media alone used as control). (B) JAK/STAT, (C) PI3K/AKT, (D) MAPK/MEK/ERK pathways and (E) other intracellular markers. *t*‐tests comparing the mean fluorescence values in SKOV3 cells versus control are shown, mean and SD are plotted. The multiple tests are corrected with Holm method. *P*‐value ranges: *P ≤ 0.05, **P ≤ 0.01, ***P ≤ 0.001. (F) Correlation matrix illustrating the degree of correlation among protein phosphorylation levels of intracellular effectors after incubation with AAF (*n* = 20) The upper part only presents the significant values, corrected for multiplicity by the Holm Method (*P* < 0.05) (G) Correlation coefficients for univariate correlation of phosphorylation levels of selected key downstream effectors. Red dots indicate the phosphorylation levels measured in cells cultured in media alone.

To investigate how AAF affects intracellular pathways, we performed phospho flow cytometry after cold trypsinisation of EOC cells exposed to AAF from 20 different patients (see Section 2). To be able to assess the dynamic changes in phosphorylation levels of 31 downstream effectors in the SKOV3 cell line after stimulation (Fig. 2A), cells were cultured in FCS‐free media alone to ensure a synchronisation of cell cycle states and to create low baseline phosphorylation levels or incubated with the different AAFs. Stimulation with AAF resulted in a significant increase in STAT3(pY705) phosphorylation compared to cells cultured in FCS‐free media (*P* < 0.001) in all samples. Within the JAK–STAT pathway, we further observed a significant activation of STAT5 (pY694), STAT6 (pY641) (*P* < 0.001), JAK2 and STAT1 (pS701) (*P* < 0.05) in SKOV3 cells (Fig. 2B). Within the PI3K/AKT pathway, an activation of S6RP (pS235/236) was shown after stimulation with AAF (*P* < 0.001), as well as phosphorylation of AKT on effectors pT308 and pS473 (*P* < 0.001), and activation of NFkB (both pS529 and pS536) (*P* < 0.05) in SKOV3 cells (Fig. 2C). Further, downstream effectors of the MAPK/MEK/ERK pathway were shown to be differentially phosphorylated in MAPKAPK‐2 (pT334), SAPK/JNK, ATF‐2 (pT71) (*P* < 0.05) and MEK1 (pS298) (*P* < 0.01) in the same cells (Fig. 2D). Other intracellular proteins that displayed higher levels of phosphorylation compared to cells growing in FCS‐free medium alone were CDC‐2 (pT161) (*P* < 0.001), Histone H3 (pS10) and VAV (pY174) (*P* < 0.01) (Fig. 2E). By performing univariate correlation analysis of SKOV3 cells stimulated with AAF, we found phosphorylation status of downstream effectors within pathways to be highly correlated with one another (Fig. 2F). Specifically, phosphorylation level of STAT3 (pY705) correlated with phosphorylation of STAT1 (pY701) (*R* = 0.65, *P* < 0.0001) and STAT5 (pY694) (*R* = 0.88, *P* < 0.0001). Phosphorylation level of MAPKAPK‐2 (pT334) was linked with activation of 38MAPK (pT180/Y182) (*R* = 0.98, *P* < 0.0001), and phosphorylation of AKT (pS473) was significantly associated with activation of S6RP (pS235/236) (*R* = 0.62, *P* < 0.0001) in SKOV3 cells (Fig. 2G). Corresponding data for incubation of OVCAR8 cells are available in Fig. S4A–G.

We next performed phospho flow on EOC cell lines SKOV3, OVCAR5, OVCAR8 and NCI after incubation with ascending concentrations of one selected ascites. A15 was titrated in ratios of 25%, 50%, 75% and 100% to FCS‐free culture media, and we observed a marked increase in signalling levels in the target S6RP, consistent in all cell lines analysed (Fig. S1). We further detected a concentration‐dependent increase in STAT3 signalling in SKOV3 cells, but this was not as pronounced in cell lines OVCAR8, OVCAR5 and NCI. The phosphorylation status of Akt showed a moderate increase after stimulation with ascending doses, with a peak at around 40% A15 in media, most prominently in SKOV3 cells. For intracellular effectors RB, NFκB, MAPKAPK‐2, SAPK/JNK and P38, no clear impact on signalling could be observed. Overall, we show that JAK/STAT, PI3K/AKT and MAPK/MEK/ERK pathways are activated by AAF in EOC cells.

### 3.3 IL‐6 receptor blockage counteracts increased intracellular phosphorylation of STAT3 in presence of acellular ascites fluid

**Fig. 3:**
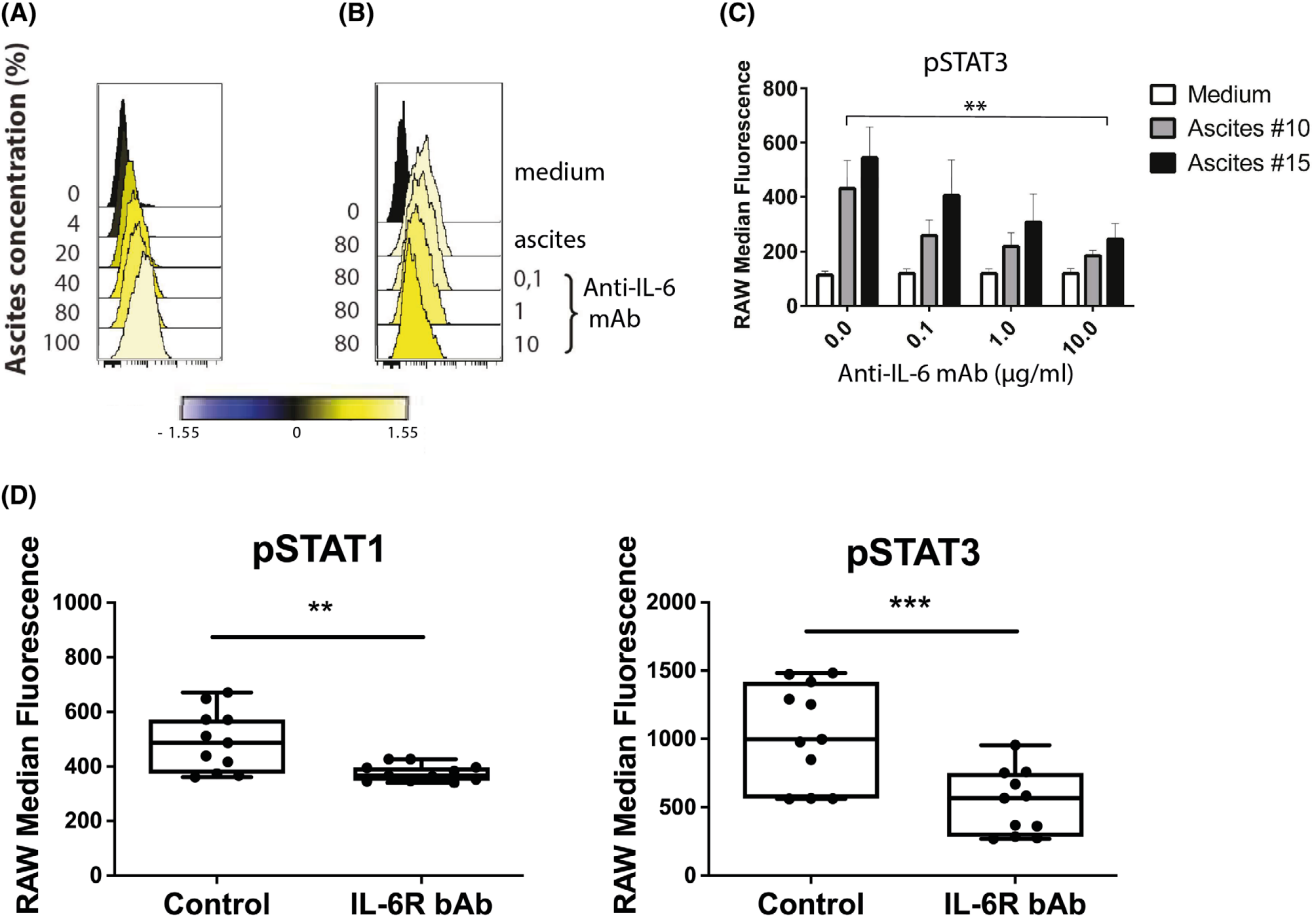
Effects of IL‐6 receptor (IL‐6R) blockage on protein phosphorylation status. Intracellular STAT3 phosphorylation status was assessed by phospho flow cytometry in SKOV3 cells treated with (A) ascending concentrations of patient‐derived ascites for 20 min or (B) acellular ascites fluid together with IL‐6R alpha blocking antibody at the indicated concentrations. (C) Effects of treatment with IL‐6 blocking mAb on ascites‐induced STAT3 phosphorylation. Data are mean (*n* = 3) of raw median fluorescence, and error bars represent the standard error of the mean, paired *t*‐test was performed to compare fluorescence. (D) Effects of the addition of IL‐6R blocking mAb (10 μg·mL^−1^) on protein phosphorylation status compared to IgG kappa control in the presence of acellular ascites fluid (*n* = 10 patients). Plots display median (raw median fluorescence), individual datapoints illustrated as 25th–75th percentiles as boxplots with minimum and maximum values as error bars (***P < 0.001, **P < 0.01 paired *t*‐test).

The use of monoclonal antibodies targeting IL6R has been introduced as a therapeutic strategy in EOC [18, 22, 44, 45]. Therefore, we sought to examine whether the addition of blocking antibodies targeting IL6R significantly alters STAT3 signalling. After 20 min incubation together with ascending doses of one AAF (A15), we found the median intracellular STAT3 protein phosphorylation status in SKOV3 cells to be impacted by the concentration of added A15 (Fig. 3A). Adding 80% AAF and treatment with the mAb targeting IL6R at a concentration range of 0.1–10 μg·mL^−1^ resulted in a clear concentration‐dependent reduction of intracellular STAT3 phosphorylation in SKOV3 cells (Fig. 3B). Treatment with a mAb targeting IL‐6R at a concentration of 10 μg·mL^−1^ showed a significant reduction of phosphorylation levels of STAT3 in SKOV3 cells (*P* < 0.01) after incubation with AAF from two different patients (A10 and A15) (Fig. 3C). Next, we quantified the phosphorylation of key signalling proteins in SKOV3 cells treated with mAbs targeting IL‐6 receptor at a concentration of 10 μg·mL^−1^ in the presence or absence of patient‐derived AAF (*n* = 10). STAT1 (*P* < 0.01) and STAT3 (*P* < 0.001) signalling was significantly reduced in samples treated with IL6R blocking mAb (Fig. 3D). To study how AAF affected intracellular phosphorylation over time, we assessed the phosphorylation status of important intracellular hubs in EOC cell lines after incubation with A10 at different time points. The 31 targets included in the analysis are listed in Fig. 2A. We found phosphorylation of STAT3 to be significantly upregulated over time in SKOV3 cells (*P* < 0.001), OVCAR8 cells (*P* < 0.01) and OVCAR5 cells (*P* < 0.05), while S6RP was significantly activated following incubation in SKOV3 and OVCAR5 cells (*P* < 0.001) as well as in OVCAR8 and NCI cells (*P* < 0.01) (Fig. S2A). For downstream effectors AKT, GSK3B, GSK3A, STAT1, MEK1 and RB, we did not observe temporal activation (Fig. S2B). By performing one‐way ANOVA analysis, we identified the total number of analysed intracellular proteins that were significantly impacted after incubation or remained unregulated or showed no phosphorylation at all. Overall, we found around 20% of effectors to be regulated following incubation, while phosphorylation status remained unchanged in around half of the proteins. In around 30% of effectors, no phosphorylation was detected. This observation was consistent across SKOV3, OVCAR8 and OVCAR5 cells, while NCI cells showed increased phosphorylation in a smaller fraction of downstream factors (Fig. S2C). Taken together, these data show that incubation with a mAb targeting IL6R at a concentration of 10 μg·mL^−1^ can reverse STAT3 activation mediated by AAF.

### 3.4 Cell lines cultured in the presence of ascites fluid can exert sensitivity to novel drugs compared to standard‐of‐care drugs

**Fig. 4:**
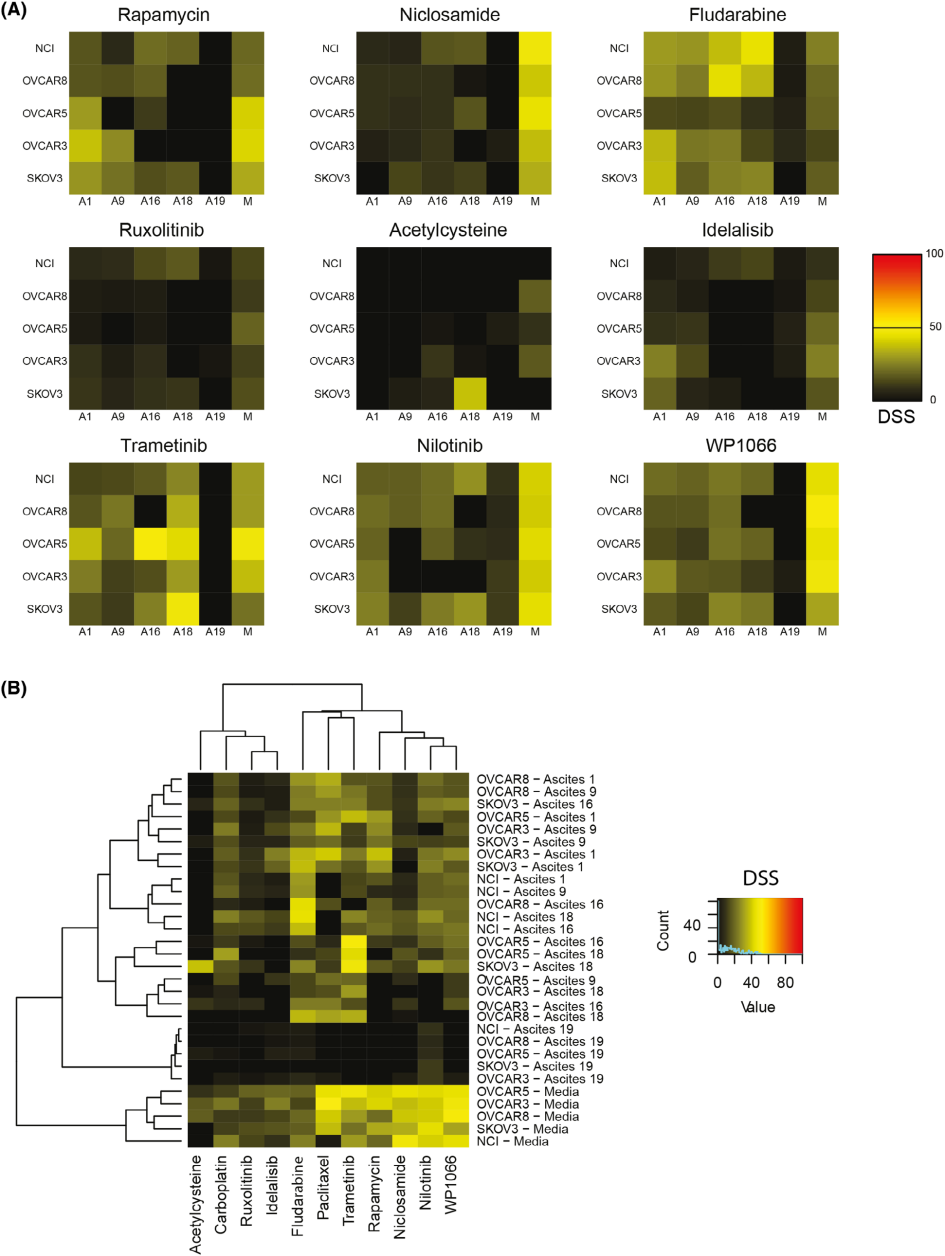
Monotherapy with novel therapeutics. (A) Heatmaps illustrating response to treatment with nine novel therapeutics. Drug sensitivity score (DSS) for five individual ovarian cancer cell lines incubated with acellular ascites fluid (*n* = 5) or media alone. (B) Displays heatmap of hierarchical clustering of DSS for individual cell lines, culture conditions and treatment based on Euclidean distance and Ward‐d2 method (for combinations of standard‐of‐care drugs and novel therapeutics). DSS is calculated as stated in Section 2.

To assess whether the chemoresistance‐inducing potential of AAF on EOC cell lines remains limited to treatment with carboplatin and paclitaxel, we screened a total of nine compounds with FDA‐approval for other indications than standard‐of‐care in EOC, rapamycin, niclosamide, fludarabine, ruxolitinib, acetylcysteine, idelalisib, trametinib, nilotinib and WP1066 (Fig. 4 and Fig. S5). Drugs were selected as they were expected to perturb intracellular signalling pathways shown to be regulated in the phosphoflow analyses (ruxolitinib, WP1066, rapamycin, trametinib, idelalisib), because they had yielded effects in earlier drug repurposing experiments examining ovarian cancer cells (niclosamide, acetylcysteine) or as candidates from other earlier drug panels used in our group (fludarabine, nilotinib). To better evaluate sensitivity of combinations of cell lines and AAF to single drugs we calculated drug sensitivity (see Section 2) in each condition (Fig. 4A). Incubation with AAF resulted in reduced chemosensitivity to treatment with rapamycin, niclosamide, nilotinib and WP1066 in individual cell lines. Stimulation with AAF did not impact chemosensitivity for treatment with ruxolitinib, acetylcysteine, idelalisib and trametitinib. However, the presence of AAF enhanced chemosensitivity to treatment with fludarabine (Fig. 4A). We found that adding A19 consistently induced chemoresistance to all drugs analysed. Carboplatin, acetylcystein, niclosamide, idelalisib, nilotinib, rapamycin and WP1066 did not have a cell line‐specific effect (Table S4). Hierarchical clustering of DSS from each condition identified classes of conditions displaying sensitivity to treatments with SCD and novel therapeutics. Fludarabine showed an enhanced effect after incubation with A16 and A18 on cell lines, including NCI. Treatment with trametinib remained efficacious after incubation with A16 and A18 (Fig. 4B). Overall, treatment with investigational drugs can be effective in EOC cells in the presence of AAFs.

### 3.5 Combination treatment with standard‐of‐care drugs is altered in the presence of acellular ascites fluid

**Fig. 5:**
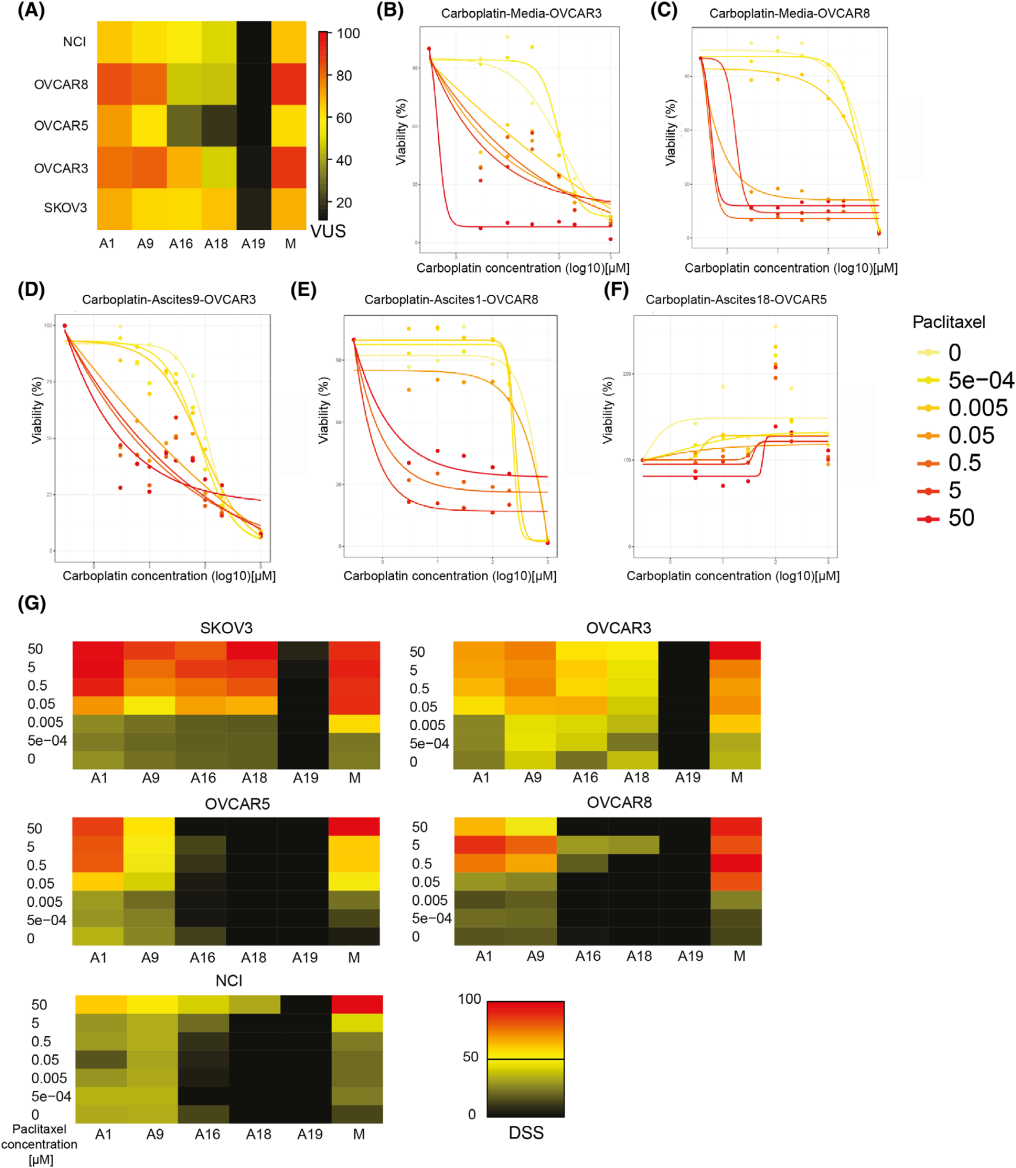
Combination treatment with standard‐of‐care drugs carboplatin and paclitaxel. (A) Heatmap illustrating volume under the surface (VUS) for treatment with a fixed concentration of carboplatin (IC20) and ascending concentrations of paclitaxel in the indicated ovarian cancer cell lines cultured in defined conditions patient‐derived ascites (A), culture media alone (M) (*n* = 3). (B–F) Illustrates cell viability during exposure to standard‐of‐care drugs carboplatin and paclitaxel in ascending concentrations under defined culture conditions. (*n* = 3) (G) Drug sensitivity scores (DSS) for individual cell lines depicting the effect of combination treatment with carboplatin and paclitaxel cultured in media or AAF. VUS and DSS are calculated as stated in Section 2, 1068 and 1308 data points were used for the cell lines OVCAR3 and OVCAR 5, respectively, while 924 data points for SKOV3, NCI and OVCAR8. These counts include replicates.

We sought to assess further the efficacy of combination treatment with SCD carboplatin and paclitaxel on EOC cell lines in the presence of AAF (*n* = 5). As a measure of efficacy for combination experiments, we devised the volume under the surface (VUS) in which the surface is defined along the two directions spanned by the concentrations of the two drugs combined (see Section 2). We found A19 to induce chemoresistance in all cell lines studied. The incubation with A16 and A18 yielded a marked reduction of chemosensitivity in cell lines OVCAR3, OVCAR5, OVCAR8 and NCI, while SKOV3 cells showed a differential response to combination treatment after addition of A9 and A16 (Fig. 5A and Fig. S6). We studied the drug response of individual EOC cell lines treated with ascending doses of the SCD carboplatin and paclitaxel cultured in media alone or with AAF (Fig. 5B–F). We found these combinations to be effective in cell lines cultured in media (Fig. 5B,C). After incubation with A9 and A1, we observed a concentration‐dependent, reduced effect in cell lines OVCAR3 and OVCAR8 (Fig. 5D,E), while the addition of A18 resulted in the induction of chemoresistance in OVCAR5 cells (Fig. 5F). Sensitivity to combination treatment with fixed doses of carboplatin at IC20 dose (as listed in Table S2) and ascending doses of paclitaxel was evaluated using DSS. The combination of SCD was effective in cell lines SKOV3 and OVCAR3, also after incubation with AAF, except for A19. Cell lines OVCAR5, OVCAR8 and NCI were resistant to the combination treatment after adding A16 and A18 (Fig. 5G). These results are consistent with experiments assessing the efficacy of carboplatin and paclitaxel as monotreatment (Fig. 1 and Fig. S3). These data indicate that combinations of SCDs can overcome AAF induced‐chemoresistance but not in all clinical scenarios.

### 3.6 Combination treatment with standard‐of‐care drugs and novel drugs allows nomination of individualised, optimal treatment strategies

Finally, we examined the efficacy of increasing doses of the compounds niclosamide, acetylcysteine, fludarabine, idelalisib, trametinib, nilotinib, ruxolitinib, rapamycin and WP1066 together with defined, cell line‐specific IC_20_ concentrations (see Section 2 and Table S2) of SCDs paclitaxel (Fig. S7) or carboplatin (Fig. S8). In order to accommodate more potent effects, the tested range of concentrations was shifted one order of magnitude compared to when the drugs were used alone on the same conditions. To compare the efficacy of the combinations to paclitaxel or carboplatin alone, we applied combination ratio (CR, see Section 2), nominating the most effective sets of treatment combinations for defined conditions (Table 1). Except for the condition in which SKOV3 cells were cultured with A9, we could nominate a more effective combination treatment, including a novel therapeutic, defined as a CR > 1.

**Table 1:** Overview over the best treatment options for the individual cell lines under defined culture conditions [incubation with patient‐derived ascites (AAF) vs. culture media (M)]. Treatment as a combination of novel therapeutics with standard‐of‐care drugs carboplatin (C) and paclitaxel (P). The lower section of the table informs about combination ratio (CR) with novel therapeutics as compared to monotreatment with carboplatin and paclitaxel.

	AAF1	AAF9	AAF16	AAF18	AAF19	Media
SKOV3	Rapamycin + C	Fludarabine + C	Fludarabine + C	WP1066 + P	Acetylcysteine + C	WP1066 + C
OVCAR3	WP1066 + P	Acetylcysteine + P	Fludarabine + C	Niclosamide + C	Niclosamide + C	Niclosamide + C
OVCAR5	Rapamycin + P	Rapamycin + P	Rapamycin + P	Fludarabine + P	Trametinib + C	WP1066 + C
OVCAR8	Fludarabine + C	Fludarabine + C	Fludarabine + P	Fludarabine + C	Fludarabine + C	Rapamycin + C
NCI	Rapamycin + C	Rapamycin + C	Fludarabine + C	Rapamycin + P	Acetylcysteine + C	Niclosamide + P
SKOV3	1.25	0.97	1.12	1.06	1.14	1.09
OVCAR3	1.03	1.06	1.17	1.25	1.09	1.14
OVCAR5	1.16	1.05	1.12	1.08	1.23	1.15
OVCAR8	1.35	1.3	1.35	1.17	1.14	1.2
NCI	1.3	1.47	1.43	1.17	1.13	1.4

**Fig. 6:**
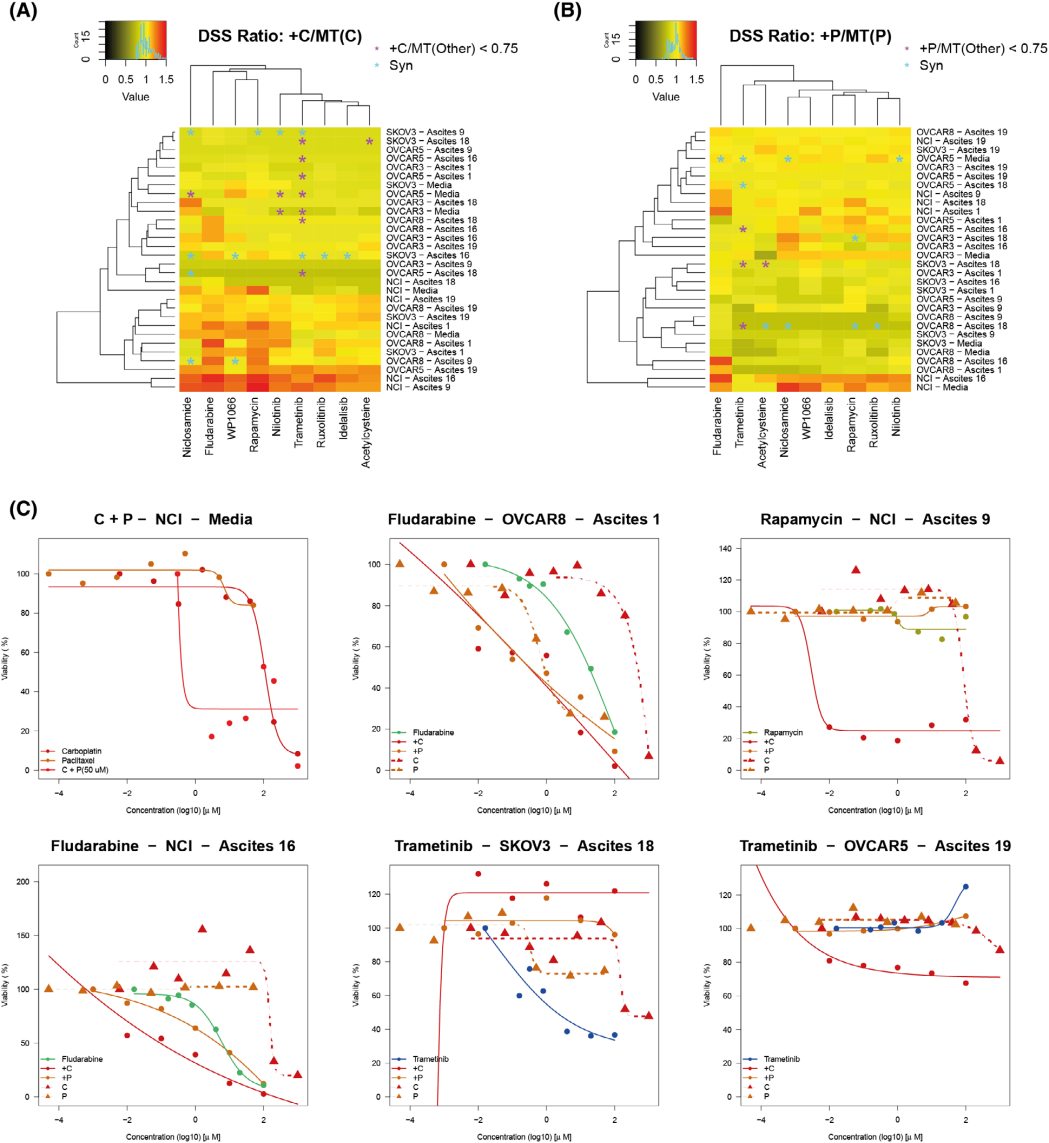
Combination treatment with standard‐of‐care drugs and novel therapeutics. Heatmap depicts drug sensitivity score (DSS) ratio of the combination of (A) carboplatin and (B) paclitaxel with nine novel therapeutics (listed on the *x*‐axes) compared to treatment with standard‐of‐care drugs. Synergy defined as 95th percentile of the zero‐interaction potency (ZIP) values computed using all combinations and all ascites/cell‐line conditions and depicted as blue asterisks, may indicate (a) strong synergy (top 5‐th quantile) or (b) weak interaction (CR < 0.75). Combinations not representing an improvement when compared to standard‐of‐care drugs highlighted with purple asterisks. DSS calculated according to Section 2; 1068 and 1308 data points were used for the cell lines OVCAR3 and OVCAR 5, respectively, while 924 data points for SKOV3, NCI and OVCAR8. These counts include replicates. (C) Dose–response curves depicting data for treatment with single compounds and combinations with standard‐of‐care drugs carboplatin (C) or paclitaxel (P) in cell lines after incubation with patient‐derived ascites or media (*n* = 3).

Niclosamide, rapamycin, fludarabine and WP1066 showed higher efficacy as compared to monotherapy with paclitaxel and carboplatin in about half of the conditions (range between 14 and 18 conditions out of 30 conditions with CR > 1, respectively, for paclitaxel and carboplatin). To identify synergistic effects, we quantified the interactions between the different carboplatin combinations (Fig. 6A) and paclitaxel (Fig. 6B), combined with the nine FDA‐approved novel drugs using the ZIP method. Drug combinations with SCDs carboplatin and paclitaxel showed an analogous number of synergistic interactions (ZIP score above the 95th percentile across all conditions, equal to 43.84 for carboplatin and 22.39 for paclitaxel), including rapamycin, fludarabine and niclosamide. The most pronounced synergistic effect with carboplatin could be observed in the treatment of SKOV3 cells (Fig. 6A). SKOV3 cells cultured in AAF showed enhanced chemosensitivity from all carboplatin combinations except fludarabine and acetylcysteine. In Fig. 6A,B, we also highlighted those combinations not presenting an improvement when compared to the nine FDA‐approved novel drugs (CR < 0.75). The drug trametinib showed the highest number of conditions yielding non‐synergistic interactions with carboplatin (Fig. 6A). To nominate the most effective treatment strategy for individual conditions, we ranked the efficacy of monotherapy and combination treatment with FDA‐approved novel drugs vs. SCD (depicted in Table 2 and Fig. S9). In one‐third of cases, drug combinations of paclitaxel and carboplatin were the most effective. Combinations of paclitaxel with rapamycin and fludarabine ranked highest in another third of conditions. Notably, treatment combinations with trametinib or fludarabine showed highest efficacy in five and in four conditions respectively, where cell cultures had been incubated with AAFs (Table 2, Fig. 6C and Fig. S9).

**Table 2:** Overview over the best treatment options for the individual cell lines under defined culture conditions. Incubation with selected patient‐derived ascites (AAF) vs. culture media (M). Treatment as a combination of novel therapeutics with standard‐of‐care drugs carboplatin (C) and paclitaxel (P). The lower section of the table lists the corresponding DSS.

	AAF 1	AAF9	AAF16	AAF18	AAF19	M
SKOV3	Rapamycin + C	C (100 μm) + P	Fludarabine + C	Trametinib	Acetylcysteine + C	C (100 μm) + P
OVCAR3	C + P (0.05 μm)	C + P (0.05 μm)	C + P (0.05 μm)	C (30 μm) + P	Niclosamide + C	Niclosamide + P
OVCAR5	Rapamycin + P	C + P (0.05 μm)	Trametinib	Trametinib	Trametinib + C	Ruxolitinib + P
OVCAR8	Fludarabine + C	Fludarabine + C	Fludarabine + P	Fludarabine	Fludarabine + C	C (200 μm) + P
NCI	Rapamycin + C	Rapamycin + C	Fludarabine + C	Fludarabine	Acetylcysteine + C	C + P (50 μm)
SKOV3	41.96	33.6	33.58	46.96	14.5	44.52
OVCAR3	48.62	56.77	57.1	42.23	12.6	70.71
OVCAR5	49.57	30.02	49.95	43.05	24.53	61
OVCAR8	55.94	47.33	55	35.42	14.29	61.13
NCI	50	75.42	62.07	45.16	13.99	74.22

To explore whether combinations of therapeutics targeting different pathways were more effective than treatment within one known downstream mode of action, we combined the STAT3 inhibitors nilotinib and niclosamide with each other or nilotinib with the JAK inhibitor WP1066 in OVCAR8 and SKOV3 cells, cultured in media or incubated with A10. In cells cultured in media alone, these combinations were shown to be effective in reducing cell viability. However, in the presence of A10, efficacy was reduced by 50% in OVCAR8 cells treated with nilotinib and WP1066 or by 80% in SKOV3 cells treated with nilotinib and niclosamide (Fig. S10). Overall, we show that treatment strategies can be improved in EOC with the addition of novel compounds to SCDs to reduce AAF‐induced chemoresistance.

## 4 Discussion

Malignant ascites is commonly found in patients diagnosed with advanced‐stage EOC and reoccurs throughout the later stages of the disease. Although the composition of ascites is heterogeneous, the presence of pro‐inflammatory, tumour‐enhancing and chemoresistance‐driving signalling molecules like IL‐6 has been reported in AAF [18, 19, 20, 21, 22, 24, 33]. As such, AAF constitutes a rich liquid microenvironment for EOC cells and facilitates the characteristic early and diffuse trans‐coelomic spread [14, 46]. By assessing viability and drug response to SCD and novel therapeutics, we found that AAF impacts chemosensitivity in EOC cell lines. This is in line with a recent report from Carmi et al., who find IL‐6 levels to correlate with the induction of chemoresistance. In their experiments, chemosensitivity could be restored by treating cells with combinations of platinum compounds and JAK inhibitors [25]. One specific AAF specimen (A19) promoted complete resistance to SCD and novel therapeutics. Interestingly, this sample was collected from a patient with locally advanced carcinosarcoma, a rare tumour known for its aggressiveness and innate chemoresistance [7]. Incubation with A16 and A18 induced chemoresistance to SCD alone and in combinations with OVCAR8, OVCAR5 and OVCAR3. Both these samples were collected from women with common, platinum‐sensitive high‐grade serous carcinoma and may be examples of AAFs that contribute to primary chemoresistance in this disease. In our experiments, we enrich drug testing data as performed by Carmi et al. by characterising the downstream effects active cytokines exert intracellularly.

Phosphorylation profiles of SKOV3 and OVCAR8 cell lines after incubation with AAFs from a total of 20 patients confirmed that the addition of AAF to FCS‐starved cells activates key intracellular pathways such as JAK/STAT, PI3K or MAPK. The addition of FCS containing media may also affect phosphorylation patterns, so all the observed regulation may not be attributable to AAFs. Moreover, high levels of IL‐6 in ascites are shown to exert pro‐inflammatory effects via the activation of STAT3 signalling, and to be associated with poor prognosis and the development of chemoresistance, together with other mechanisms such as acting through exosomes [18, 23, 47]. We report STAT3 to undergo a time‐ and concentration‐dependent activation of signalling after incubation with AAF. To corroborate these data, we studied the effect of IL‐6 receptor blockage during incubation with AAF and observed a clear reduction in STAT3 phosphorylation following treatment with a mAb targeting IL6R, suggesting IL‐6 signalling to mediate activation of STAT3 although we were not able to measure IL‐6 in the AAFs. This is in line with previous studies that reported diminished cytokine and chemokine production, reduced tumour growth, tumour immune infiltrate and angiogenesis following IL‐6 blockage [22]. Early‐phase clinical trials have assessed the efficacy of IL6R‐blocking antibodies in EOC patients, pioneered by the assessment of tocilizumab combined with interferon‐alpha or siltuximab in patients with chemoresistant ovarian cancer, showing promising response rates [22, 44, 45]. Since we confirmed AAF to affect downstream signalling in JAK/STAT, PI3K‐AKT and MEK pathways, and novel anticancer drugs targeting these pathways have been proposed as promising therapeutic strategies in EOC [11, 18, 48] in large clinical trials [49, 50], we expanded our drug library by a total of nine novel therapeutics with FDA approval for other indications for the use in EOC (nilotinib, niclosamide, fludarabine, rapamycin, trametinib, WP1066, acetylcysteine, idelalisib, ruxolitinib). Combinations of these agents with SCDs carboplatin or paclitaxel were more effective than single agents alone. For instance, we identified synergistic effects with paclitaxel and all novel therapeutics but nilotinib. Interestingly, the strongest synergies were seen in the inherently chemoresistant cell line NCI, cultured both with AAF and media alone, ascribing potential re‐sensitisation properties to the novel therapeutics. The MEK inhibitor trametinib has shown efficacy in patients with advanced low‐grade serous carcinoma [50]. Limitations of our study include the fact that we perform drug testing on a finite number of cancer cell lines and with a limited number of drugs and examine a defined panel of intracellular signalling changes. Moreover, as the present paper focuses on intracellular signalling, we did not include a PARP inhibitor to our drug portfolio. PARP inhibitors have previously been shown to be effective in EOC cell lines, harbouring mutations in the *BRCA1* gene as well as in cells that are *BRCA1/2* wild‐type and *MYC* amplification and genome‐wide rearrangements are introduced as predictive factors [51] together with established markers for homologous repair deficiency [52].

## 5 Conclusion

In conclusion, we show that AAFs possess the ability to alter drug responses to SCD as well as to novel therapeutics through the activation of downstream signalling pathways in EOC cells. Our results underscore the importance of future efforts in characterising the acellular and cellular components in ascites and possibly utilising AAF as a source of predictive and prognostic biomarkers informing personalised treatment choices.

## Conflict of interest

The authors declare no conflict of interest.

## Author contributions

KT initiated the study. KT and AU supervised the work. AU and AC designed the analysis. LE, JL and L‐LF‐L produced the data. KT, LB and KB contributed data/samples/analysis tools. AC, AU, LE, AG, L‐LF‐L, LB and KB performed analysis. AC, KB and AU drafted the manuscript. KB, AU, AC and KT wrote the paper. All the authors read and approved the final version of the paper.

### Peer review

The peer review history for this article is available at https://www.webofscience.com/api/gateway/wos/peer‐review/10.1002/1878‐0261.13726.

## Supporting information


**Fig. S1.** Concentration‐dependent dynamics of intracellular signalling in ovarian cancer cell lines after incubation with patient‐derived acellular ascites fluid.
**Fig. S2.** Temporal dynamics of intracellular signalling in ovarian cancer cell lines after incubation with patient‐derived ascites.
**Fig. S3.** Concentration‐responses to treatment with paclitaxel and carboplatin of cell lines OVCAR3, OVCAR5 and NCI after incubation with patient‐derived ascites.
**Fig. S4.** Intracellular pathway activation following incubation of OVCAR8 cells with patient‐derived ascites.
**Fig. S5.** Dose response curves for the treatment with novel therapeutics of five ovarian cancer cell lines.
**Fig. S6.** Dose response curves for combination treatment with standard of care drugs in the presence of five different acellular ascites fluid.
**Fig. S7.** Dose response curves for combination treatment with novel therapeutics and paclitaxel at its IC20 (after incubation with five different patient‐derived ascites or media).
**Fig. S8.** Dose response curves for combination treatment with novel therapeutics and carboplatin at its IC20 (after incubation with five different patient‐derived ascites or media).
**Fig. S9.** Dose response curves for treatment with novel therapeutics alone or together with standard‐of‐care drugs carboplatin or paclitaxel in cell lines after incubation with patient‐derived ascites or media alone.
**Fig. S10.** Combination treatment with novel therapeutics.
**Table S1.** Overview over clinicopathological data of patients included (*n* = 20).
**Table S2.** IC20 (in μm or m) of standard‐of‐care drugs Paclitaxel and Carboplatin in the indicated cell lines derived from experiments in Figs 1 and 5 and Fig. S3.
**Table S3.** Overview over phospho‐antibodies used in the flow‐cytometry analyses.
**Table S4.** Summary statistics for drug sensitivity score (DSS).

## Data Availability

The data that support the findings of this study are available on request from the corresponding author. The data are not publicly available due to privacy or ethical restrictions.
