# Supplementary material for: Patient‐derived acellular ascites fluid affects drug responses in ovarian cancer cell lines through the activation of key signalling pathways
Source: Mol Oncol. 2024 Sep 8;19(1):81–98. doi: 10.1002/1878-0261.13726 (PMC11705723; doi:10.1002/1878-0261.13726)
Supplement: Supplementary file 1 — Fig. S1. Concentration‐dependent dynamics of intracellular signalling in ovarian cancer cell lines after incubation with patient‐derived acellular ascites fluid. Fig. S2. Temporal dynamics of intracellular signalling in ovarian cancer cell lines after incubation with patient‐derived ascites. Fig. S3. Concentration‐responses to treatment with paclitaxel and carboplatin of cell lines OVCAR3, OVCAR5 and NCI after incubation with patient‐derived ascites. Fig. S4. Intracellular pathway activation following incubation of OVCAR8 cells with patient‐derived ascites. Fig. S5. Dose response curves for the treatment with novel therapeutics of five ovarian cancer cell lines. Fig. S6. Dose response curves for combination treatment with standard of care drugs in the presence of five different acellular ascites fluid. Fig. S7. Dose response curves for combination treatment with novel therapeutics and paclitaxel at its IC20 (after incubation with five different patient‐derived ascites or media). Fig. S8. Dose response curves for combination treatment with novel therapeutics and carboplatin at its IC20 (after incubation with five different patient‐derived ascites or media). Fig. S9. Dose response curves for treatment with novel therapeutics alone or together with standard‐of‐care drugs carboplatin or paclitaxel in cell lines after incubation with patient‐derived ascites or media alone. Fig. S10. Combination treatment with novel therapeutics. Table S1. Overview over clinicopathological data of patients included (n = 20). Table S2. IC20 (in μm or m) of standard‐of‐care drugs Paclitaxel and Carboplatin in the indicated cell lines derived from experiments in Figs 1 and 5 and Fig. S3. Table S3. Overview over phospho‐antibodies used in the flow‐cytometry analyses. Table S4. Summary statistics for drug sensitivity score (DSS). [file MOL2-19-81-s001.pdf]

Supplementary Material

1. Supplementary Tables

Patient-ID	Age at diagnosis (years)	FIGO Stage	Ca125 levels IU/ml	Histological subtype	Macroscopic resection status
1	63	FIGO IIIC	451	HGSC	R0
2	57	FIGO IIIC	961	HGSC	R0
3	69	FIGO IIIC	46000	serous	R1
4	52	FIGO IIIC	535	serous	R1
5	54	FIGO IIIC	2784	HGSC	R1
6	40	FIGO IIIC	497	HGSC	R2
7	55	FIGO IIIC	1534	HGSC	R1
8	68	FIGO IIB	492	HGSC	R0
9	42	FIGO IV	440	HGSC	R1
10	70	FIGO IIIC	655	HGSC	R2
11	51	FIGO IA	23	mucinous	R0
12	68	FIGO IV	427	clear cell	R2
13	76	FIGO IIIC	2962	serous	R2
14	58	FIGO IIIC	2320	HGSC	R2
15	66	FIGO IIIC	2237	HGSC	R2
16	63	FIGO IIIC	966	HGSC	R2
17	50	FIGO IIIC	6000	LGSC	R1
18	57	FIGO IIIC	158	serous	R2
19	65	FIGO IIB	531	carcinosarcoma	R0
20	69	FIGO IIIC	656	HGSC	R1

Supplementary Table 1. Overview over clinicopathological data of patients included (n=20). Abbrevations: FIGO The International Federation of Gynecology and Obstetrics; HGSC high-grade serous cancer, LGSC low-grade serous cancer; serous – not classified R0 no macroscopic rest tumour, R1 < 2 cm rest tumor, R2 > 2 cm rest tumor at primary surgery.

	SKOV 3	OVCAR 3	OVCAR 5	OVCAR 8	NCI
Paclitaxel (µM)	0.0024	0.002	0.034	0.004	50
Carboplatin (M)	82	33	96	215	62

Supplementary Table 2. IC20 (in µM or M) of standard-of-care drugs Paclitaxel and Carboplatin in the indicated cell lines derived from experiments in Figure 1, Figure 5 and Supplementary Figure 1.

	Concentration antibody (µl)	FACS buffer (µl)	Producer/ CAT#
IgG kappa	5	20	BD/557783
p-STAT1 (pS727)	5	20	BD/560190
p-STAT1 (pY701)	5	20	BD/512597
p-STAT3 (pY705)	4	21	BD/557815
p-STAT5 (pY694)	5	20	BD/612597
p-AKT (pT308)	5	20	CST/3375
p-AKT (pS473)	2	23	CST/4075
p-S6RP (pS235/236)	1.5	23.5	CST/4851
p-MEK1 (pS298)	5	20	BD/560043
p-ERK 42/44	1	124	CST/4357
p-P38 MAPK (pT180/Y182)	1	49	CST/4552
p-MAPKAPK-2 (pT334)	1	24	CST/4320
p-JAK-2*	1	200	CST/8082S
p-STAT6(pY641)	5	20	BD/612601
p-NF-κB (pS529)	1.5	23.5	BD/558422
p-NF-κB (pS536)	2.5	22.5	CST/4887
p-ATF-2 (pT71)	1	24	SC/8398
p-HSP27(pS78)*	1	124	CST/2405
p-SAPK/JNK	2	23	CST/9257
p-HISTONE H3 (pS10)	1.5	48.5	CST/9716
p-CDC-2 (pT161)*	1	125	CST/9114
p-RB (pS807/pS811)	2	23	BD/558590
p-GSK3-alpha (pS21)*	1	125	CST/9316
p-GSK3-beta (pS9)*	1	125	CST/9336S
p-VASP (pS239)*	1	500	CST/3114
p-CaMKII (pT286)*	1	125	CST/3361
p-PKD/PKC µ (pS744/748)*	1	500	CST/2054
p-VAV (pY174)*	1	500	SC/16408-R
p-PLC gamma 1 (pT783)*	1	250	CST/2821
p-Beta-Catenin (pS45)*	1	250	CST/9564
p-VASP (pS157)*	1	500	CST/3111
p-VIMENTIN*	1	125	AC/52942

Supplementary Table 3. Overview over phospho-antibodies used in the flow-cytometry analyses. Producer full name: BD=BD Bioscience, CST= Cell signaling technologies, SC=Santa Cruz, AC= Abcam, * unconjugated antibody

Drug Name	2-way ANOVA DSS vs Culture	2-way ANOVA DSS vs Cell Line	Avg DSS (Media)	Avg DSS (Ascites)
**Paclitaxel**	6.4*10^(-5)	1.2*10^(-4)	35.47	14.99
**Carboplatin**	6.3*10^(-3)	0.2	16.4	11.9
**Acetylcysteine**	0.4	0.5	8.62	2.56
**Niclosamide**	5.8*10^(-11)	0.2	40.2	6.87
**Fludarabine**	2.4*10^(-4)	0.01	18.55	21.56
**Idelalisib**	1.7*10^(-3)	0.3	16.54	5.58
**Trametinib**	2.3*10^(-5)	0.02	33.11	18.76
**Nilotinib**	4.2*10^(-6)	0.07	41.68	13.85
**Ruxolitinib**	4.8*10^(-4)	0.02	13.59	4.62
**Rapamycin**	3.1*10^(-4)	0.6	31.11	11.95
**WP1066**	1.6*10^(-8)	0.7	43.68	14.04

Supplementary table 4. Summary statistics for drug sensitivity score (DSS). Individual DSS are calculated as stated in the Materials and Methods section, 1068 and 1308 data points were used for the cell lines OVCAR3 and OVCAR 5, respectively, while 924 data points for SKOV3, NCI and OVCAR8. These counts include replicates.

2. Supplementary figures

Supplementary Figure 1

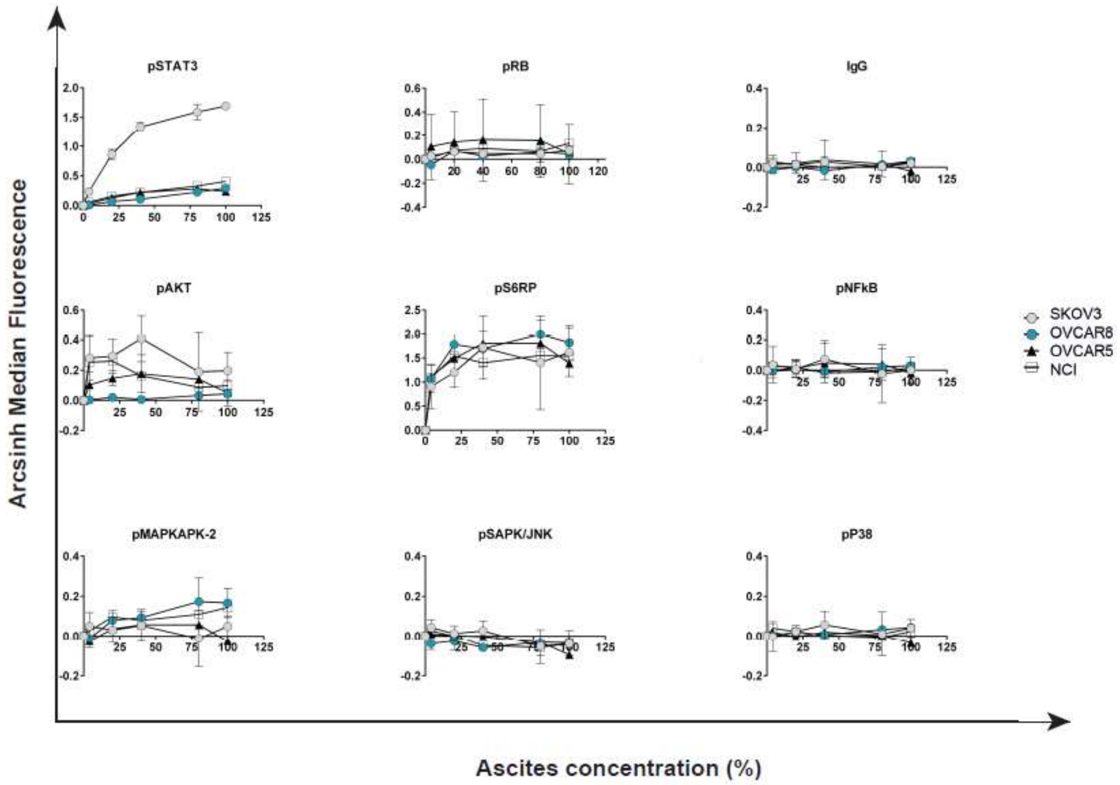

Supplementary Figure 1. Concentration-dependent dynamics of intracellular signalling in ovarian cancer cell lines after incubation with patient-derived acellular ascites fluid. Ovarian cancer cell lines SKOV3, OVCAR8, OVCAR5 and NCI treated with ascending concentrations of patient-derived ascites (A15) (at concentrations of 25-50-75% added to culture media). Phosphorylation status of key intracellular proteins STAT3, RB, Akt, S6RP, NFκB, MAPKAPK-2, SAPK/JNK, and P38 was assessed by phospho flow. IgG served as a control. Graphs depict median fluorescence intensity for individual proteins after treatment with ascending concentrations of patient-derived ascites.

Supplementary Figure 2

A.

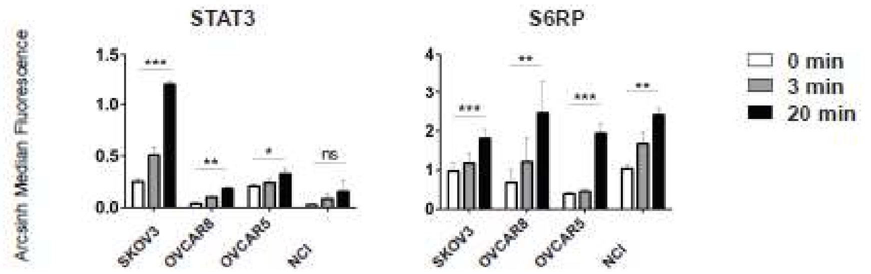

B.

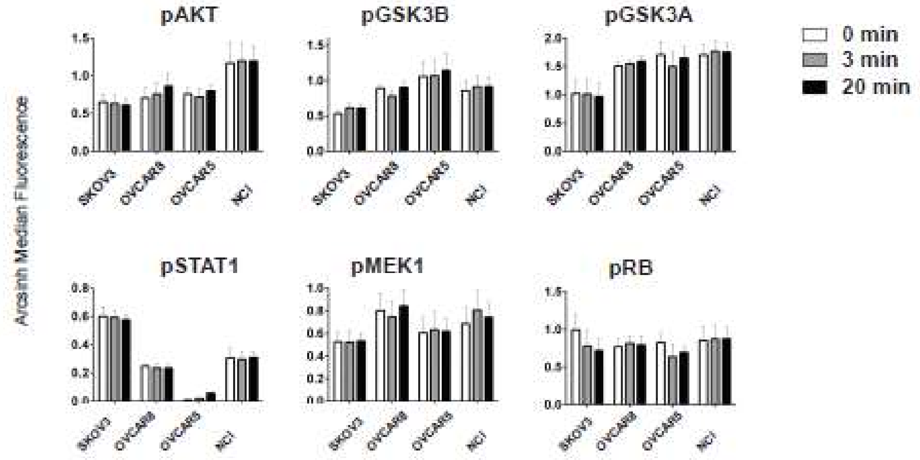

C.

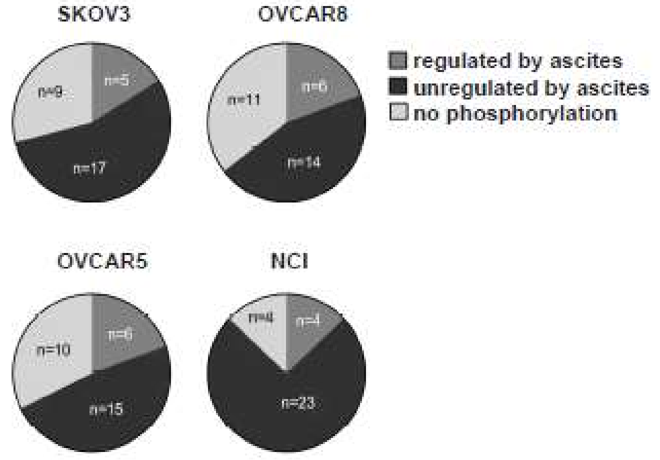

Supplementary Figure 2. Temporal dynamics of intracellular signaling in ovarian cancer cell lines after incubation with patient-derived ascites. (A) Phosphorylation status of STAT3 and S6RP after incubation with patient-derived ascites (A10) for 0, 3 or 20 minutes in cell lines SKOV3, OVCAR8 OVCAR5 and NCI (One-way ANOVA test ***P<0.001, **P<0.005, *P<0.05, ns = not significant). **(B)** Phosphorylation status for intracellular proteins AKT, GSK3B, GSK3A, STAT1, MEK1 and RB. **(C)** Overall effects of stimulation with A10 on protein phosphorylation in individual cell lines. The 31 targets included in the analysis as depicted in Figure 2A.

Supplementary Figure 3

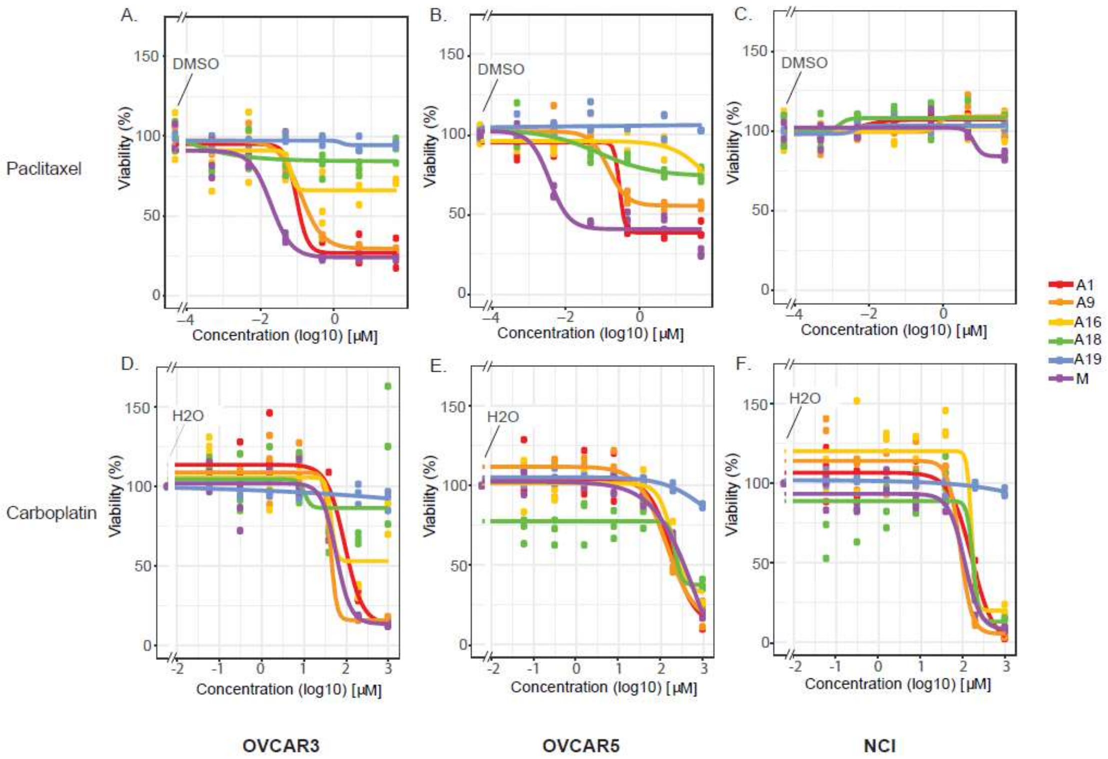

Supplementary Figure 3. Concentration-responses to treatment with paclitaxel and carboplatin of cell lines OVCAR3, OVCAR5 and NCI after incubation with patient-derived acellular ascites.

Concentration-responses to treatment with paclitaxel **(A,B,C)** and carboplatin **(D,E,F)** in the ovarian cell line OVCAR3, OVCAR5, NCI incubated in culture media alone (M) or with five different patient-derived acellular ascites fluids (A). Concentration-responses have been fitted to a 4-parameters log-logistic model for which the lower bound was set to zero.

Supplementary Figure 4

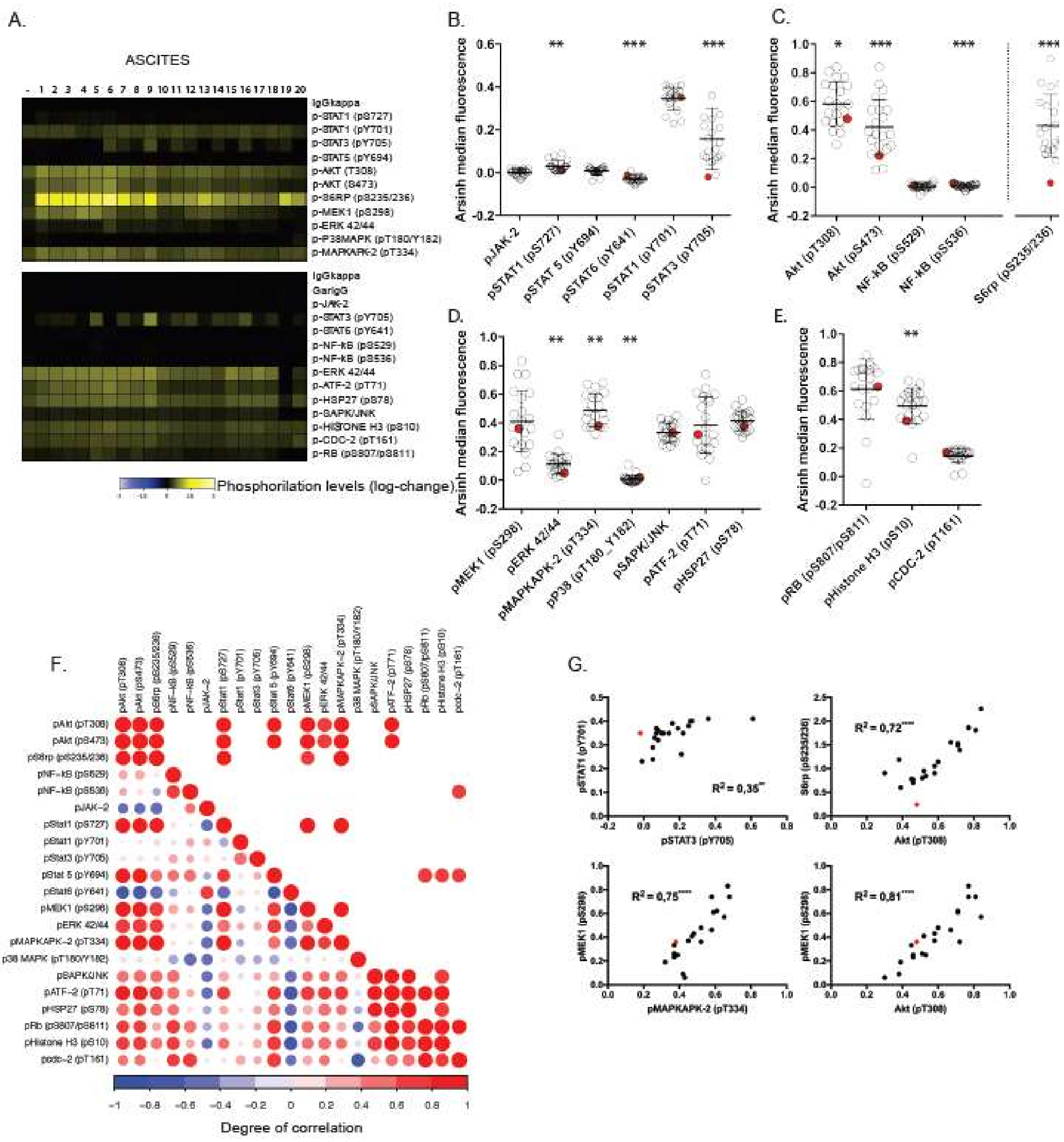

Supplementary Figure 4. Intracellular pathway activation following incubation of OVCAR8 cells with patient-derived ascites.

(A) Heatmap illustrating individual phospho-signalling profiles for OVCAR8 cells after incubation with acellular ascites fluid (n=20, labeled #1-20) or media (-). The target pSTAT3(pY705) was included in all three panels that were run. IgG served as a control and data are normalized to the first row in each heatmap (representing different panel runs).

(B-E) Variation in phosphorylation levels in downstream effectors in OVCAR8 cells (red dots represent signal in cells in media alone used as control). (B) JAK/STAT, (C) PI3K/AKT, (D) MAPK/MEK/ERK pathways, and (E) other intracellular markers. t-tests comparing the mean fluorescence values in OVCAR8 cells versus control are shown, mean and SD are plotted. The multiple tests are corrected with Holm method. P-value ranges: *$P \leq 0.05$, **$P \leq 0.01$, ***$P \leq 0.001$. (F) Correlation matrix illustrating the degree of correlation among protein phosphorylation levels of intracellular effectors after incubation with AAF (n= 20). The upper part only presents the significant values, corrected for multiplicity by the Holm Method ($p<0.05$) (G) Correlation coefficients for univariate correlation of phosphorylation levels of selected key downstream effectors. Red dots indicate the phosphorylation levels measured in cells cultured in media alone.

SKOV3

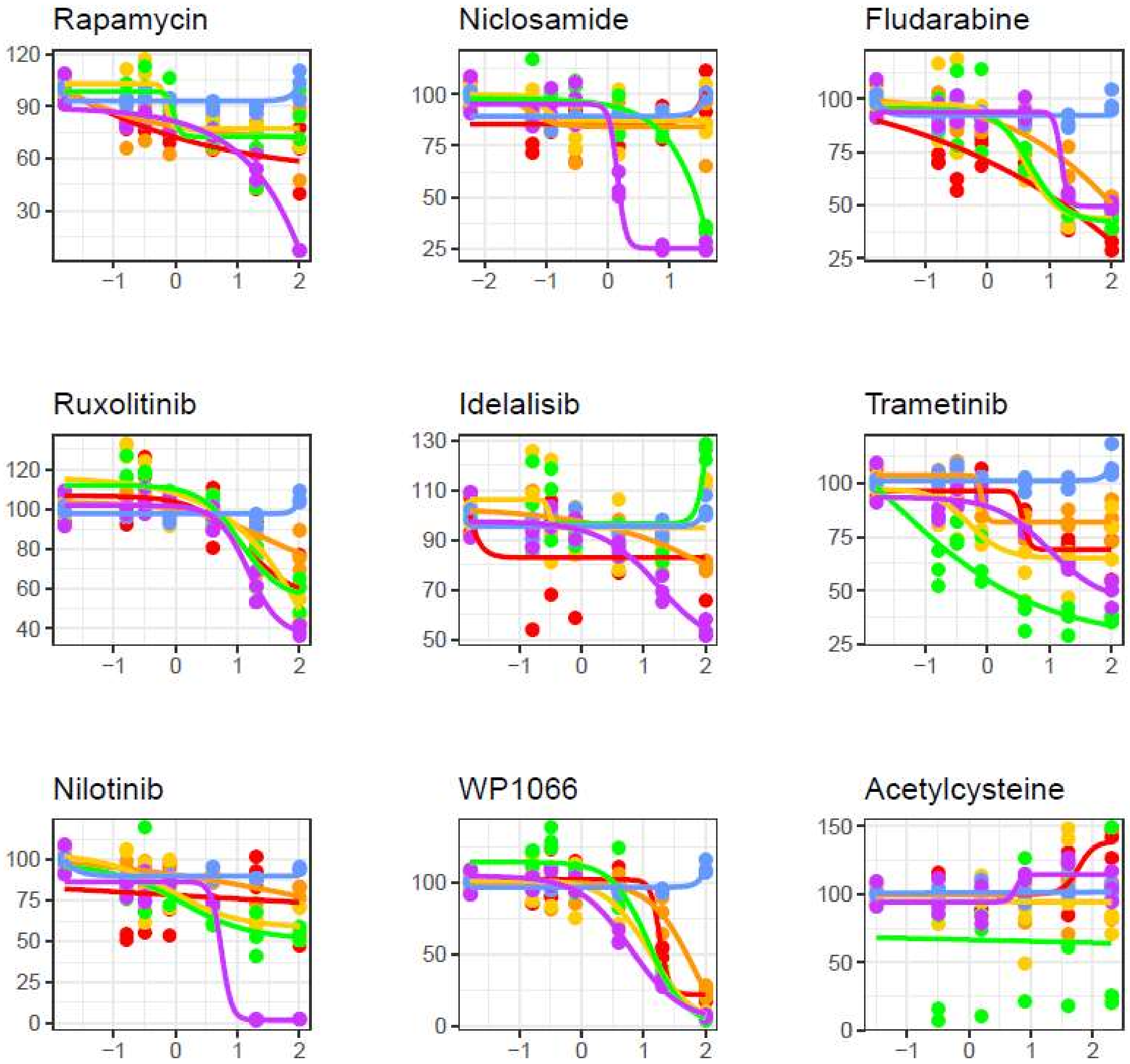
Rapamycin
Niclosamide
Fludarabine
Ruxolitinib
Idelalisib
Trametinib
Nilotinib
WP1066
Acetylcysteine

OVCAR3

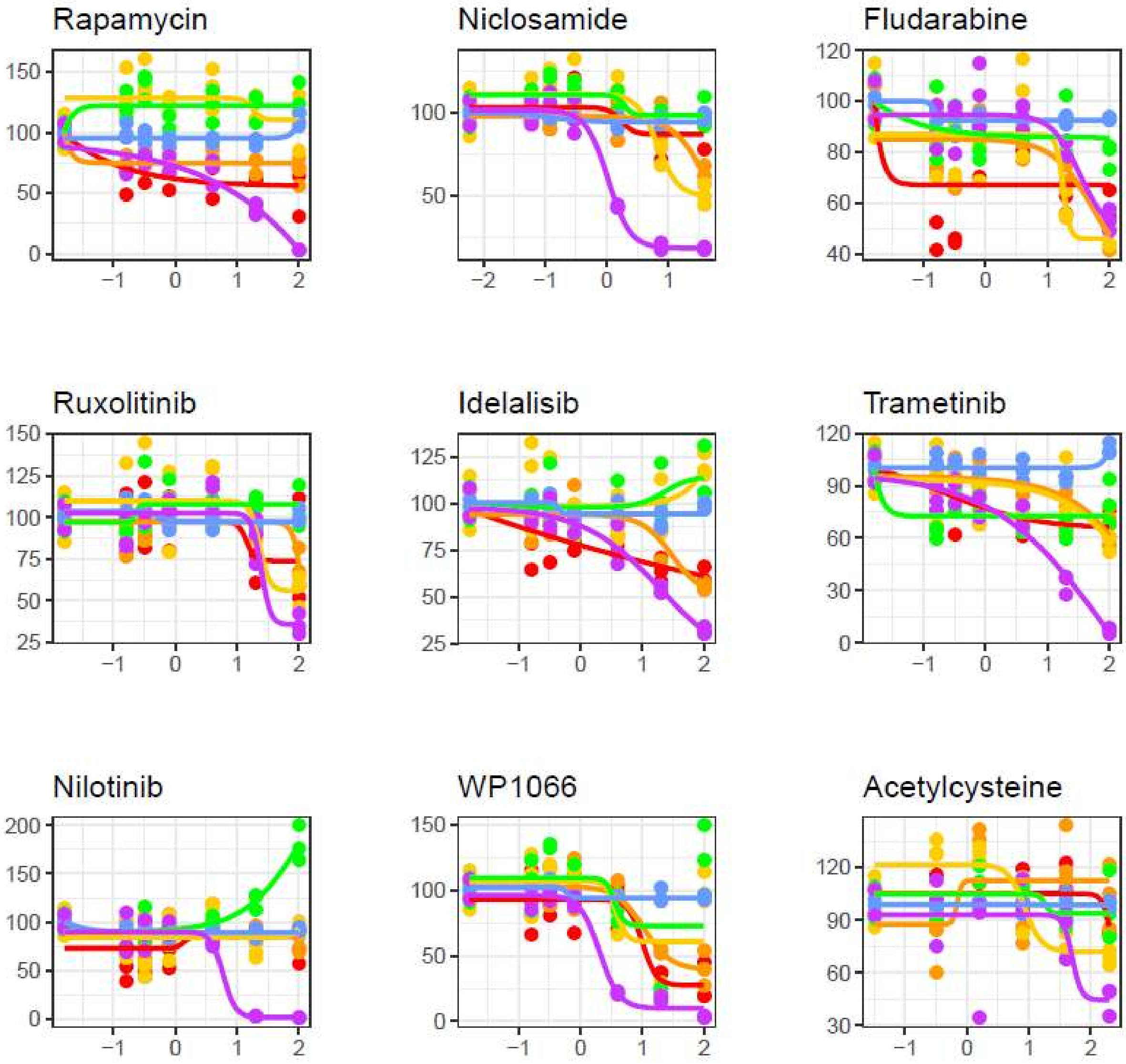

Rapamycin
150 100 50 0
−1 0 1 2
Niclosamide
100 50
−2 −1 0 1
Fludarabine
120 100 80 60 40
−1 0 1 2
Ruxolitinib
150 125 100 75 50 25
−1 0 1 2
Idelalisib
125 100 75 50 25
−1 0 1 2
Trametinib
120 90 60 30 0
−1 0 1 2
Nilotinib
200 150 100 50 0
−1 0 1 2
WP1066
150 100 50 0
−1 0 1 2
Acetylcysteine
120 90 60 30
−1 0 1 2

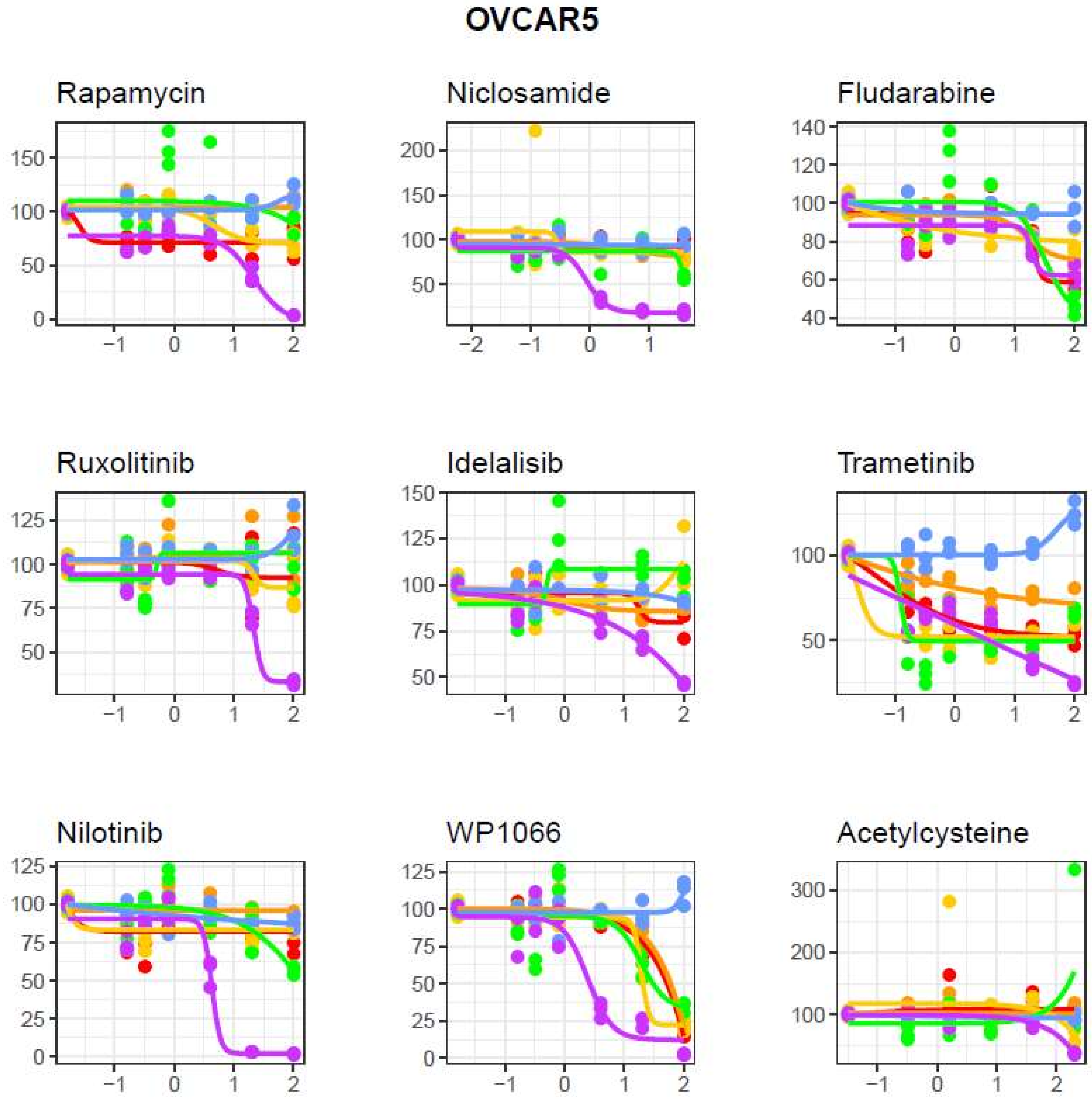

OVCAR5
Rapamycin
Niclosamide
Fludarabine
Ruxolitinib
Idelalisib
Trametinib
Nilotinib
WP1066
Acetylcysteine

OVCAR8

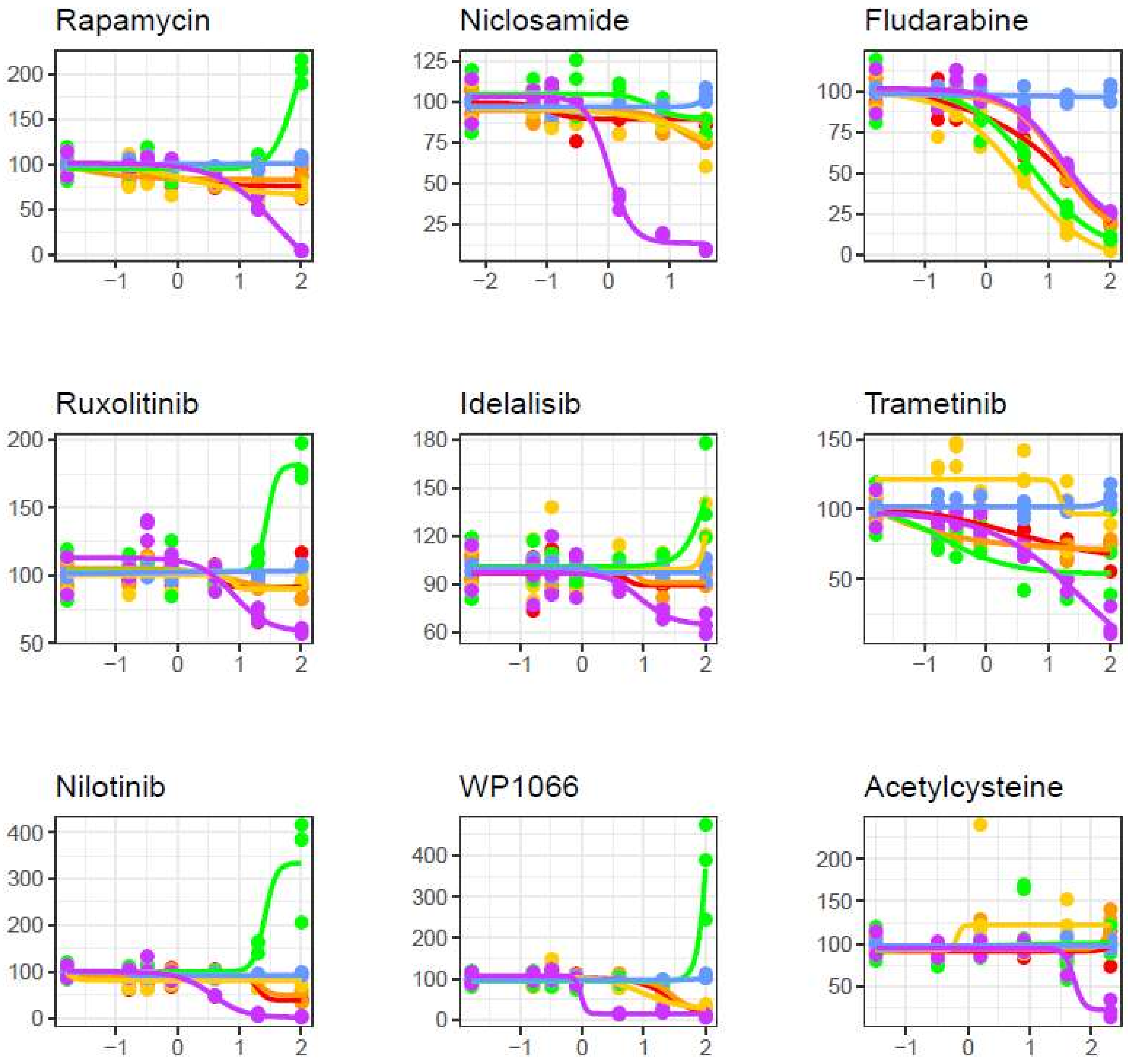
Rapamycin
200
150
100
50
0
−1
0
1
2
Niclosamide
125
100
75
50
25
−2
−1
0
1
Fludarabine
100
75
50
25
0
−1
0
1
2
Ruxolitinib
200
150
100
50
−1
0
1
2
Idelalisib
180
150
120
90
60
−1
0
1
2
Trametinib
150
100
50
−1
0
1
2
Nilotinib
400
300
200
100
0
−1
0
1
2
WP1066
400
300
200
100
0
−1
0
1
2
Acetylcysteine
200
150
100
50
−1
0
1
2

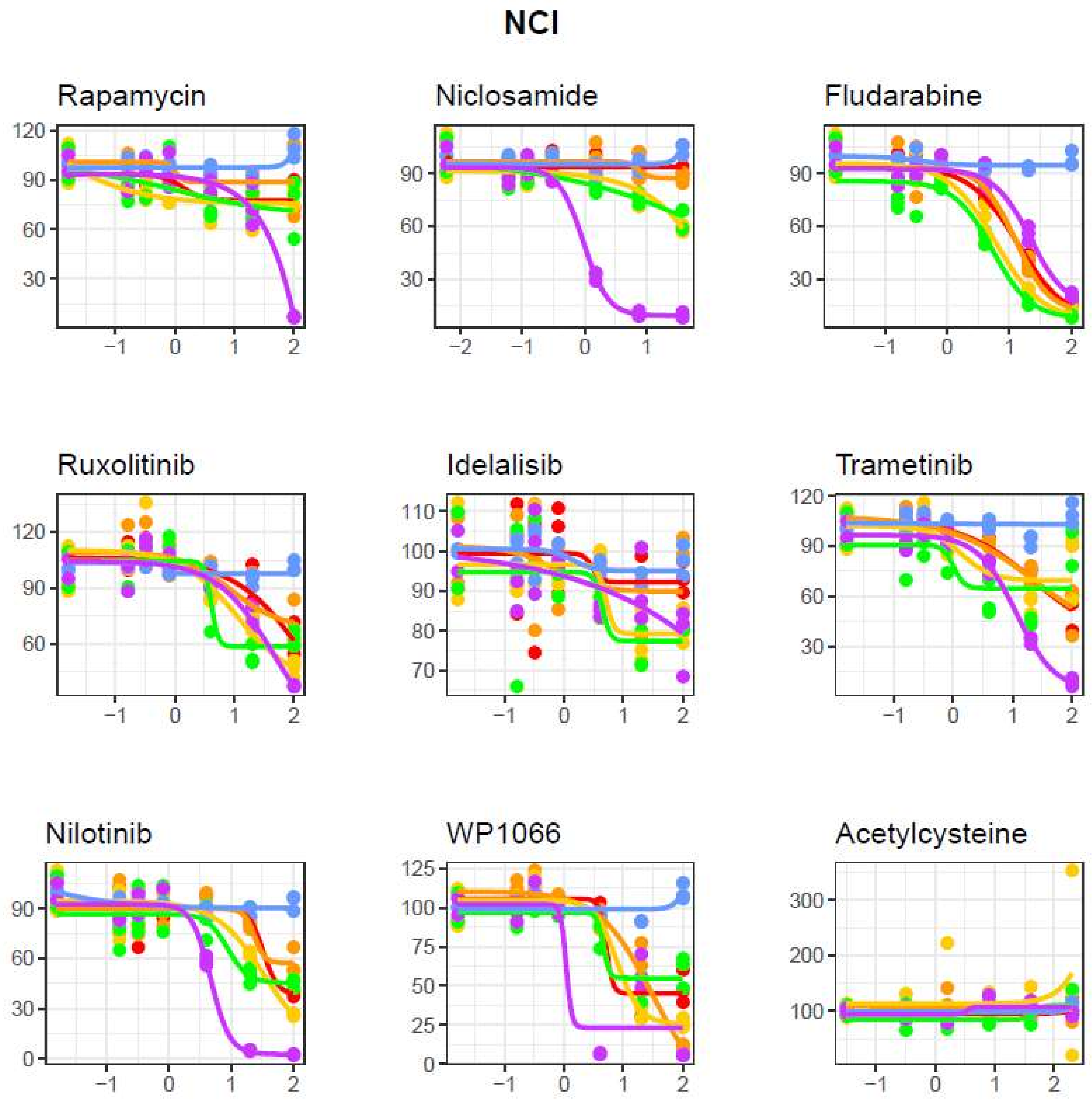

Supplementary Figure 5. Monotherapy with novel therapeutics (X-axes depict the log10 of the drug concentration whilst Y-axes is the relative viability) after incubation with five different patient-derived ascites fluids or media.

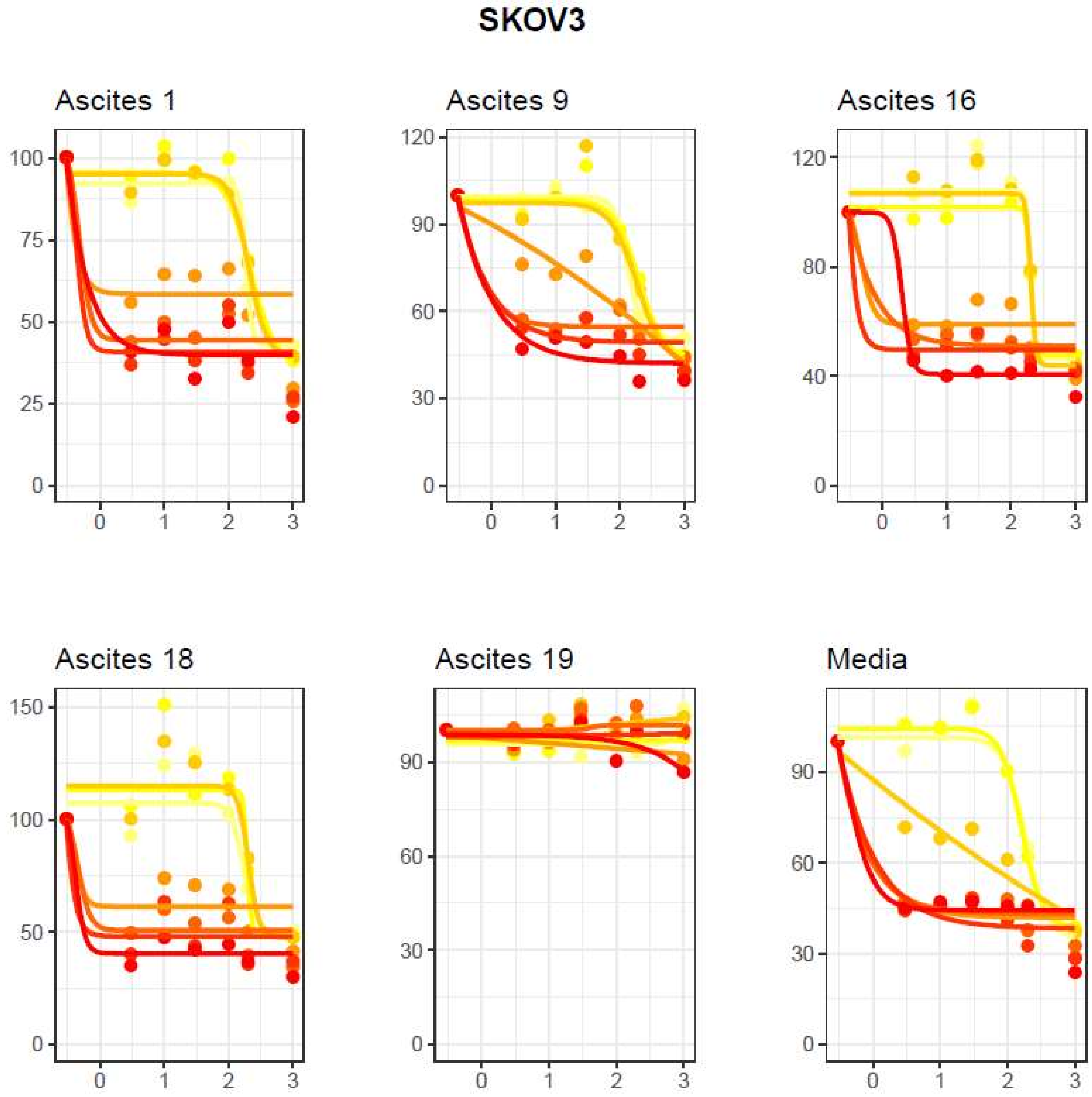
SKOV3
Ascites 1
Ascites 9
Ascites 16
Ascites 18
Ascites 19
Media

OVCAR3

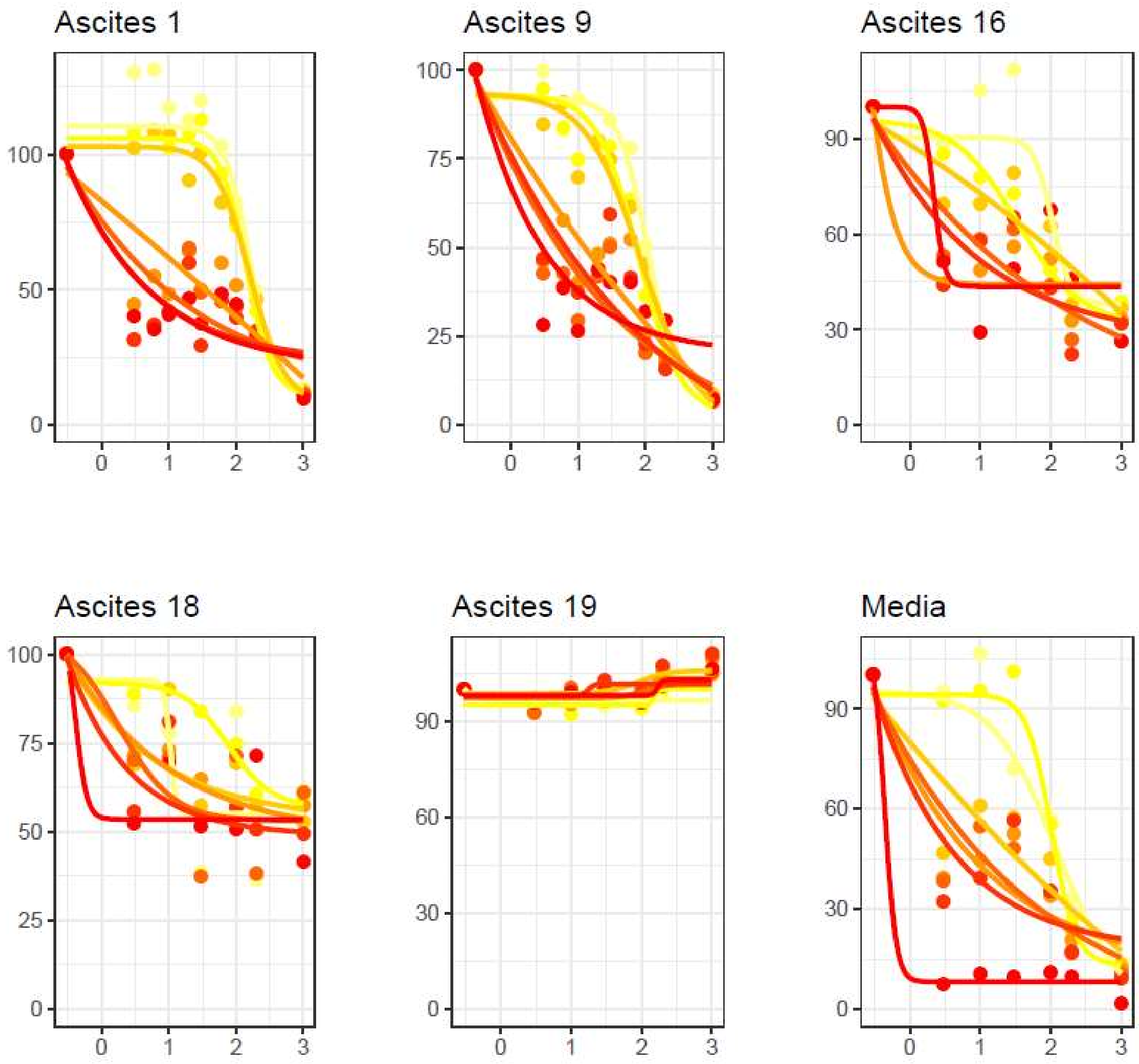

Ascites 1
Ascites 9
Ascites 16
Ascites 18
Ascites 19
Media
100
75
50
25
0
90
60
30
0
1
2
3

OVCAR5

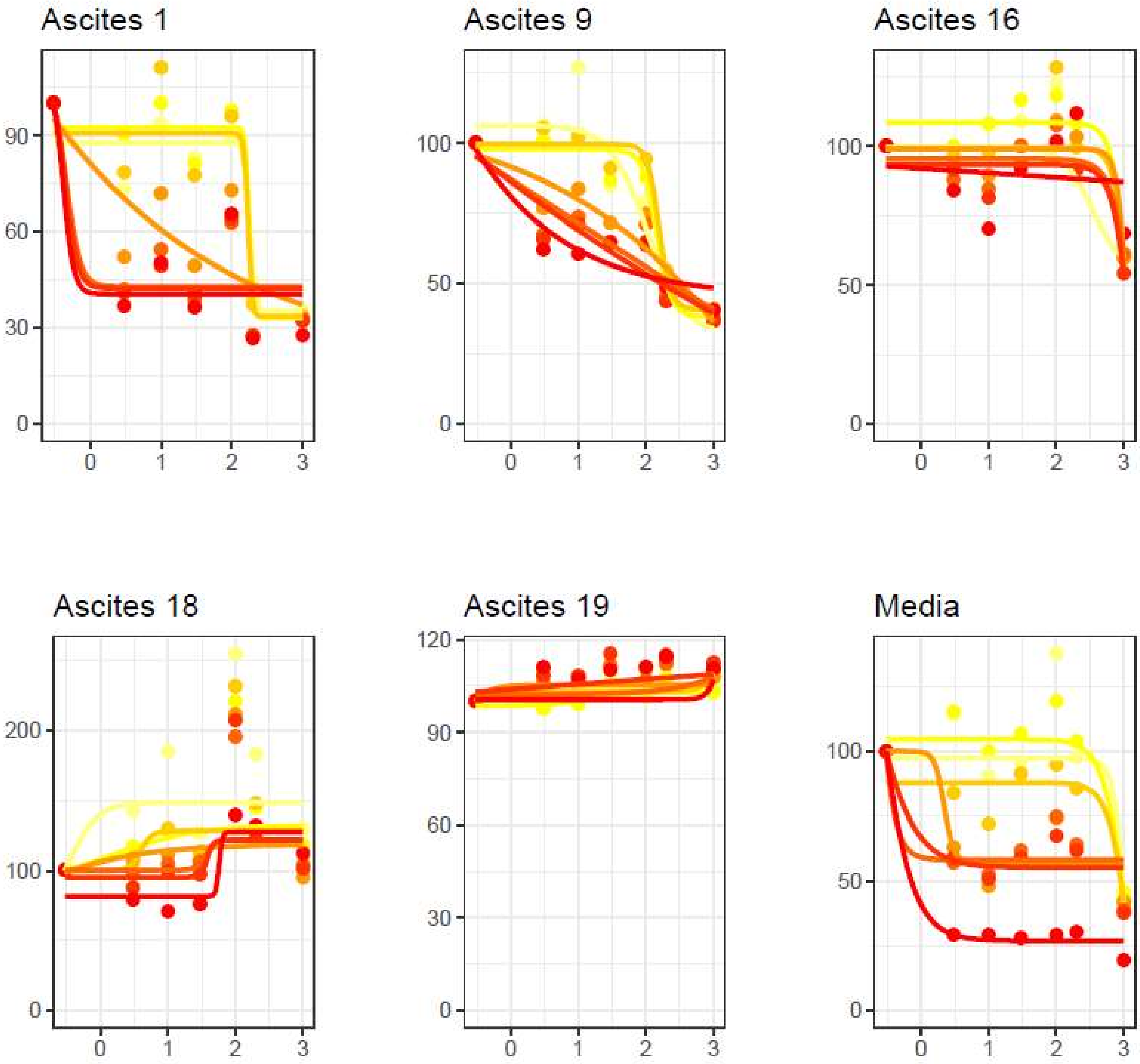

OVCAR8

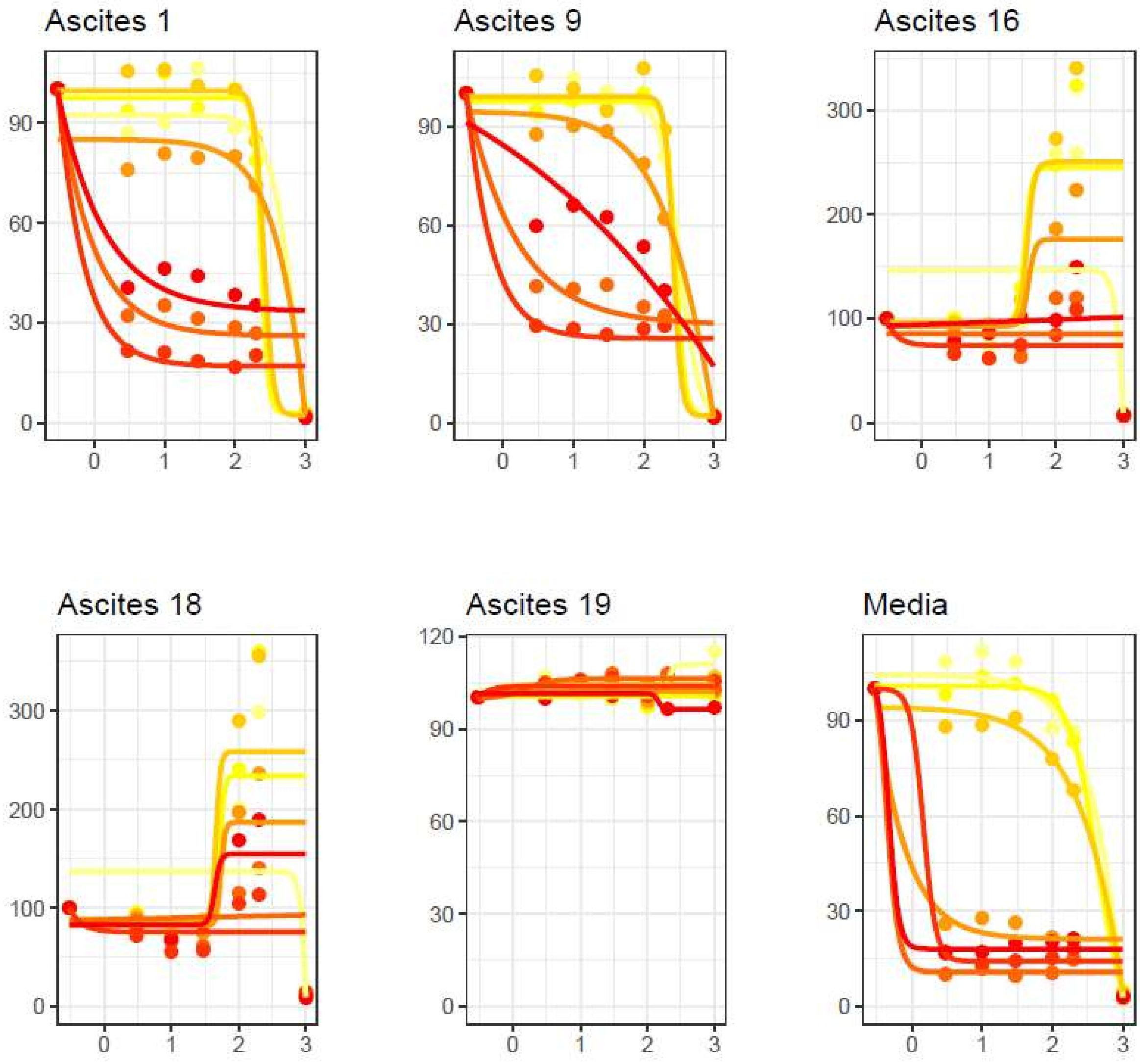

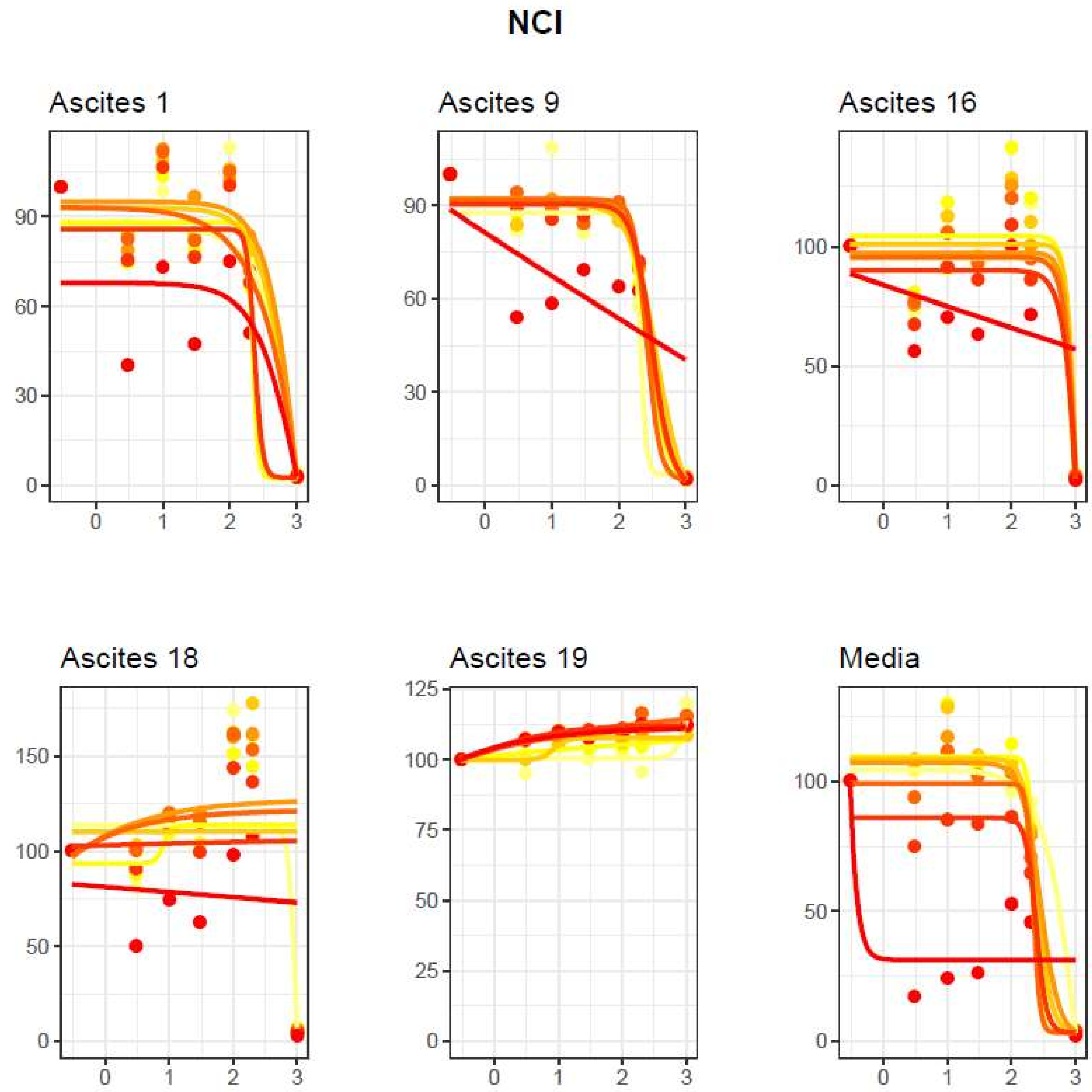

Supplementary Figure 6. Combinations of paclitaxel and carboplatin (X-axes depict the log10 of the concentration of carboplatin whilst Y-axes is the relative viability) after incubation with five different patient-derived ascites fluids or media. Colors ranging from yellow to red indicate increasing concentrations of paclitaxel with red being the highest concentration.

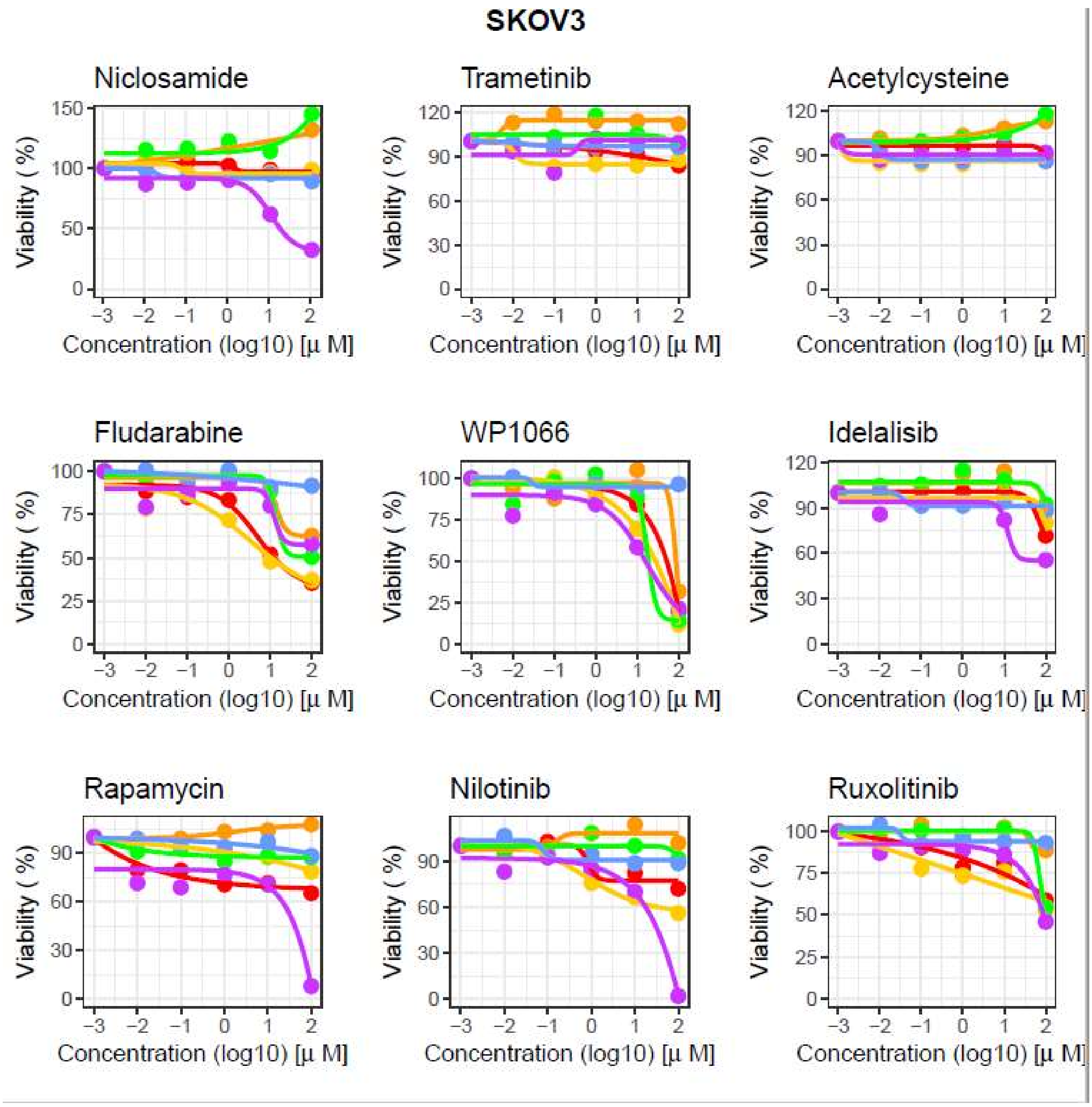
SKOV3
Niclosamide
Trametinib
Acetylcysteine
Fludarabine
WP1066
Idelalisib
Rapamycin
Nilotinib
Ruxolitinib
Viability (%)
Concentration (log10) [μ M]

OVCAR3

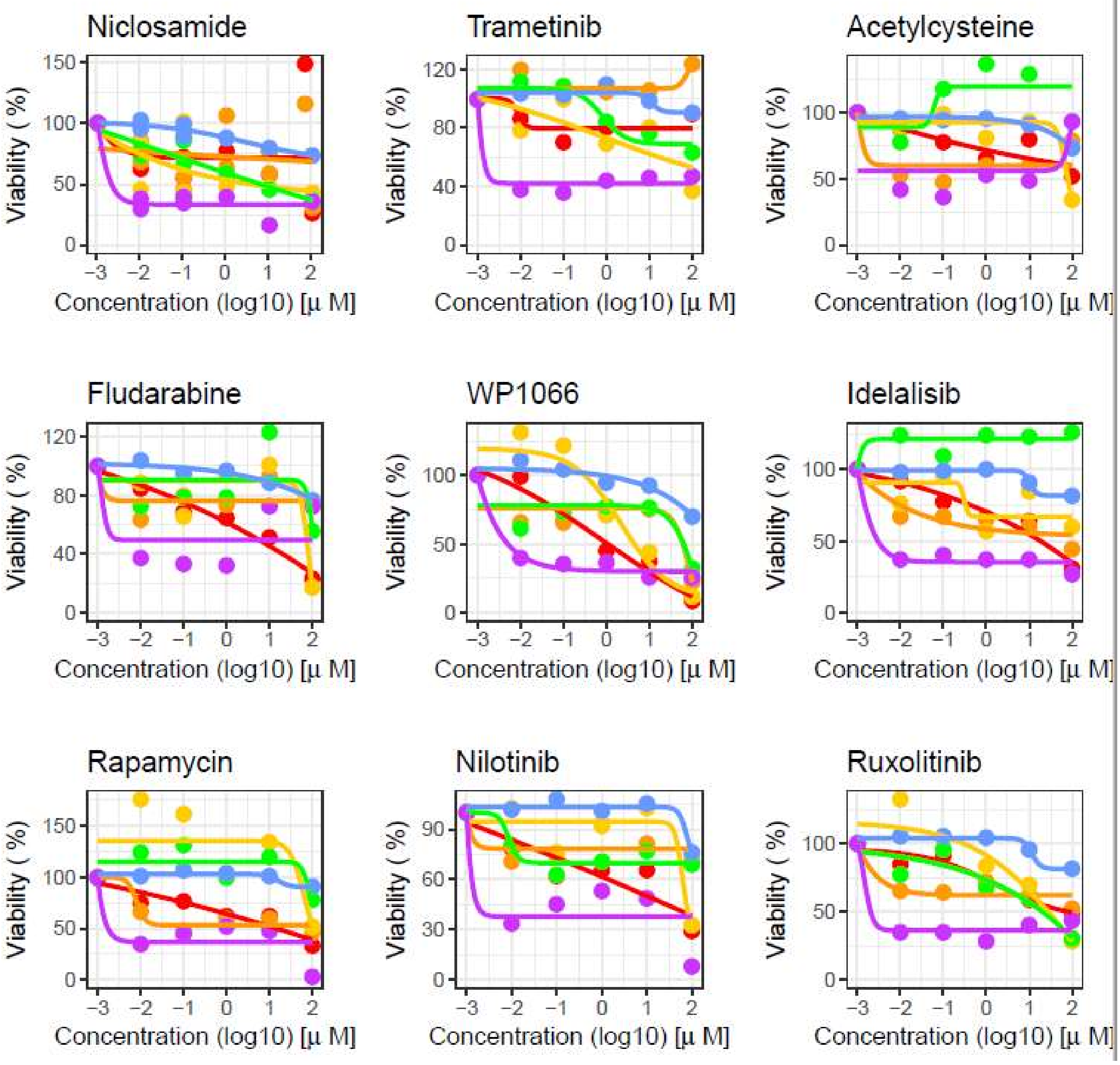

Niclosamide
Trametinib
Acetylcysteine
Fludarabine
WP1066
Idelalisib
Rapamycin
Nilotinib
Ruxolitinib
Viability (%)
Concentration (log10) [μ M]

OVCAR5

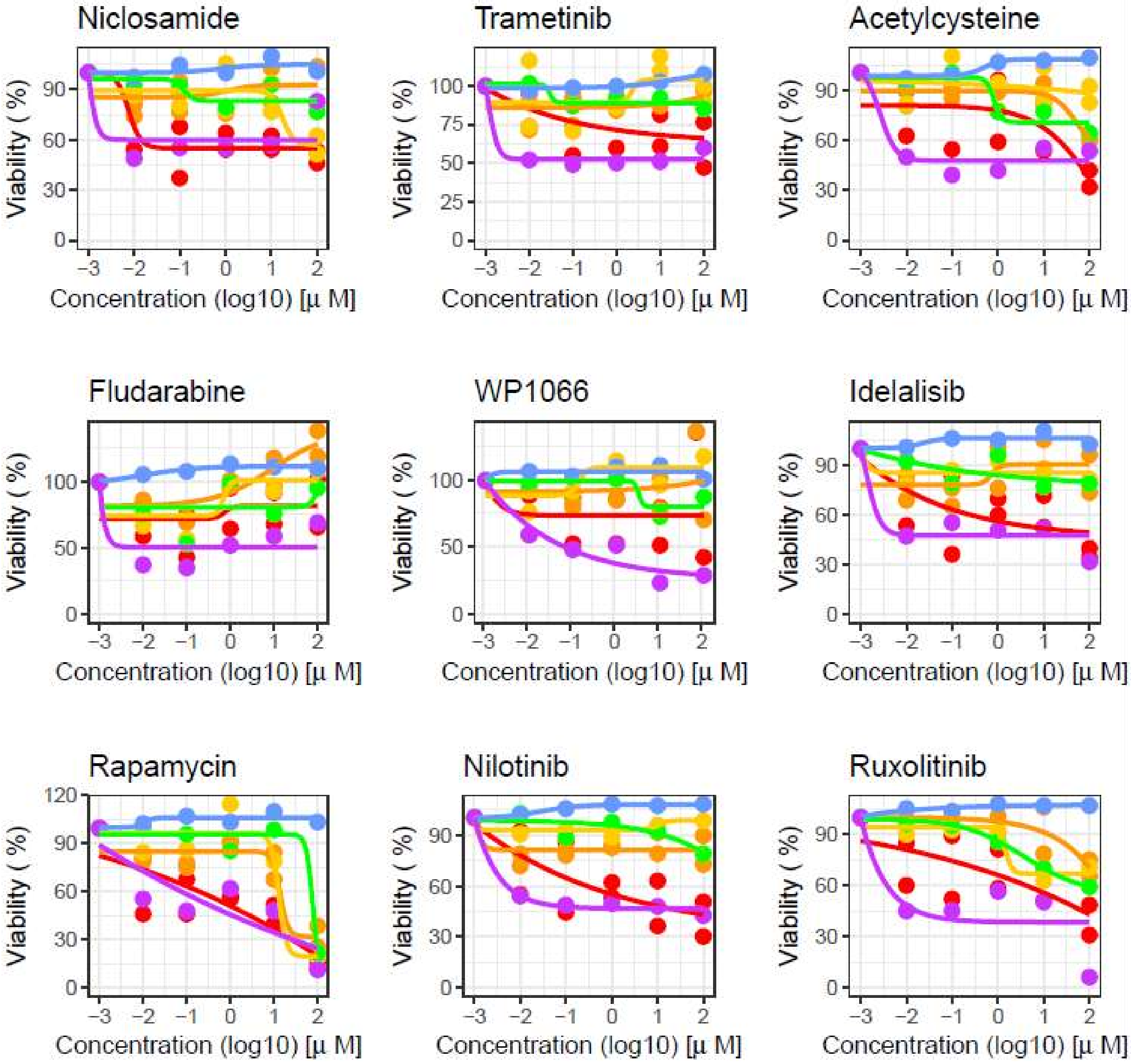
Niclosamide
Trametinib
Acetylcysteine
Fludarabine
WP1066
Idelalisib
Rapamycin
Nilotinib
Ruxolitinib
Viability (%)
Concentration (log10) [μ M]

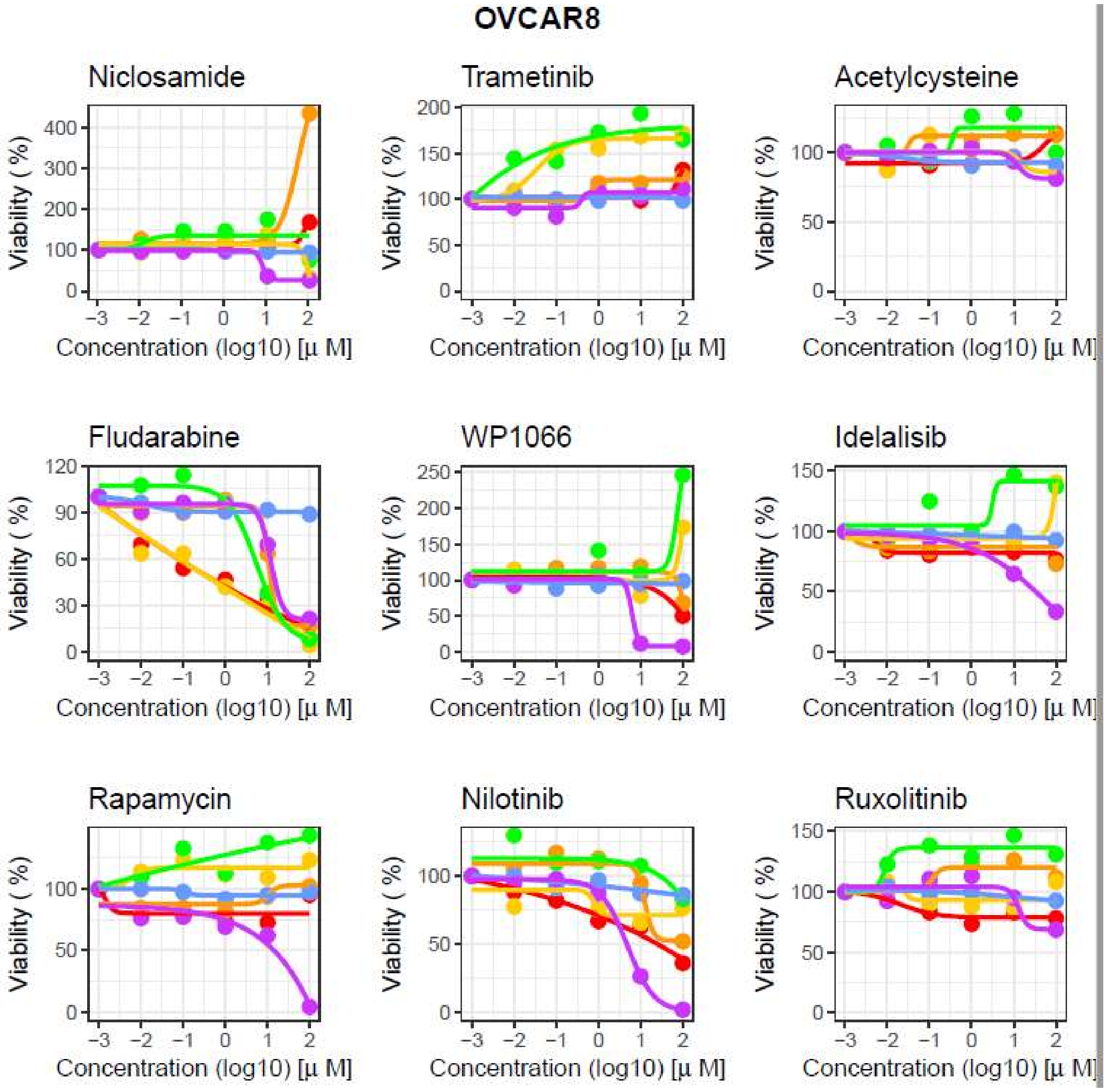
OVCAR8
Niclosamide
Trametinib
Acetylcysteine
Fludarabine
WP1066
Idelalisib
Rapamycin
Nilotinib
Ruxolitinib
Viability (%)
Concentration (log10) [µ M]

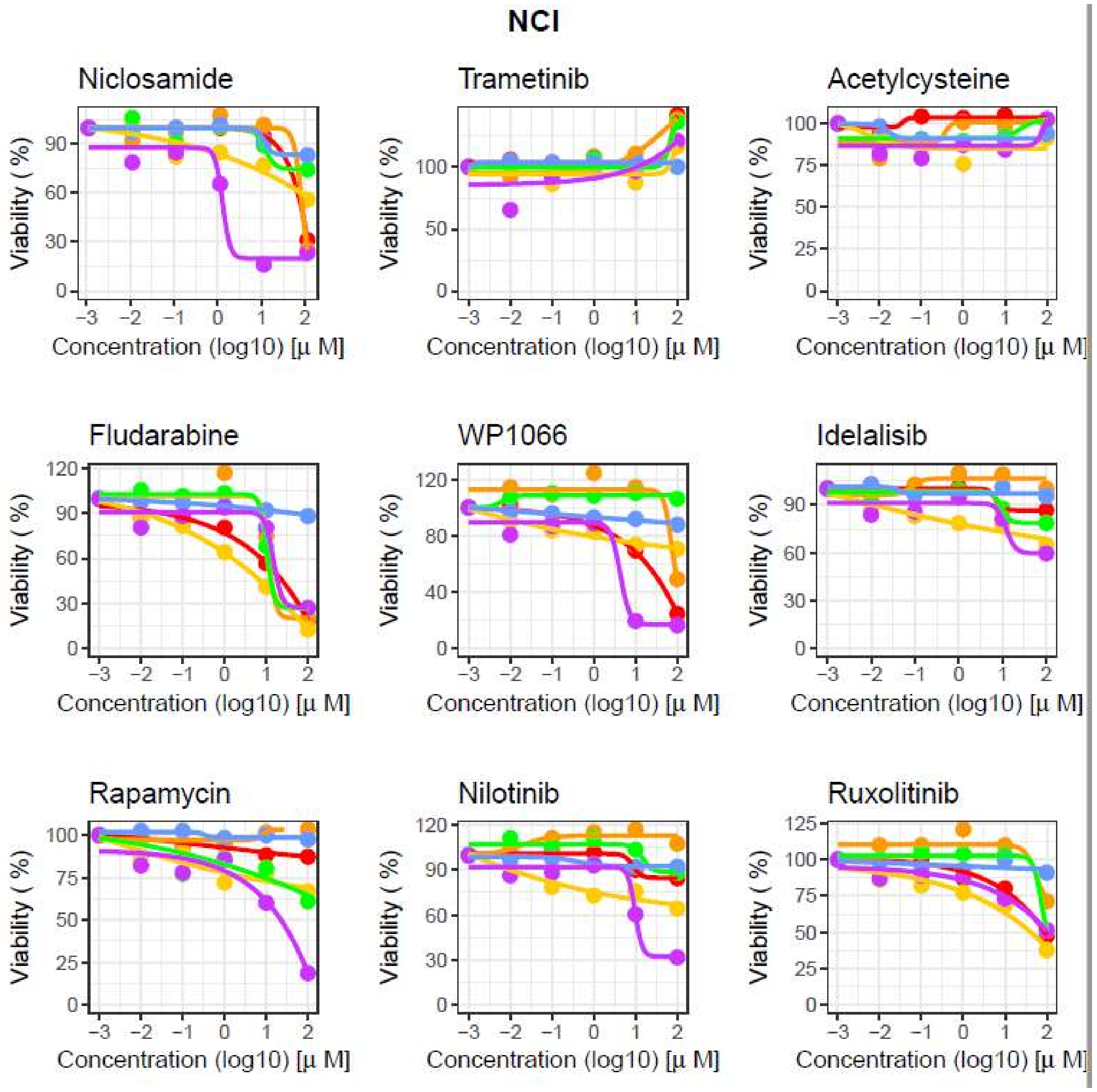

Supplementary Figure 7. Combination of novel therapeutics with paclitaxel at its IC20 (X-axes depict the log10 of the concentration whilst Y-axes is the relative viability) after incubation with five different patient-derived ascites or media.

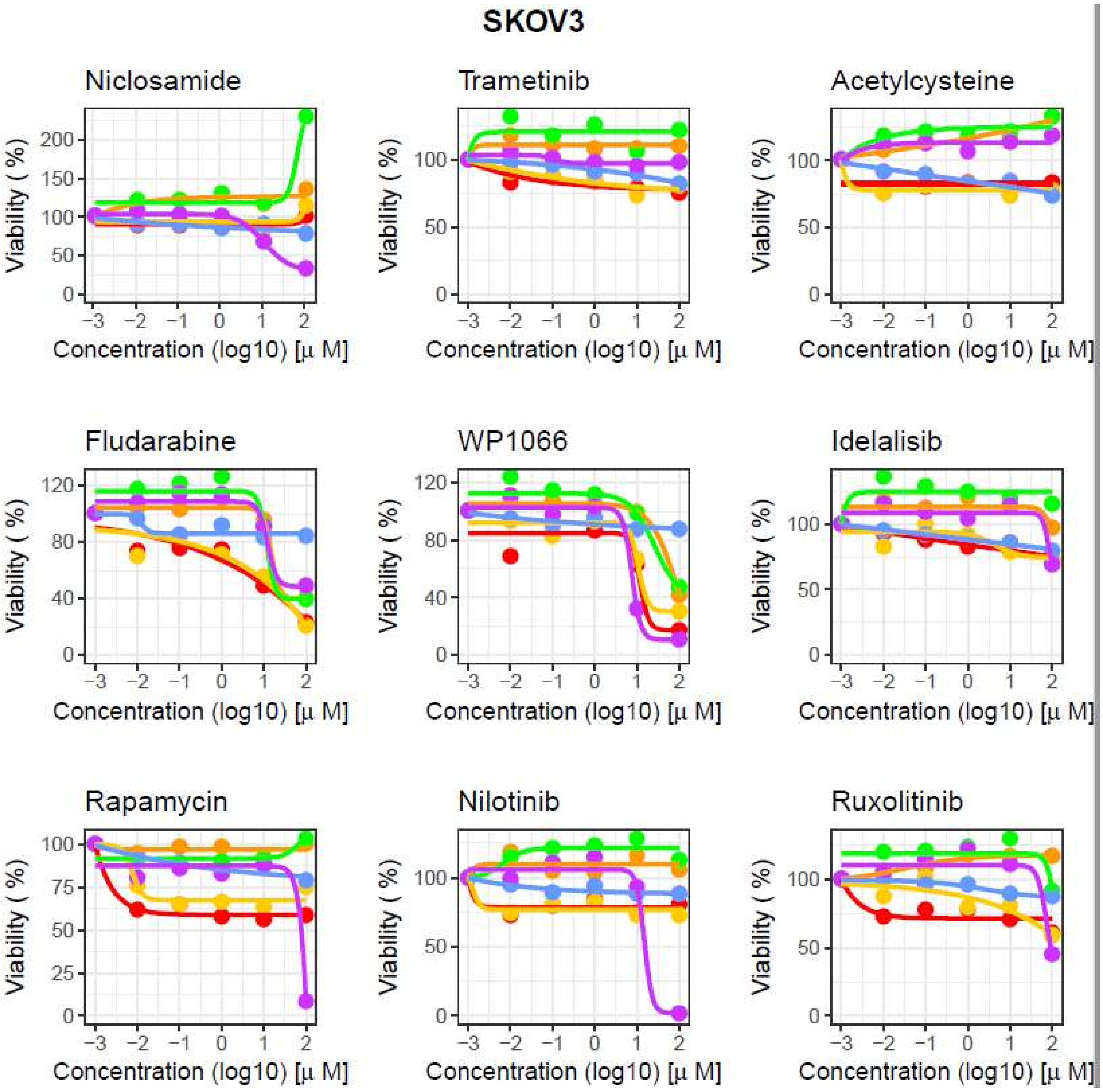
SKOV3
Niclosamide
Trametinib
Acetylcysteine
Fludarabine
WP1066
Idelalisib
Rapamycin
Nilotinib
Ruxolitinib
Viability (%)
Concentration (log10) [μ M]

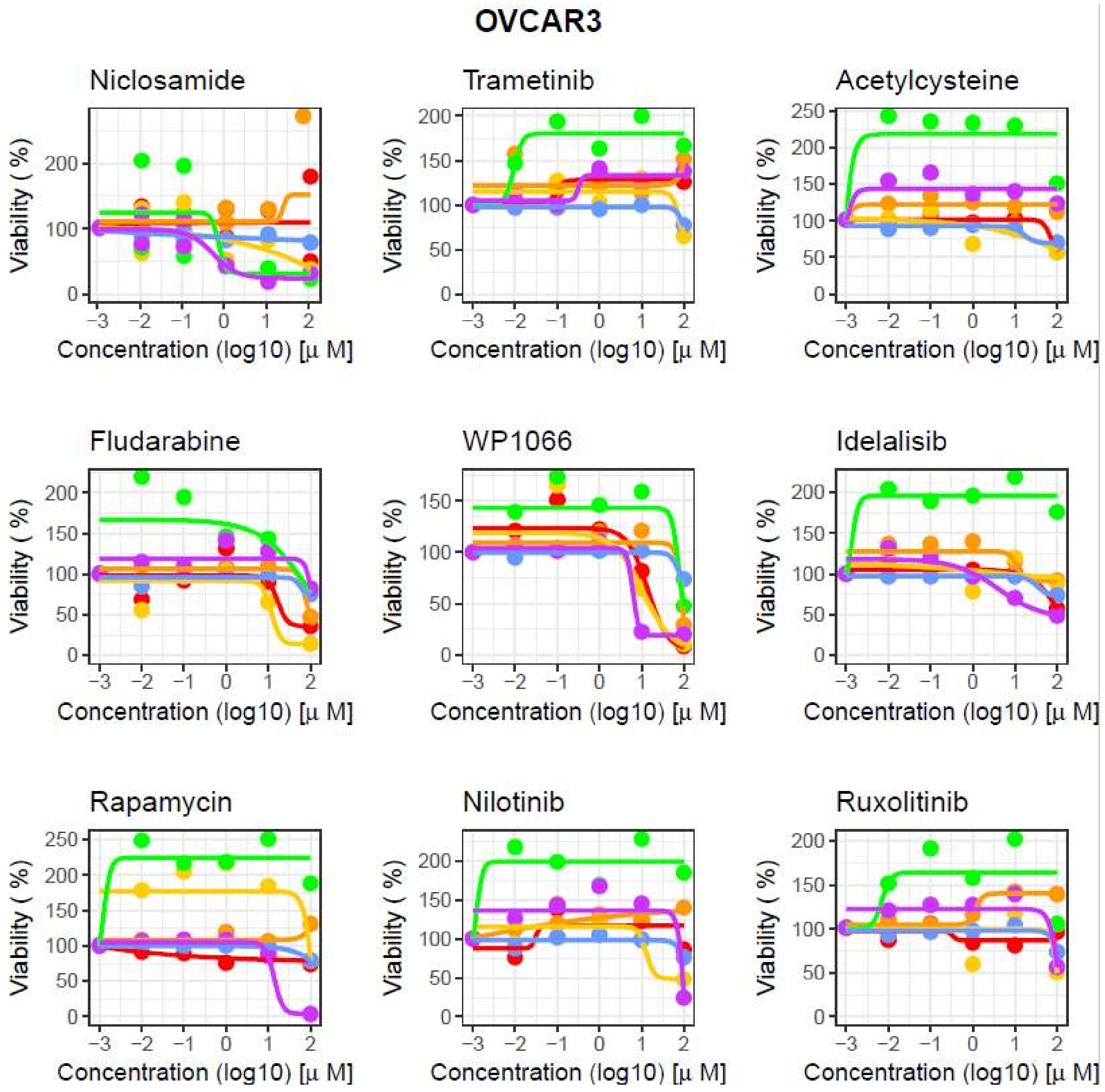
OVCAR3
Niclosamide
Trametinib
Acetylcysteine
Fludarabine
WP1066
Idelalisib
Rapamycin
Nilotinib
Ruxolitinib
Viability (%)
Concentration (log10) [μ M]

OVCAR5

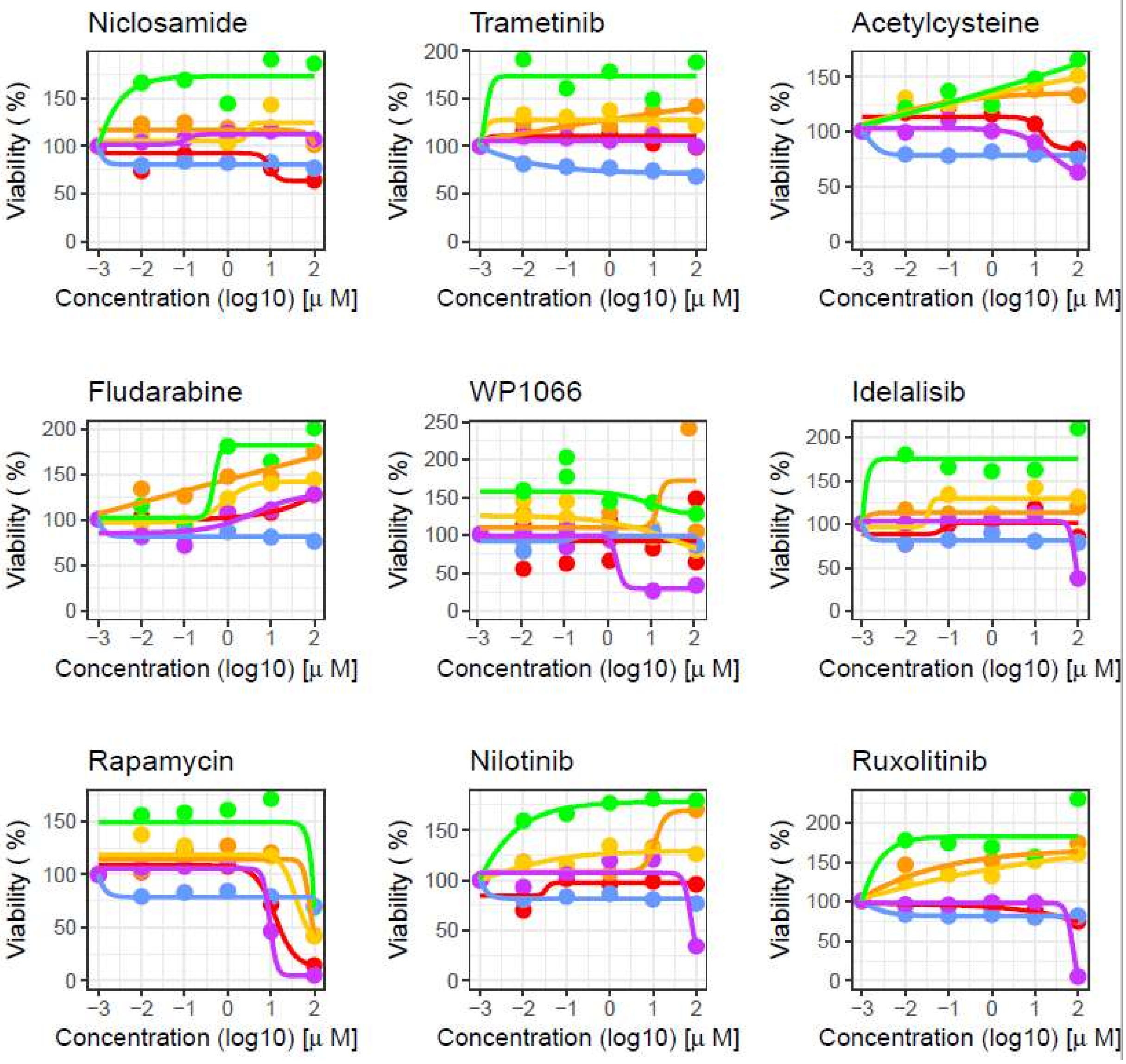

Niclosamide
Trametinib
Acetylcysteine
Fludarabine
WP1066
Idelalisib
Rapamycin
Nilotinib
Ruxolitinib
Viability (%)
Concentration (log10) [μ M]

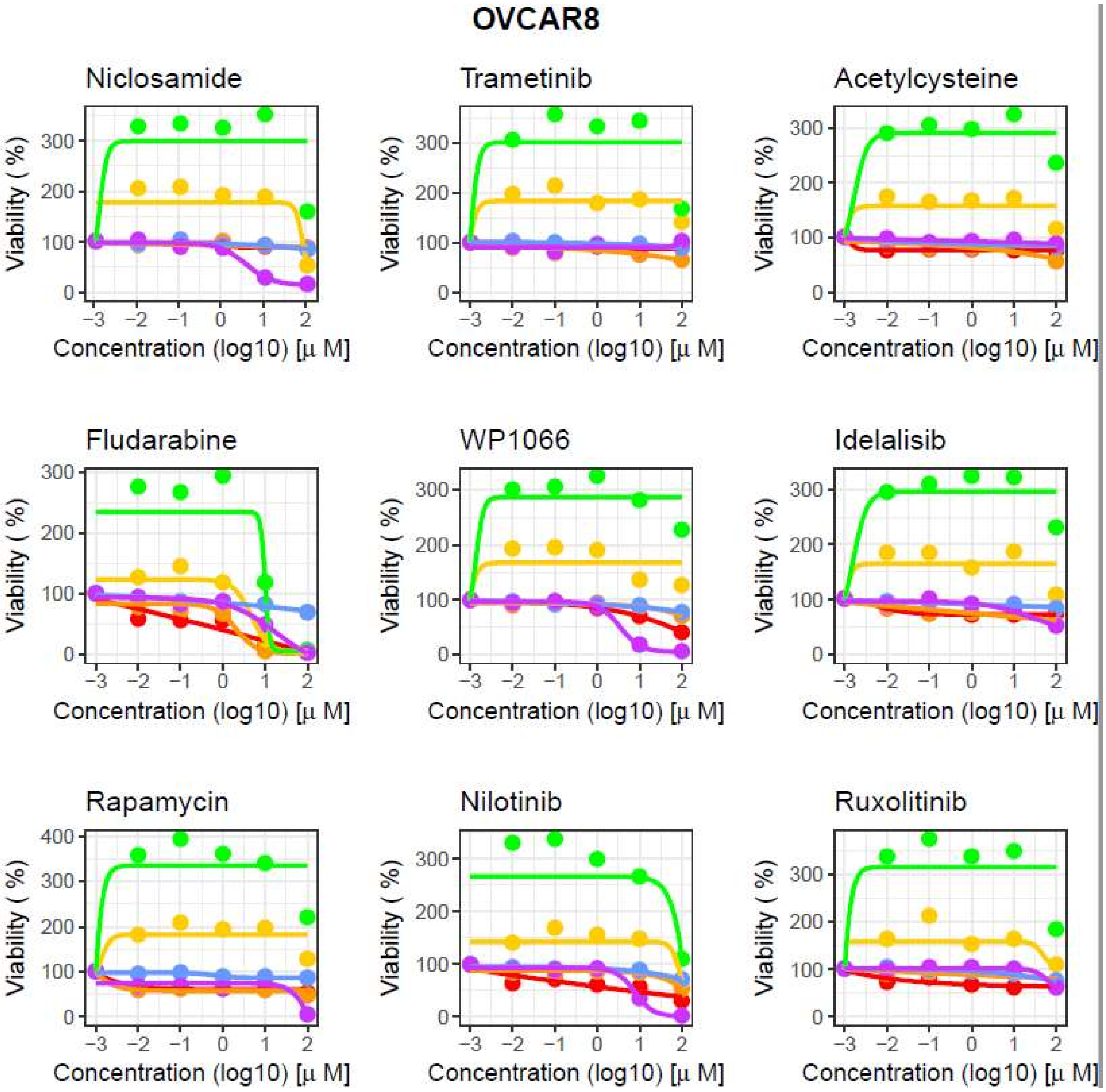
OVCAR8
Niclosamide
Trametinib
Acetylcysteine
Fludarabine
WP1066
Idelalisib
Rapamycin
Nilotinib
Ruxolitinib
Viability (%)
Concentration (log10) [μ M]

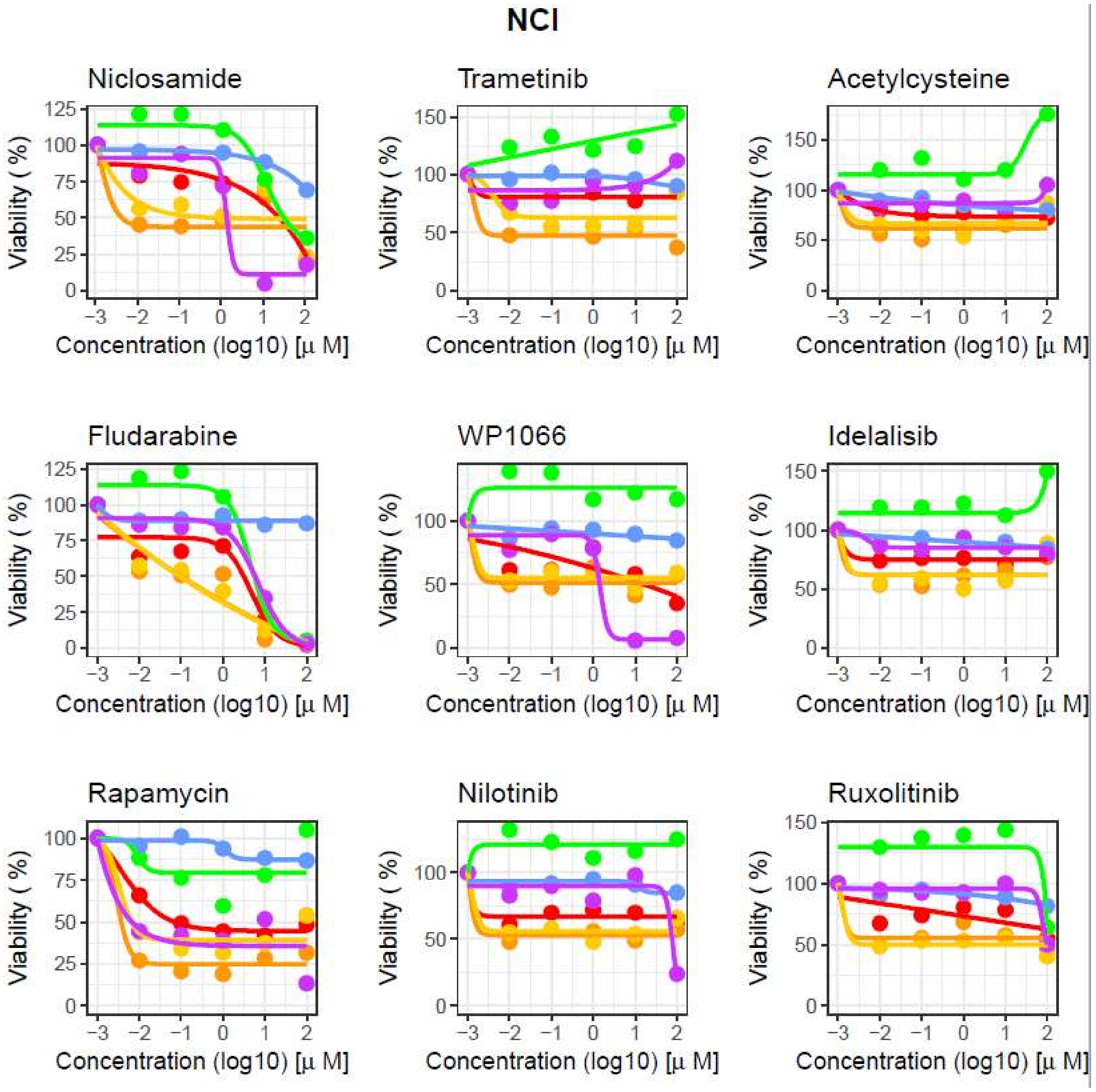

Supplementary Figure 8. Combination of novel therapeutics with carboplatin at its IC20 (X-axes depict the log10 of the concentration whilst Y-axes is the relative viability) after incubation with patient-derived ascites (n=5) or media.

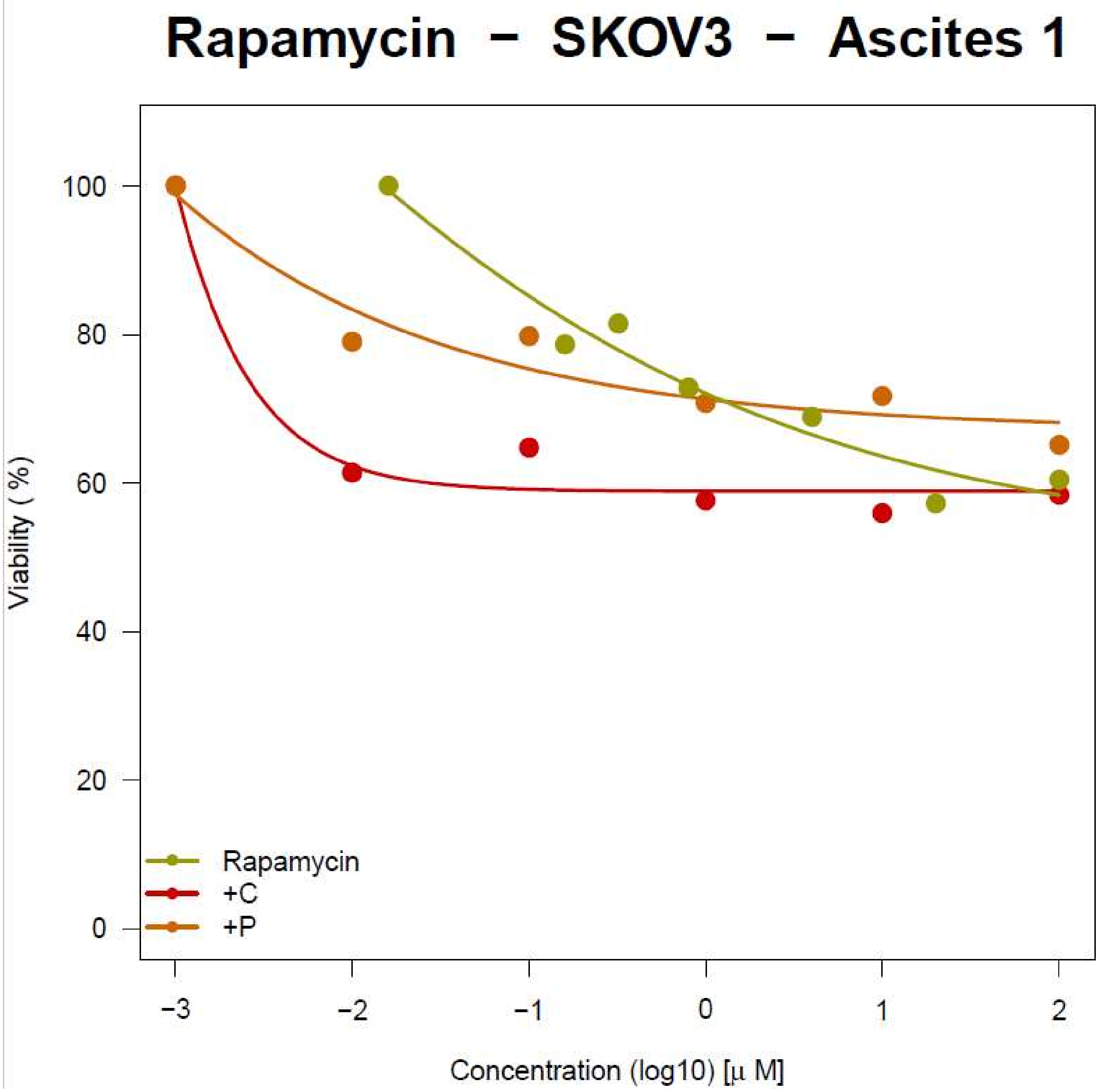

Rapamycin − SKOV3 − Ascites 1
Viability (%)
100
80
60
40
20
0
−3
−2
−1
0
1
2
Concentration (log10) [µ M]
Rapamycin
+C
+P

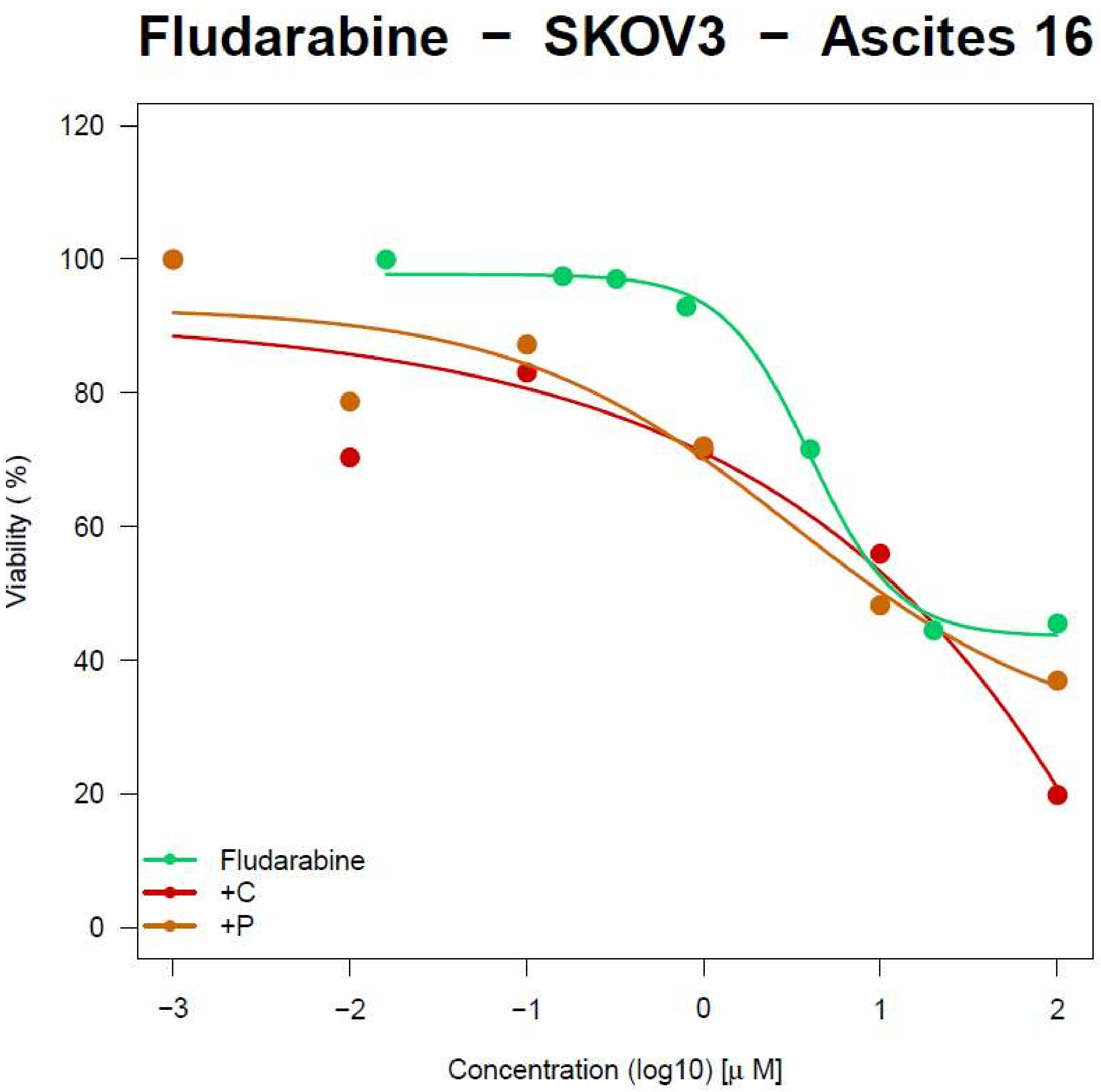
Fludarabine − SKOV3 − Ascites 16
120
100
80
60
40
20
0
Viability (%)
−3
−2
−1
0
1
2
Concentration (log10) [µ M]
Fludarabine
+C
+P

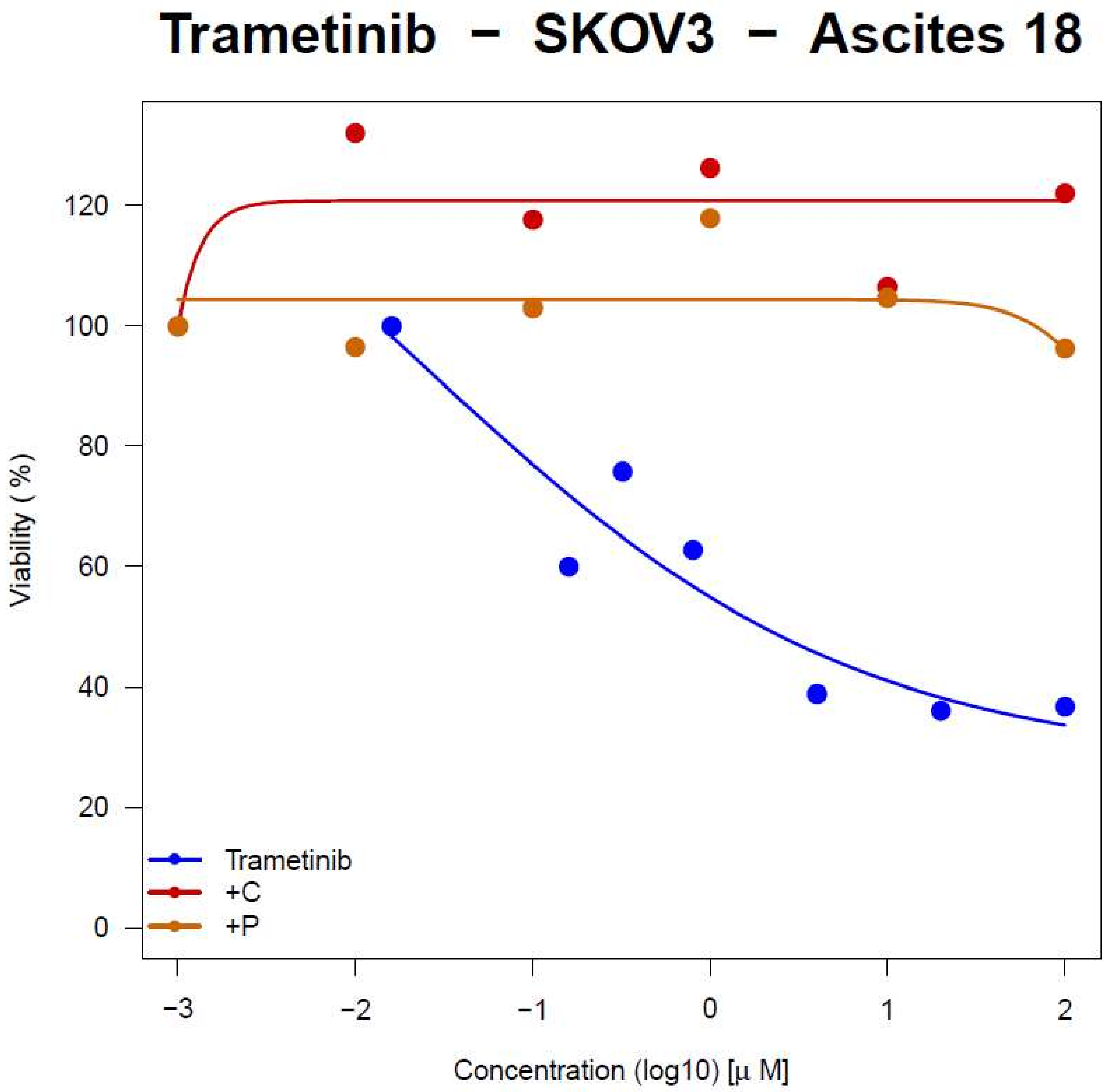
Trametinib – SKOV3 – Ascites 18
Viability (%)
0
20
40
60
80
100
120
−3
−2
−1
0
1
2
Concentration (log10) [µ M]
Trametinib
+C
+P

Acetylcysteine – SKOV3 – Ascites 19

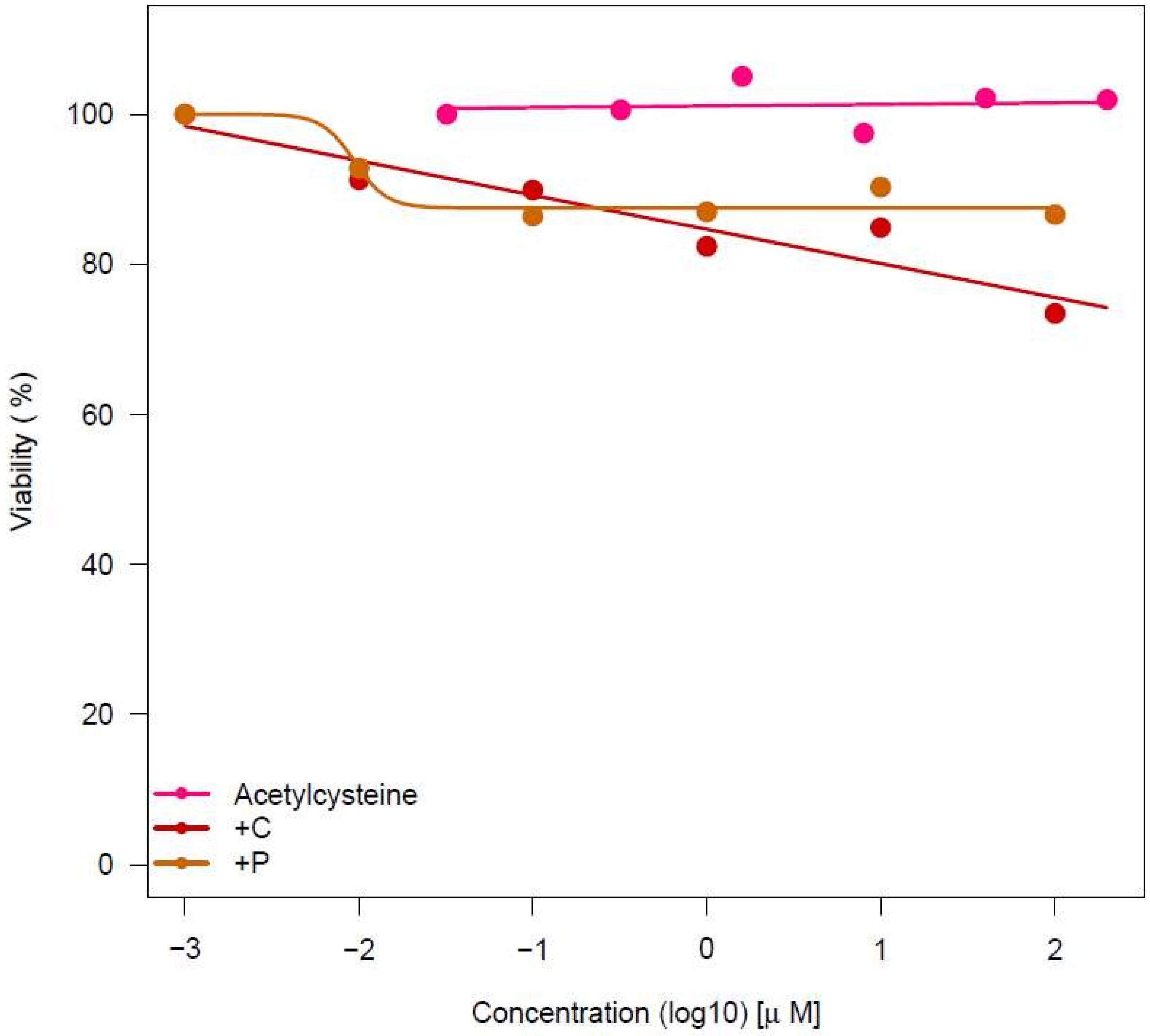

Niclosamide − OVCAR3 − Ascites 19

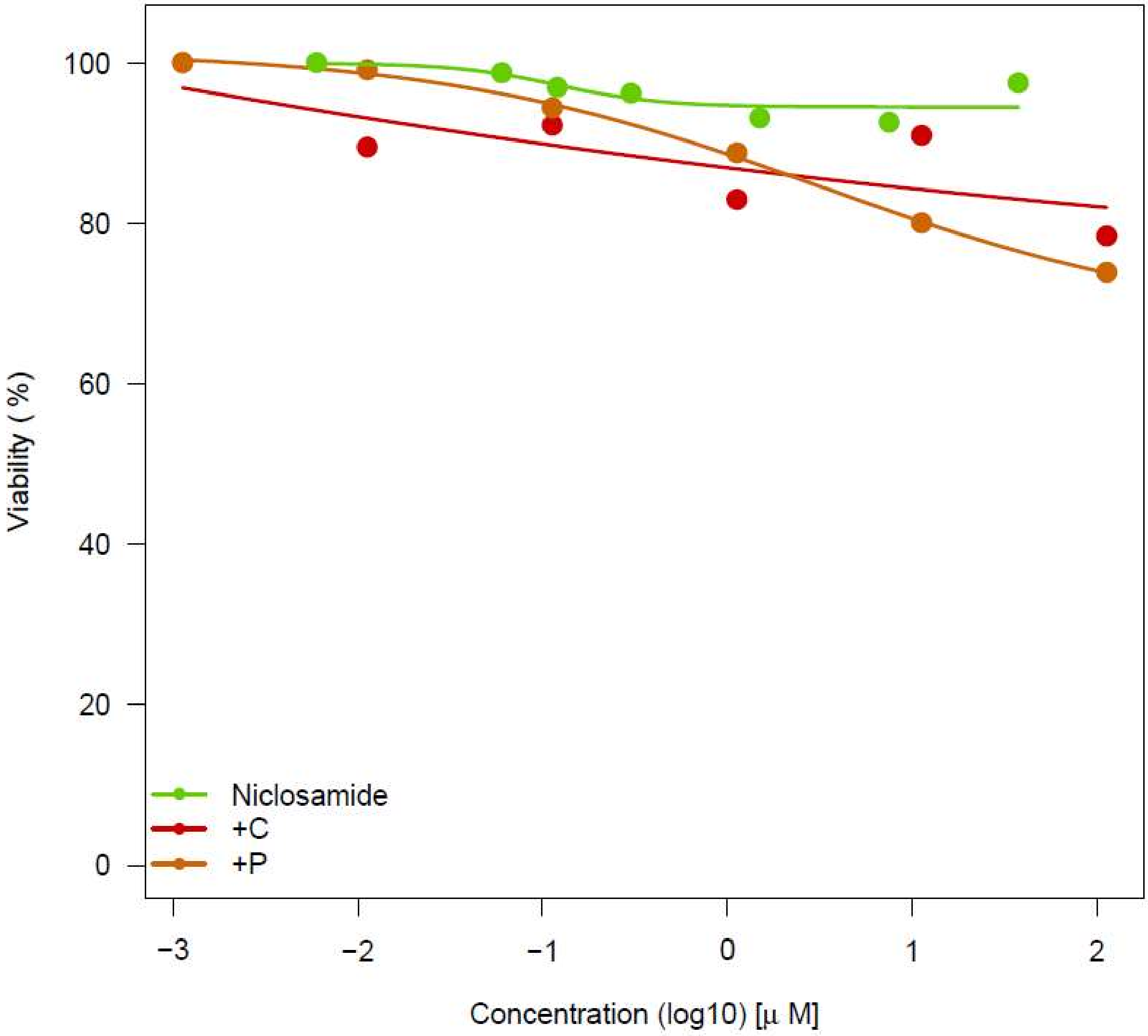

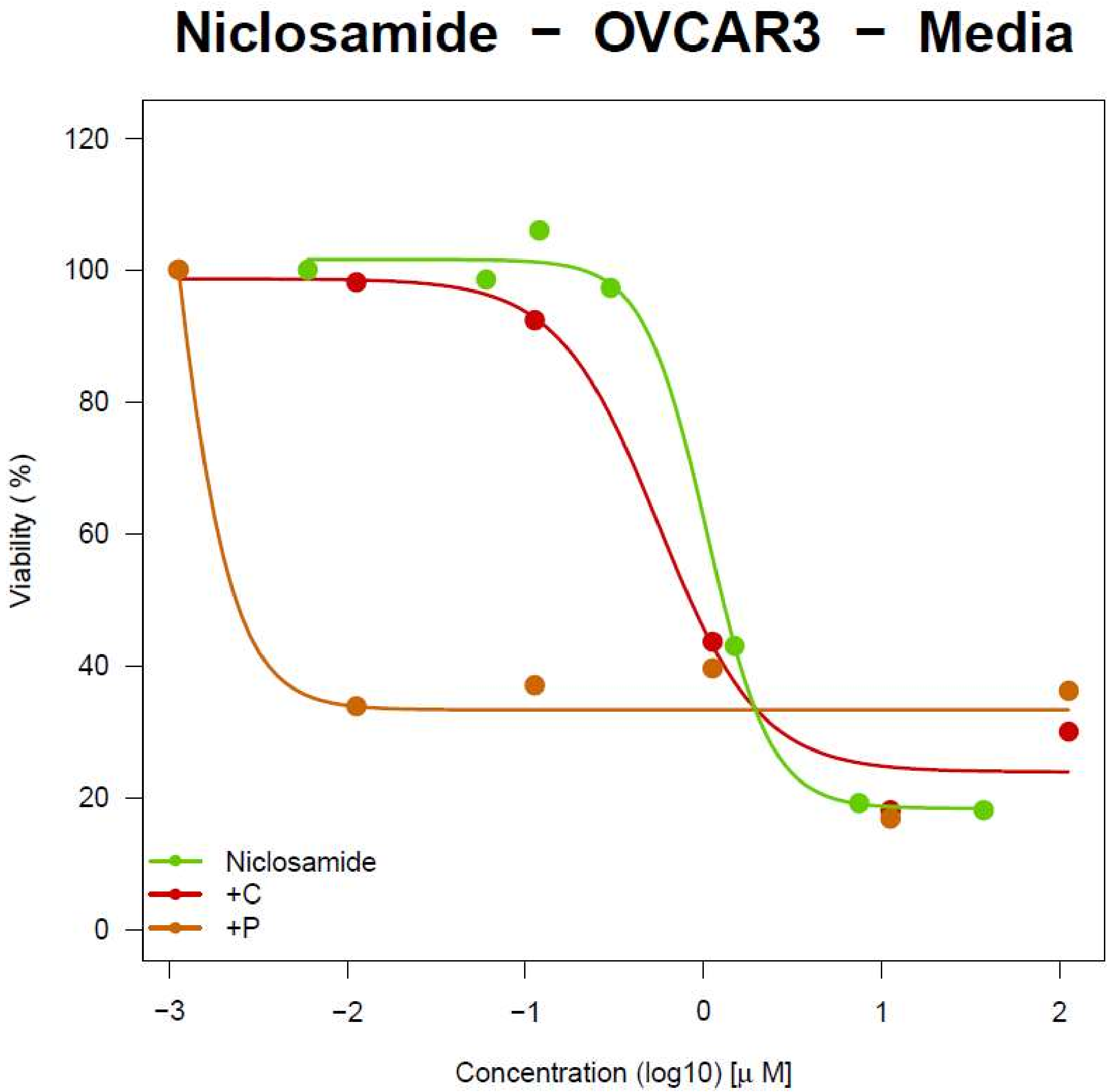
Niclosamide − OVCAR3 − Media
120
100
80
60
40
20
0
Viability (%)
−3
−2
−1
0
1
2
Concentration (log10) [μ M]
Niclosamide
+C
+P

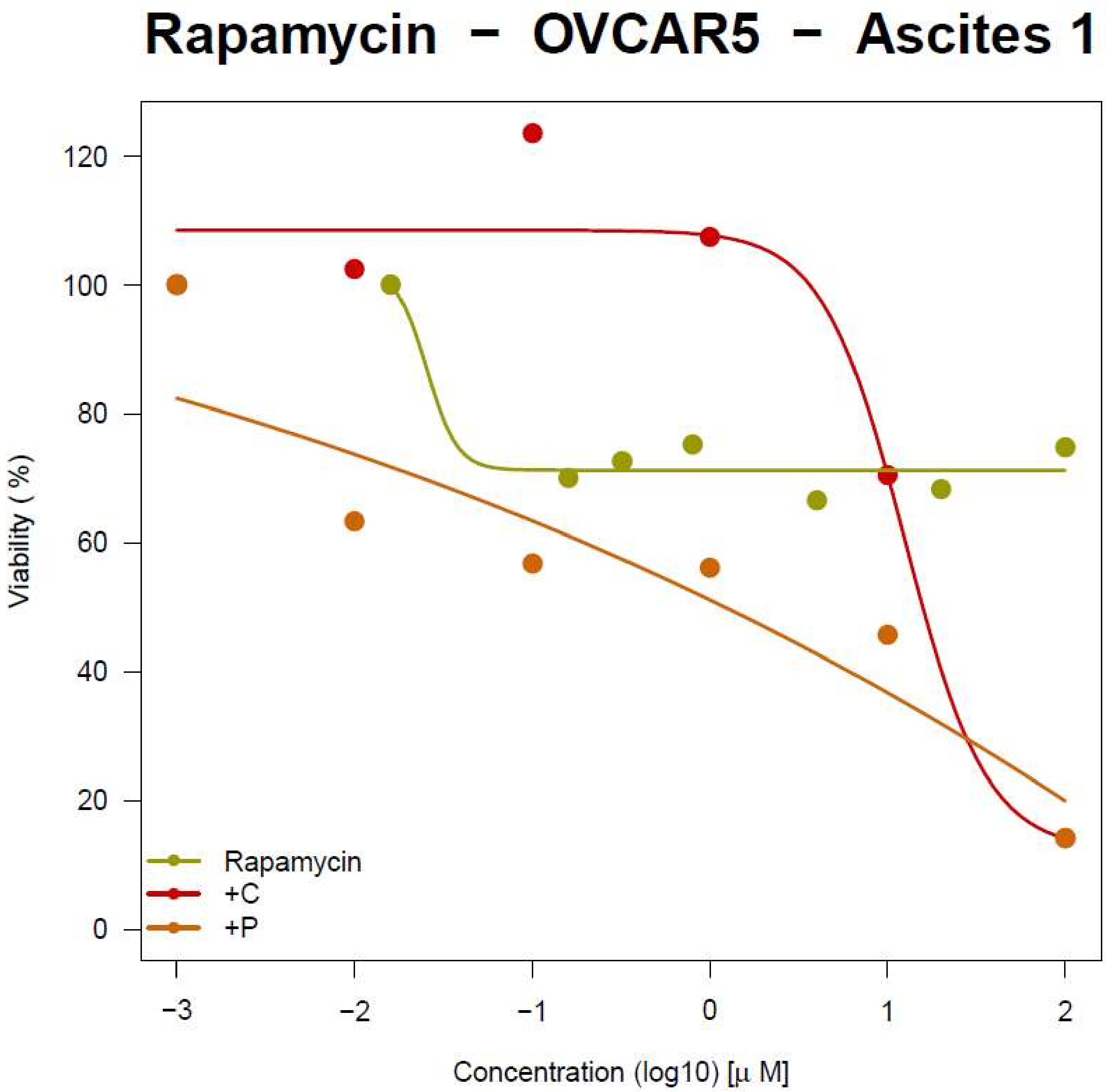
Rapamycin − OVCAR5 − Ascites 1
Viability (%)
Concentration (log10) [μ M]
Rapamycin
+C
+P
120
100
80
60
40
20
0
−3
−2
−1
0
1
2

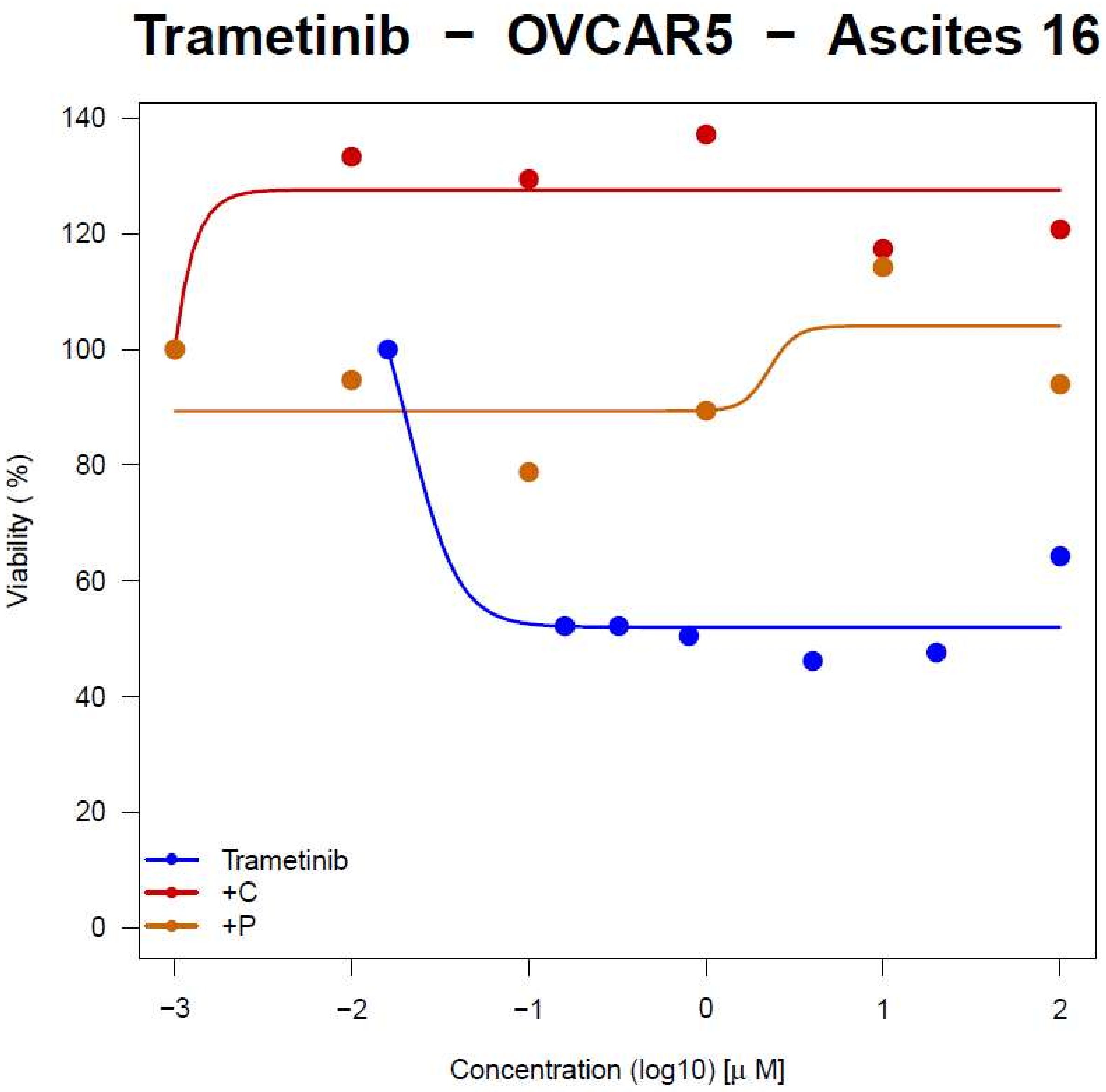
Trametinib − OVCAR5 − Ascites 16
Viability (%)
Concentration (log10) [μ M]
Trametinib
+C
+P
140
120
100
80
60
40
20
0
−3
−2
−1
0
1
2

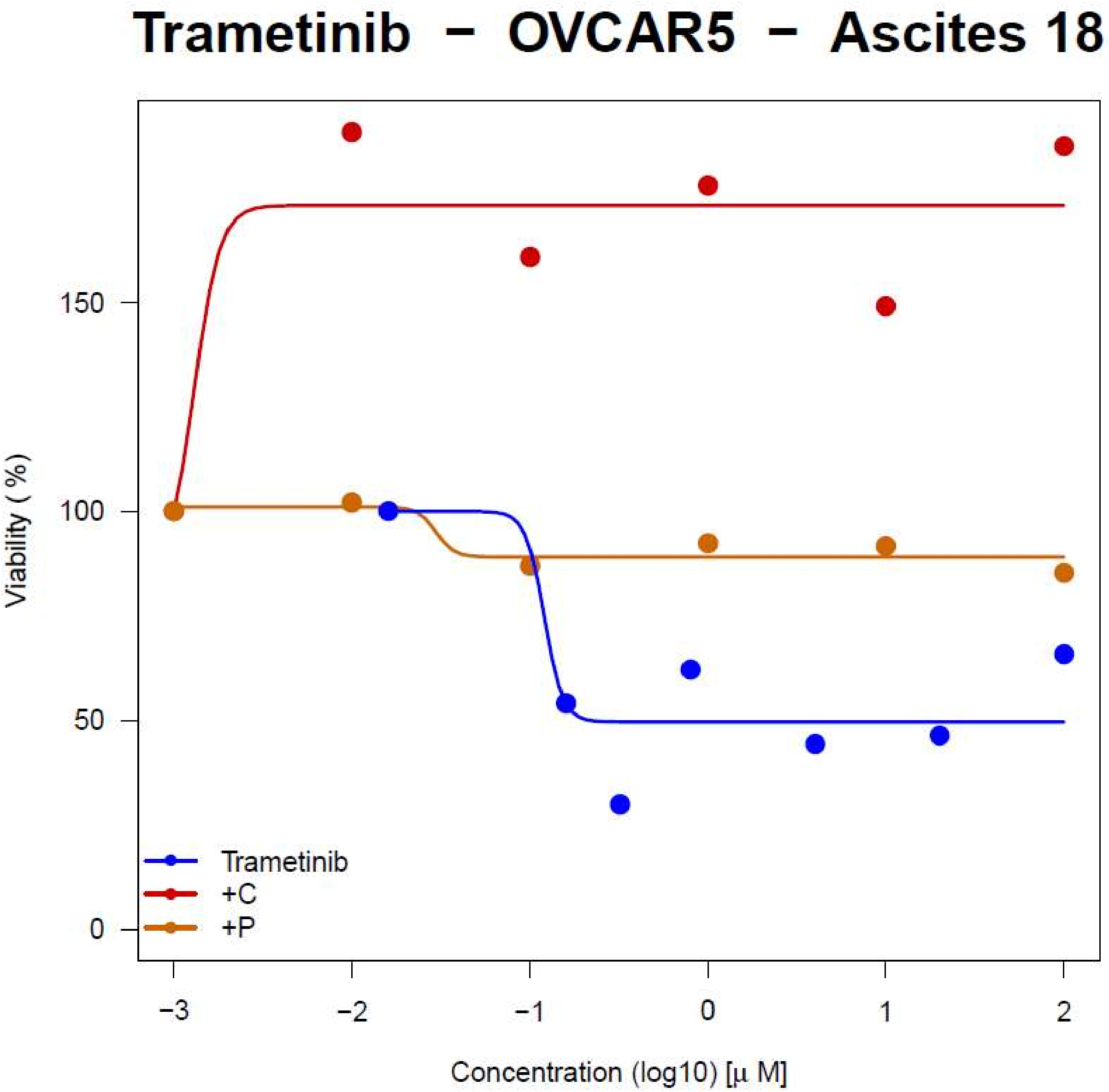
Trametinib − OVCAR5 − Ascites 18
Viability (%)
0
50
100
150
−3
−2
−1
0
1
2
Concentration (log10) [μ M]
Trametinib
+C
+P

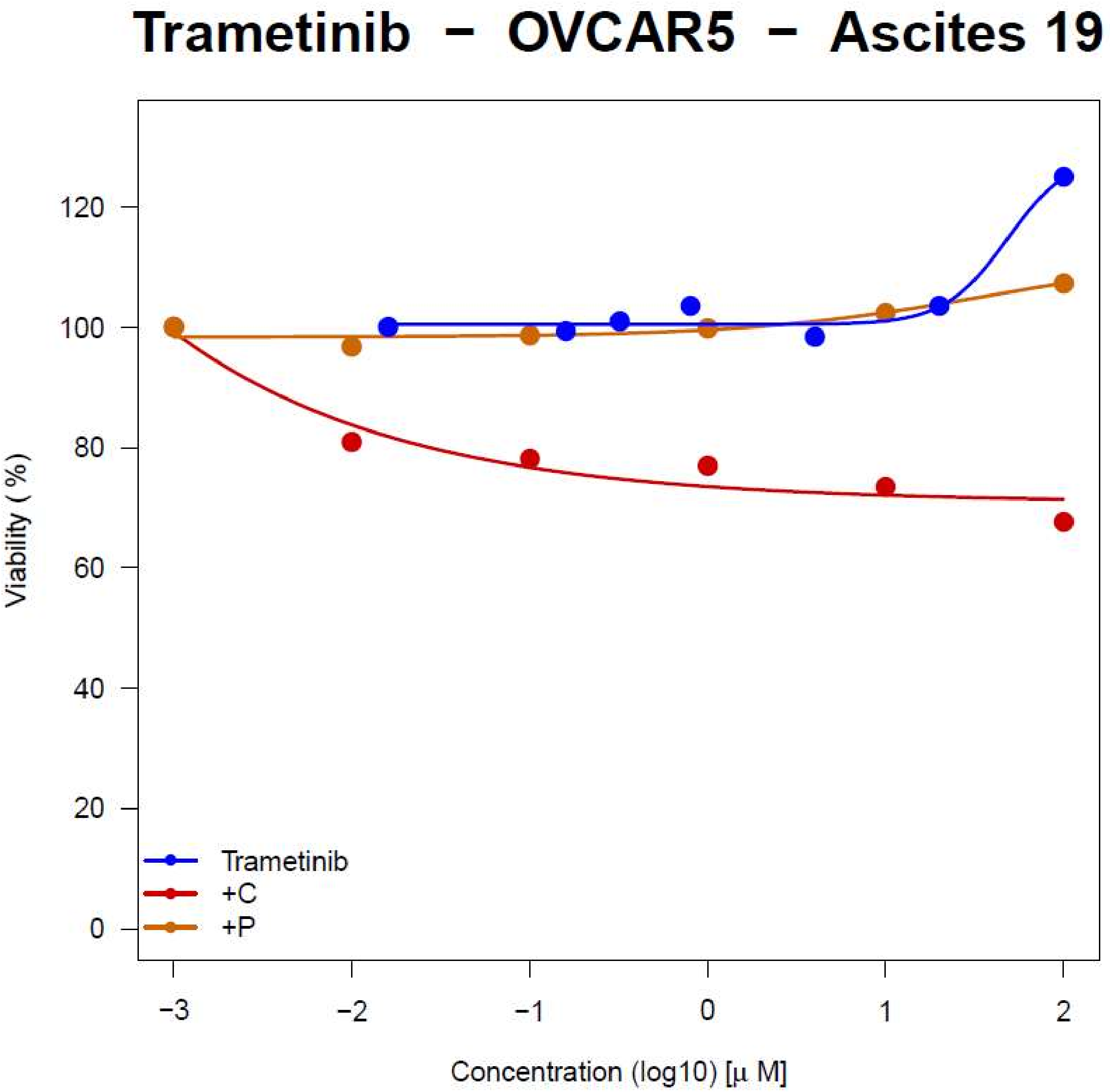
Trametinib − OVCAR5 − Ascites 19
Viability (%)
0
20
40
60
80
100
120
−3
−2
−1
0
1
2
Concentration (log10) [μ M]
Trametinib
+C
+P

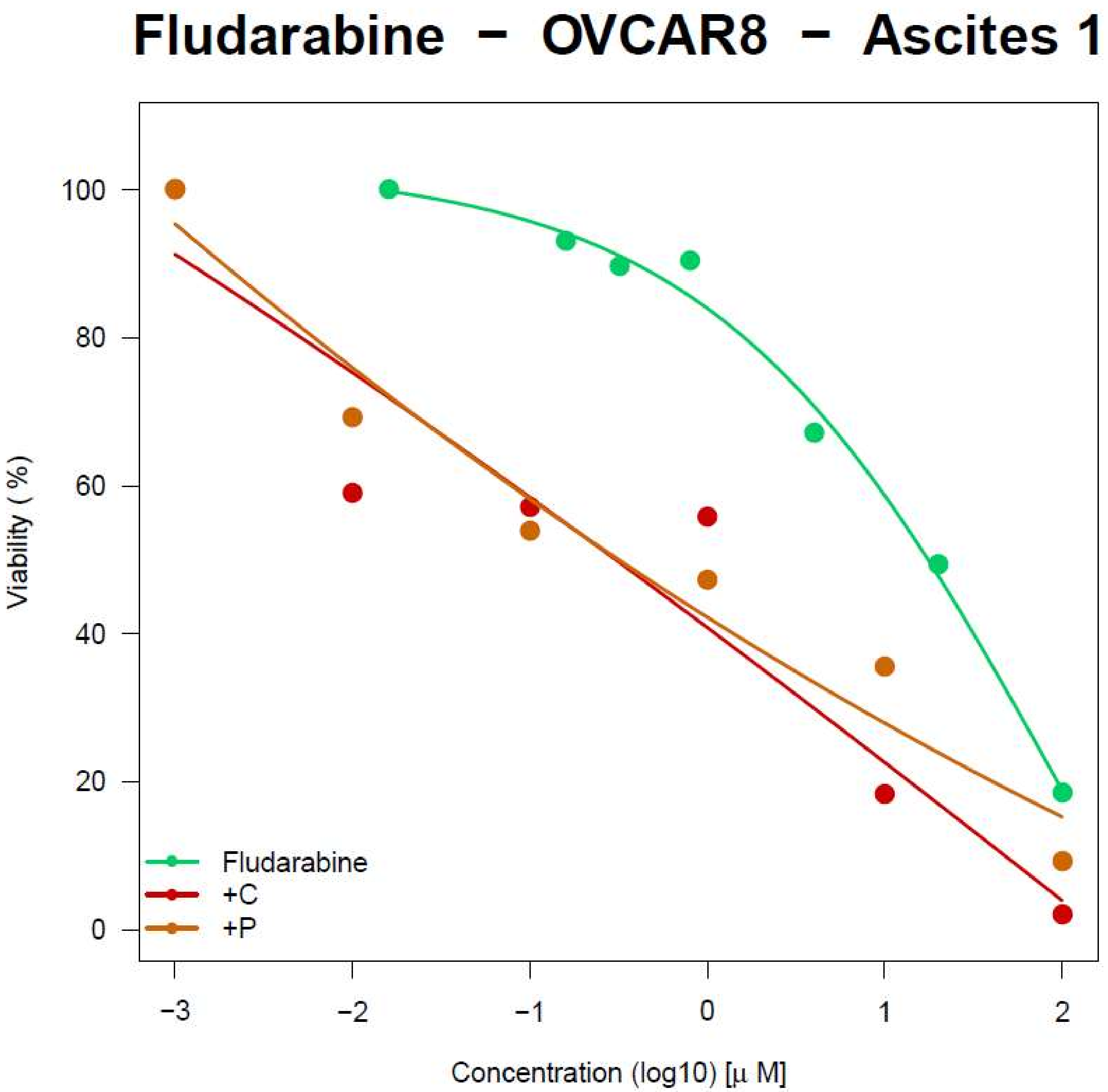

Fludarabine − OVCAR8 − Ascites 1
Viability (%)
100
80
60
40
20
0
−3
−2
−1
0
1
2
Concentration (log10) [µ M]
Fludarabine
+C
+P

Fludarabine − OVCAR8 − Ascites 9

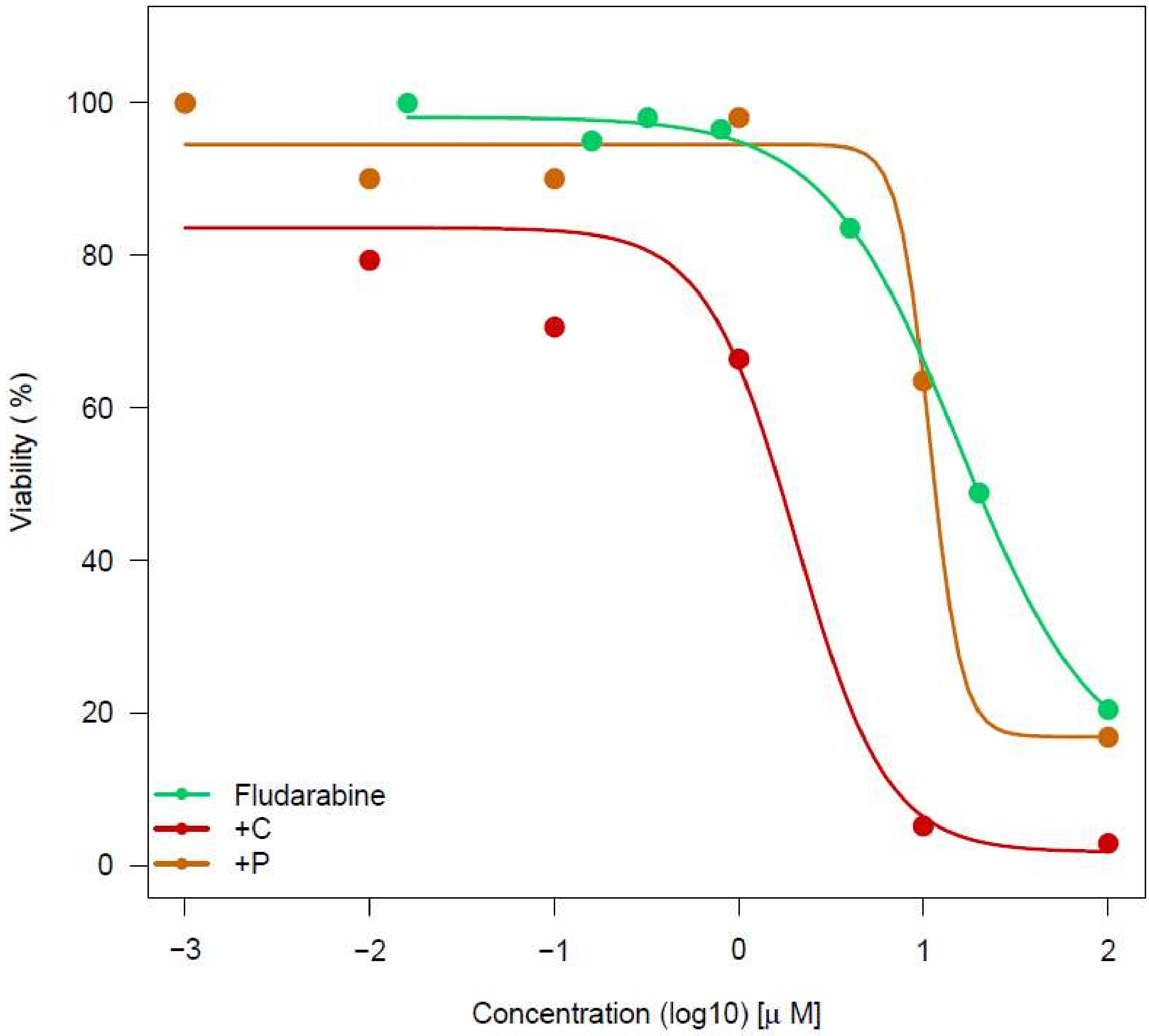

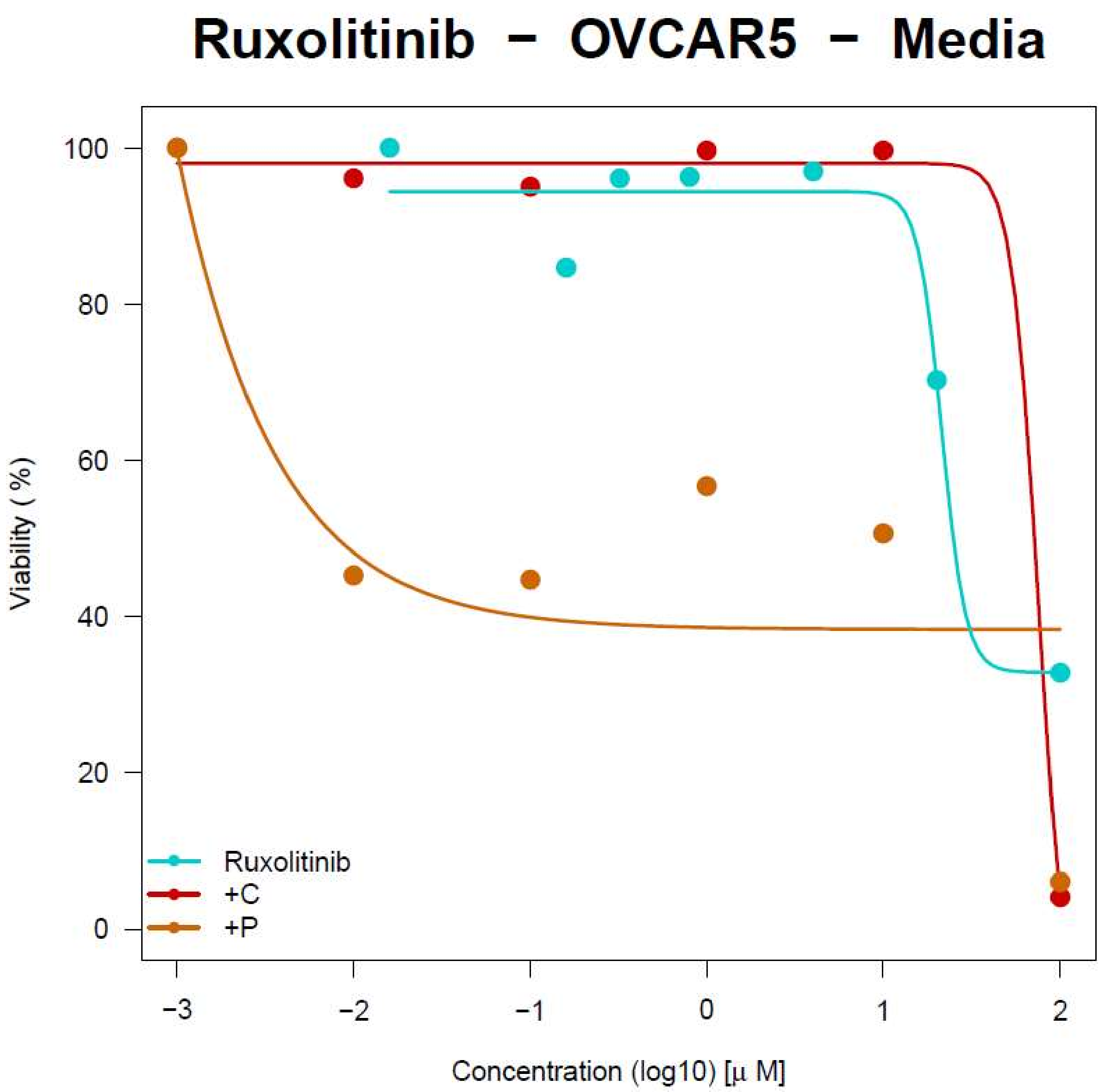

Ruxolitinib − OVCAR5 − Media
Viability (%)
Concentration (log10) [μ M]
0
20
40
60
80
100
−3
−2
−1
0
1
2
Ruxolitinib
+C
+P

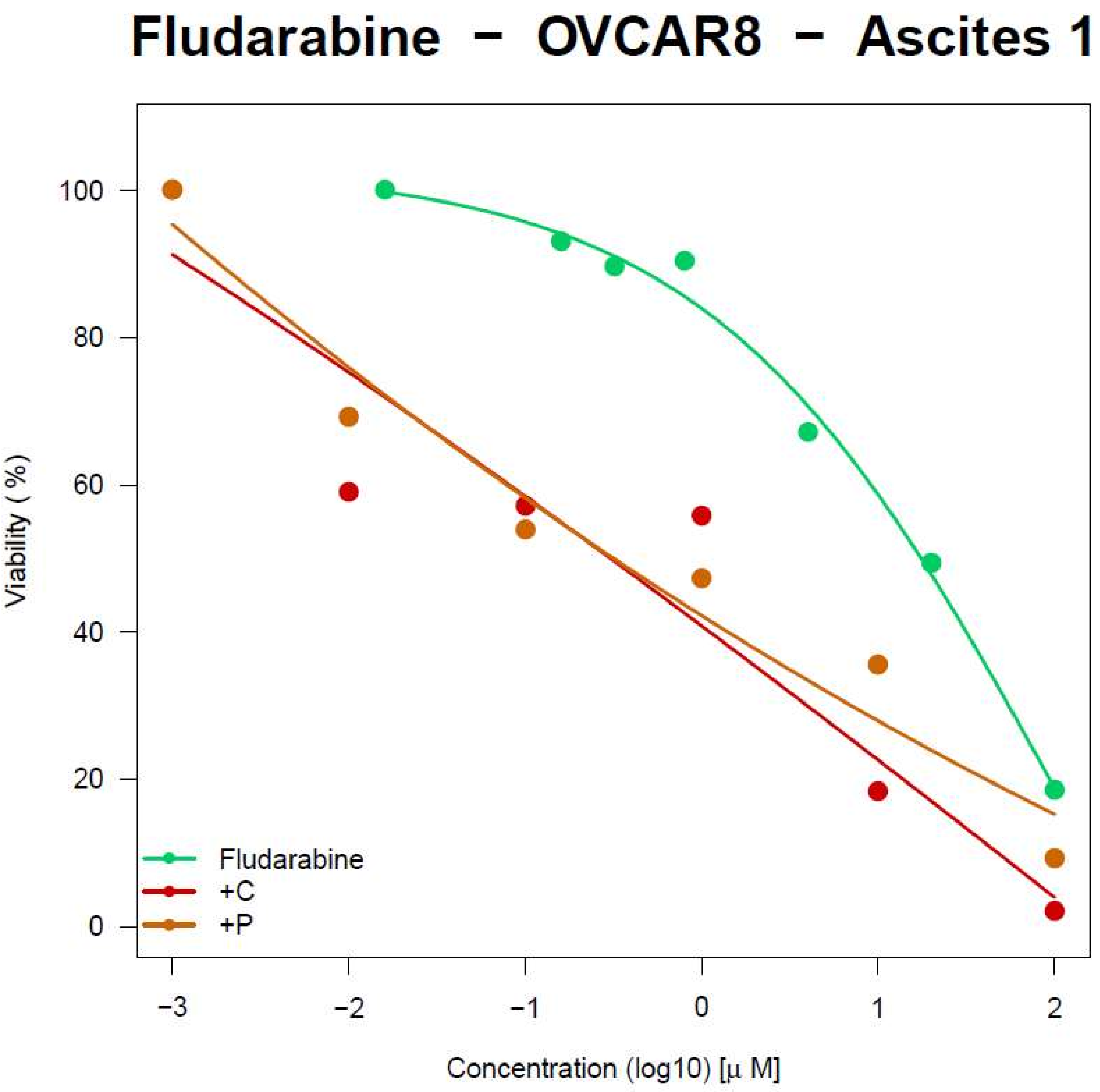
Fludarabine − OVCAR8 − Ascites 1
Viability (%)
0
20
40
60
80
100
−3
−2
−1
0
1
2
Concentration (log10) [μ M]
Fludarabine
+C
+P

Fludarabine − OVCAR8 − Ascites 9

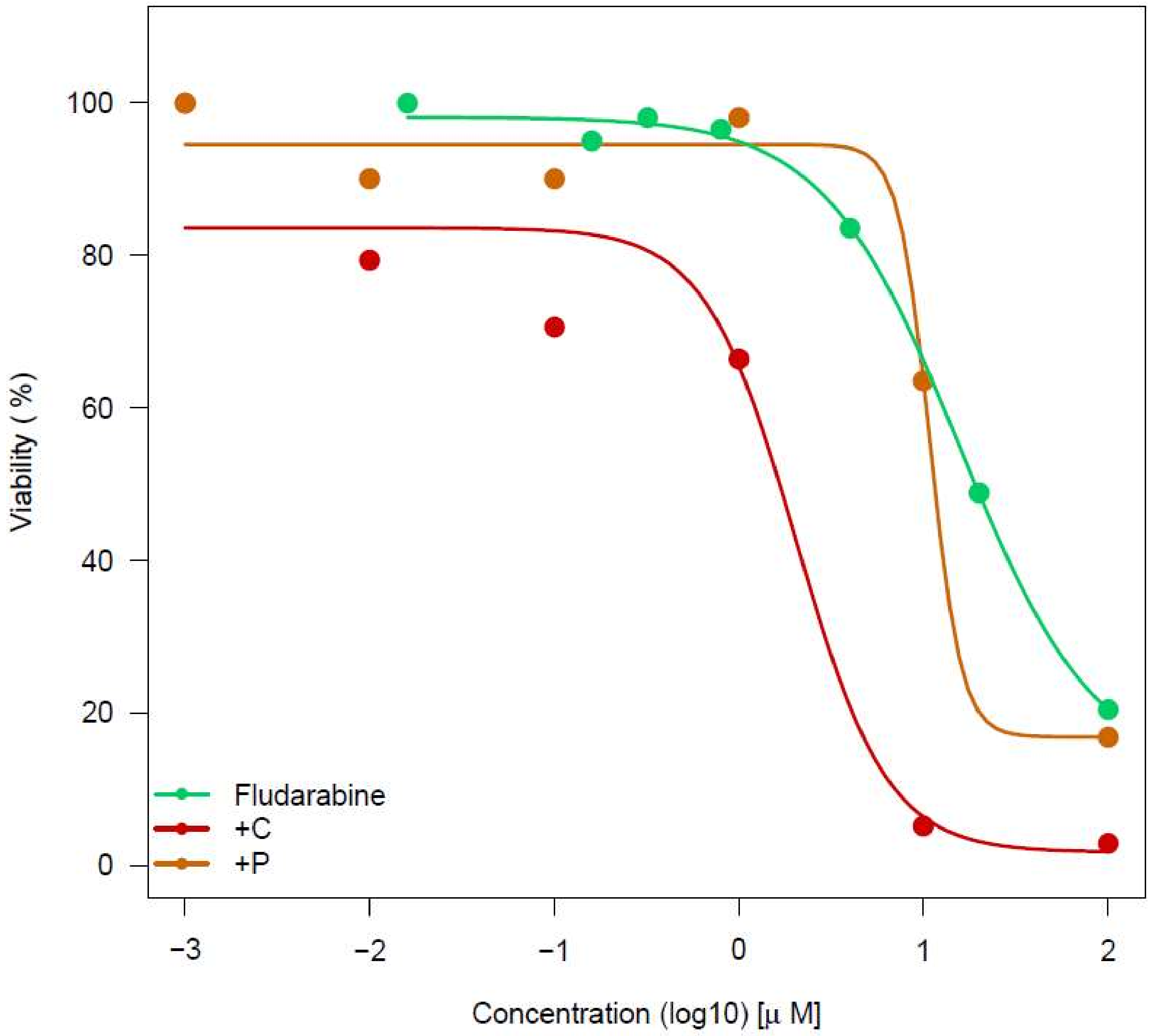

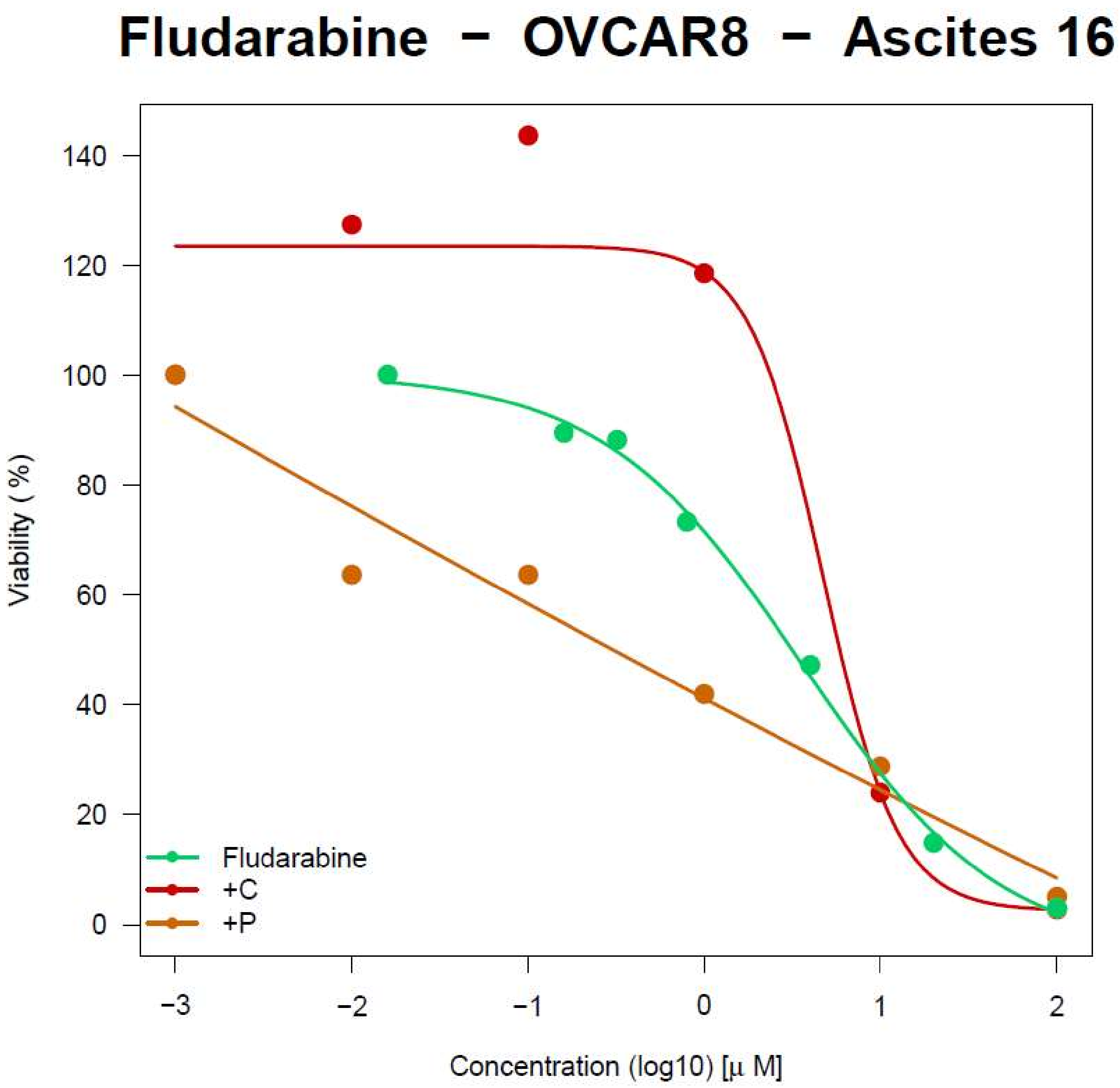
Fludarabine − OVCAR8 − Ascites 16
Viability (%)
140
120
100
80
60
40
20
0
−3
−2
−1
0
1
2
Concentration (log10) [μ M]
Fludarabine
+C
+P

Fludarabine − OVCAR8 − Ascites 19

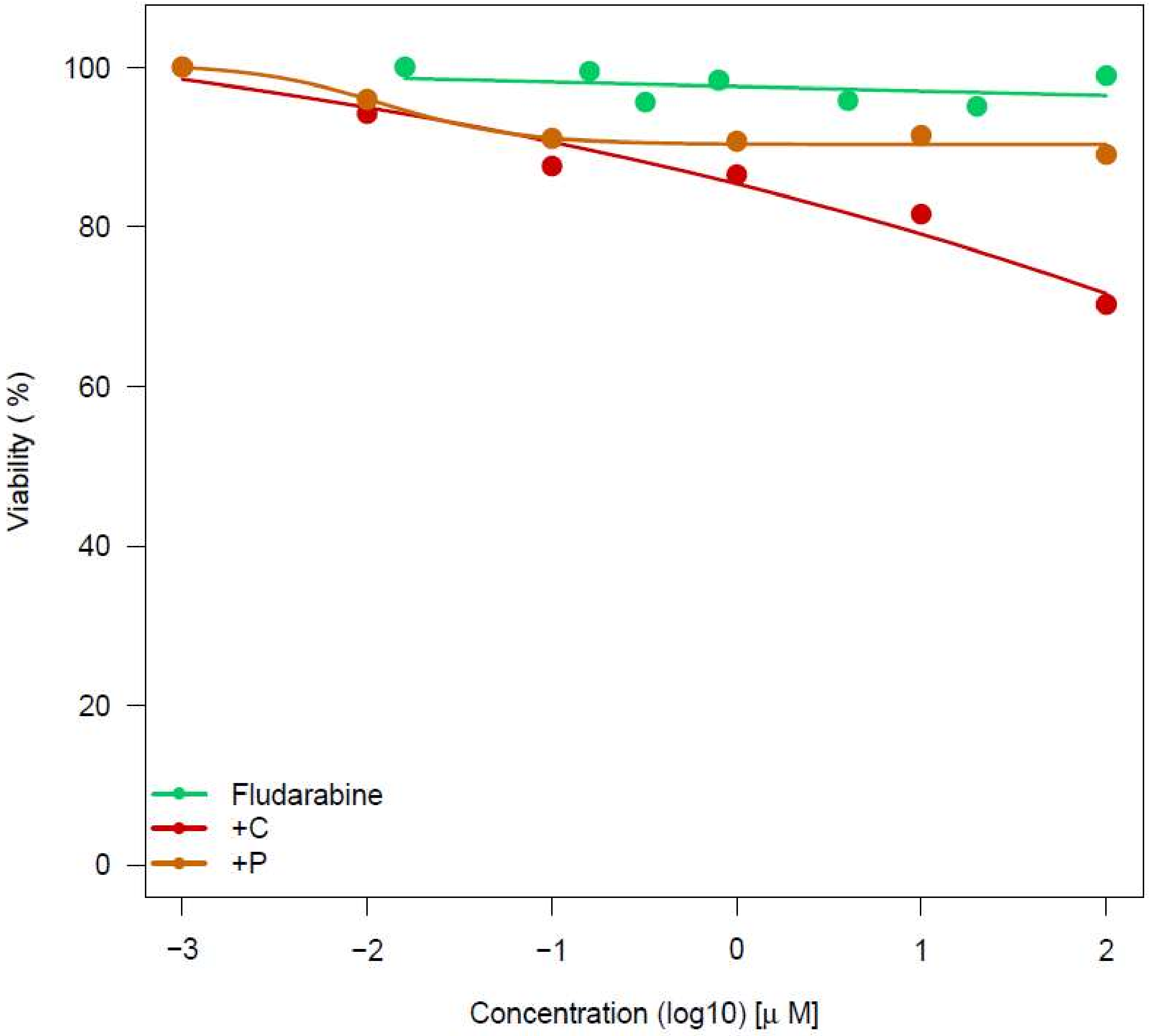

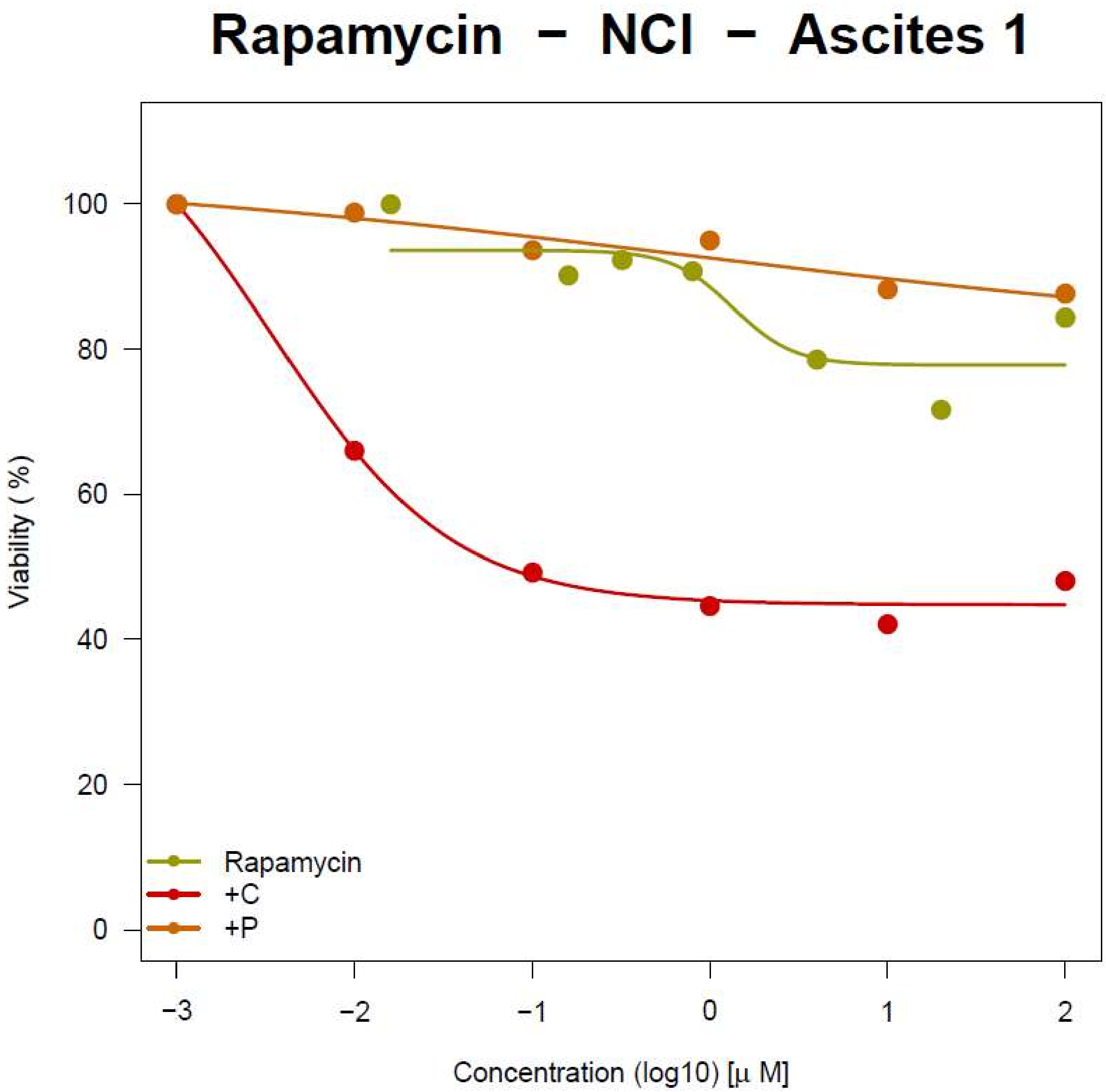

Rapamycin − NCI − Ascites 1
Viability (%)
100
80
60
40
20
0
−3
−2
−1
0
1
2
Concentration (log10) [µ M]
Rapamycin
+C
+P

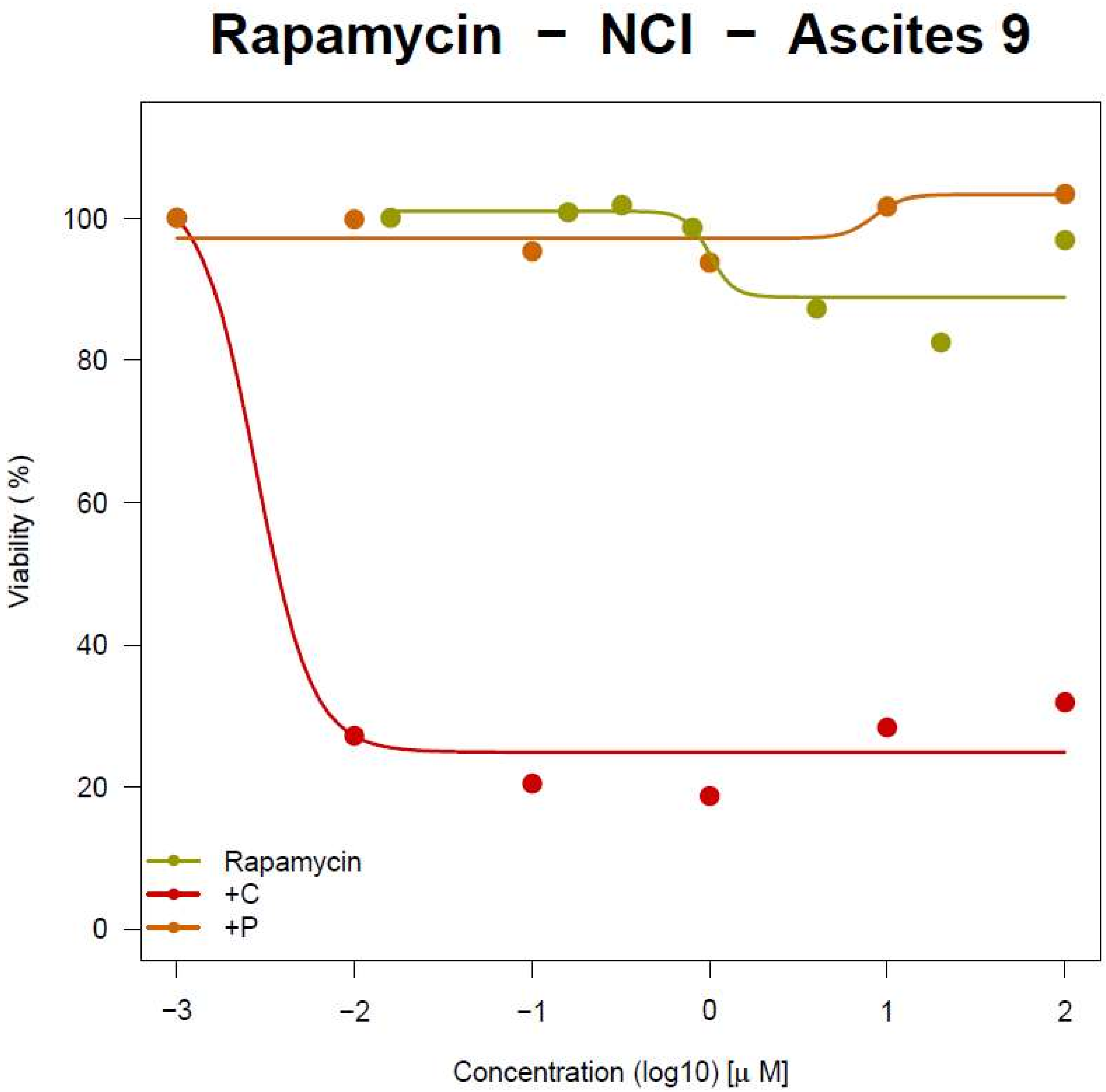

Rapamycin − NCI − Ascites 9
Viability (%)
Concentration (log10) [μ M]
Rapamycin
+C
+P
0
20
40
60
80
100
−3
−2
−1
0
1
2

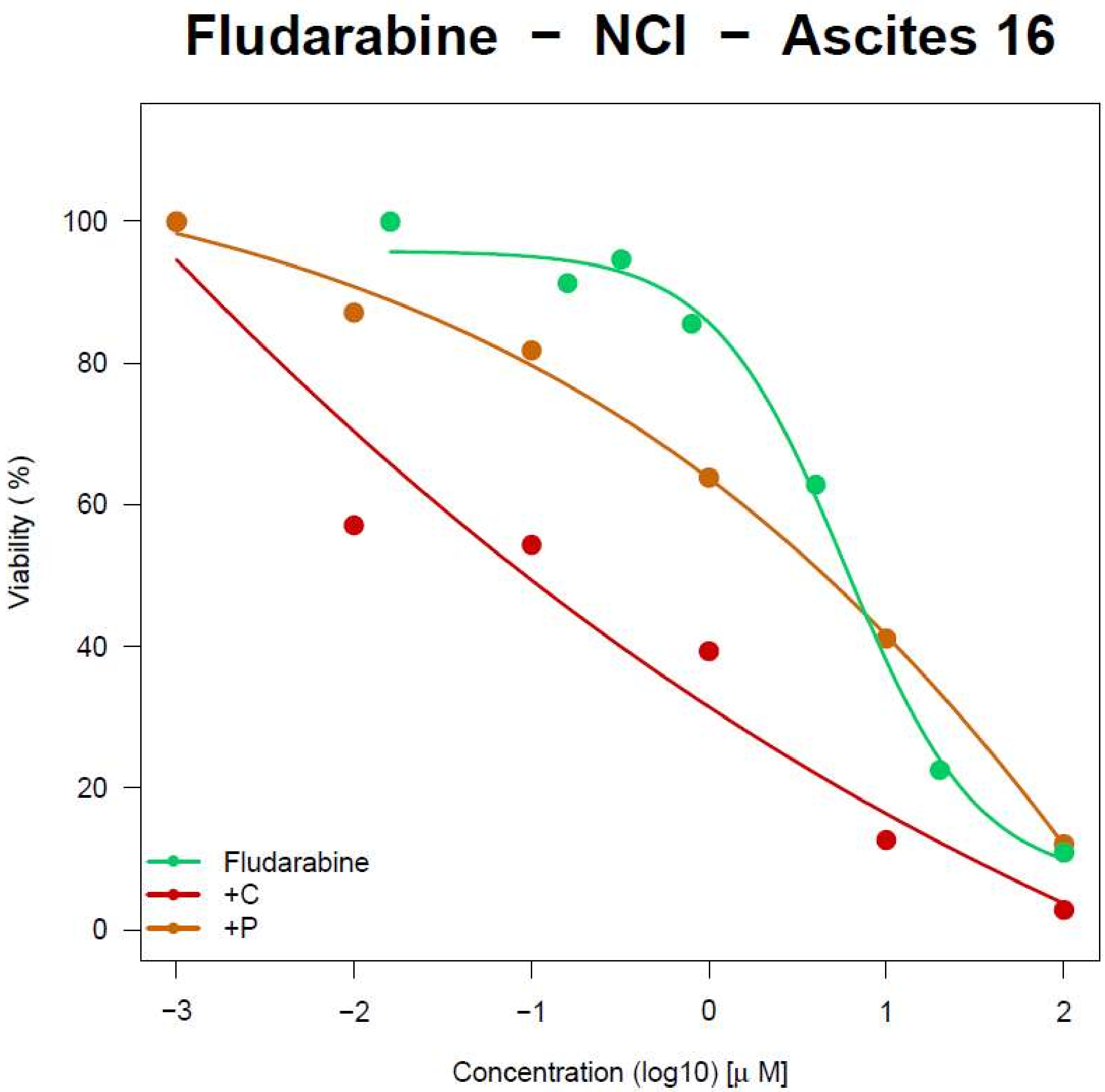

Fludarabine − NCI − Ascites 16
Viability (%)
100
80
60
40
20
0
−3
−2
−1
0
1
2
Concentration (log10) [μ M]
Fludarabine
+C
+P

Fludarabine – NCI – Ascites 18

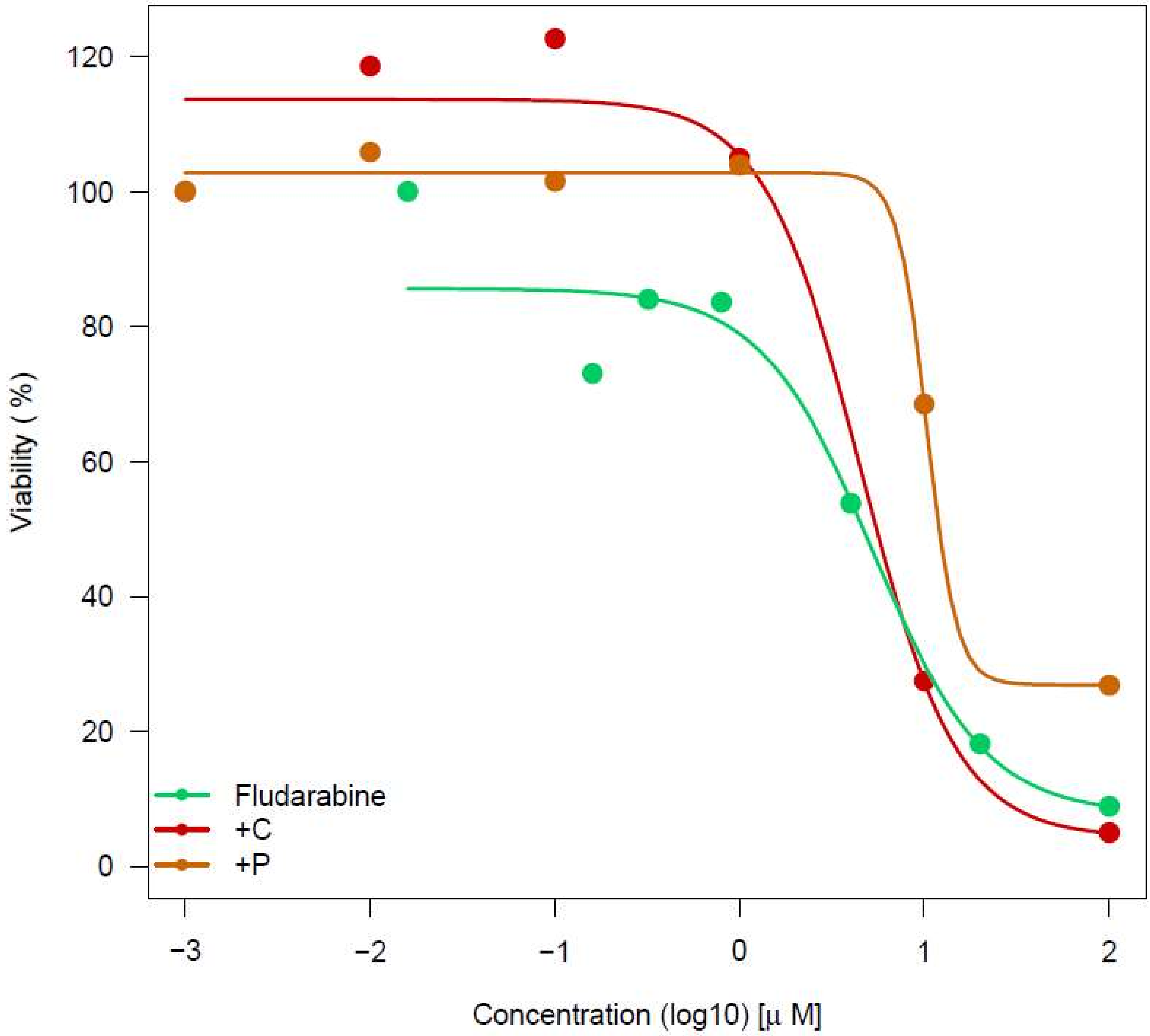

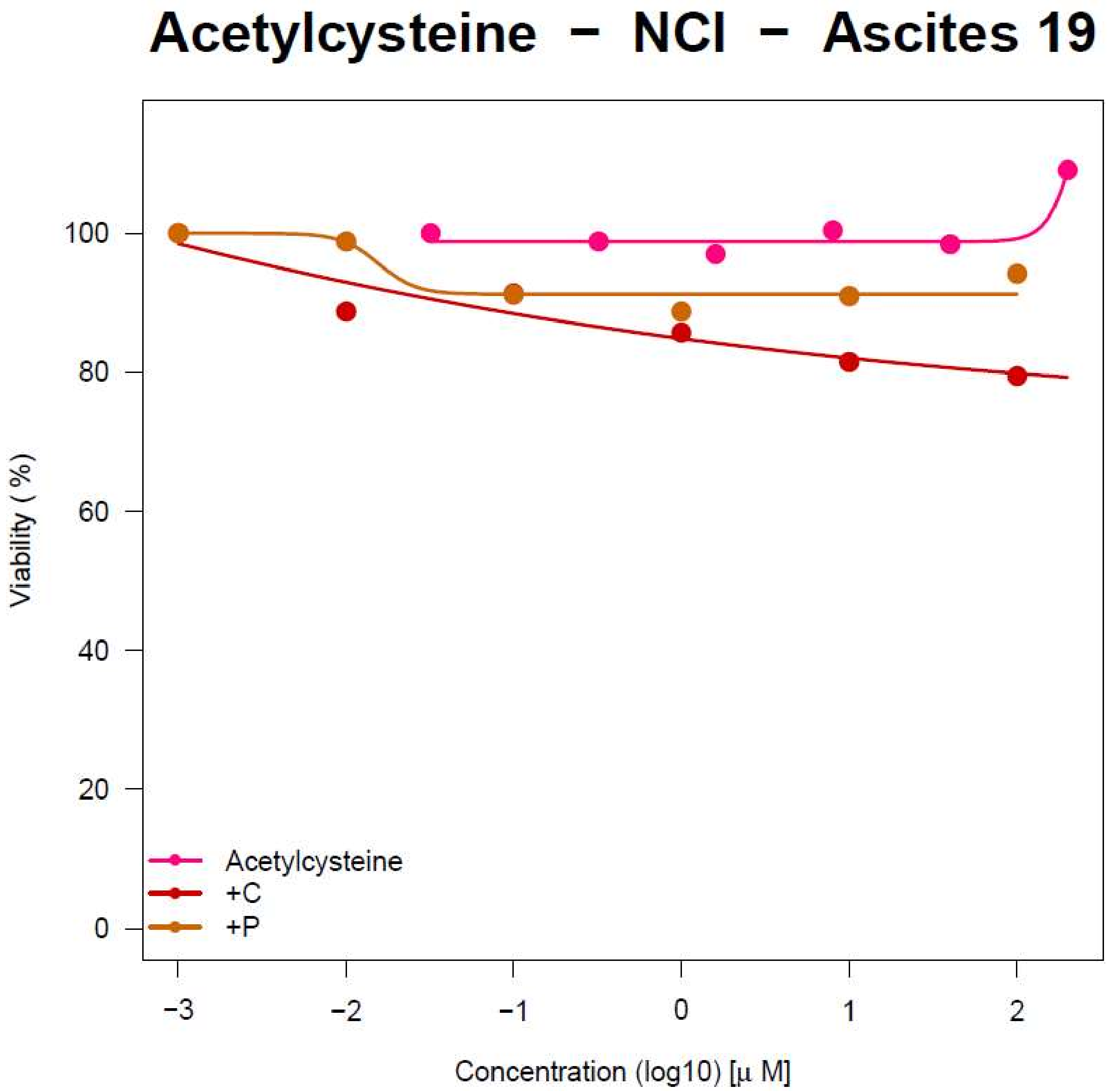

Acetylcysteine − NCI − Ascites 19
Viability (%)
100
80
60
40
20
0
Acetylcysteine
+C
+P
−3
−2
−1
0
1
2
Concentration (log10) [µ M]

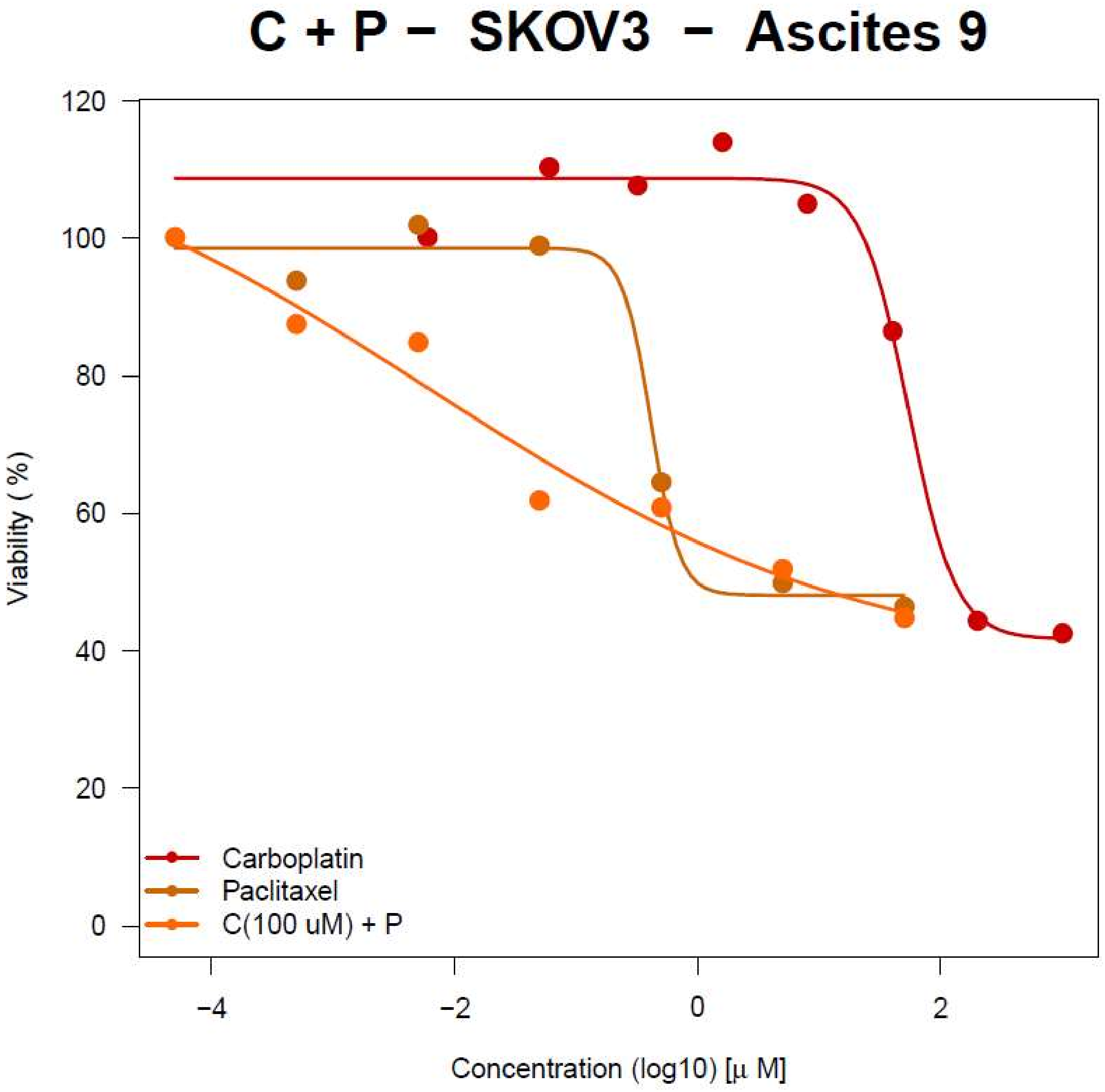
C + P – SKOV3 – Ascites 9
Viability (%)
Concentration (log10) [μ M]
Carboplatin
Paclitaxel
C(100 uM) + P
120
100
80
60
40
20
0
−4
−2
0
2

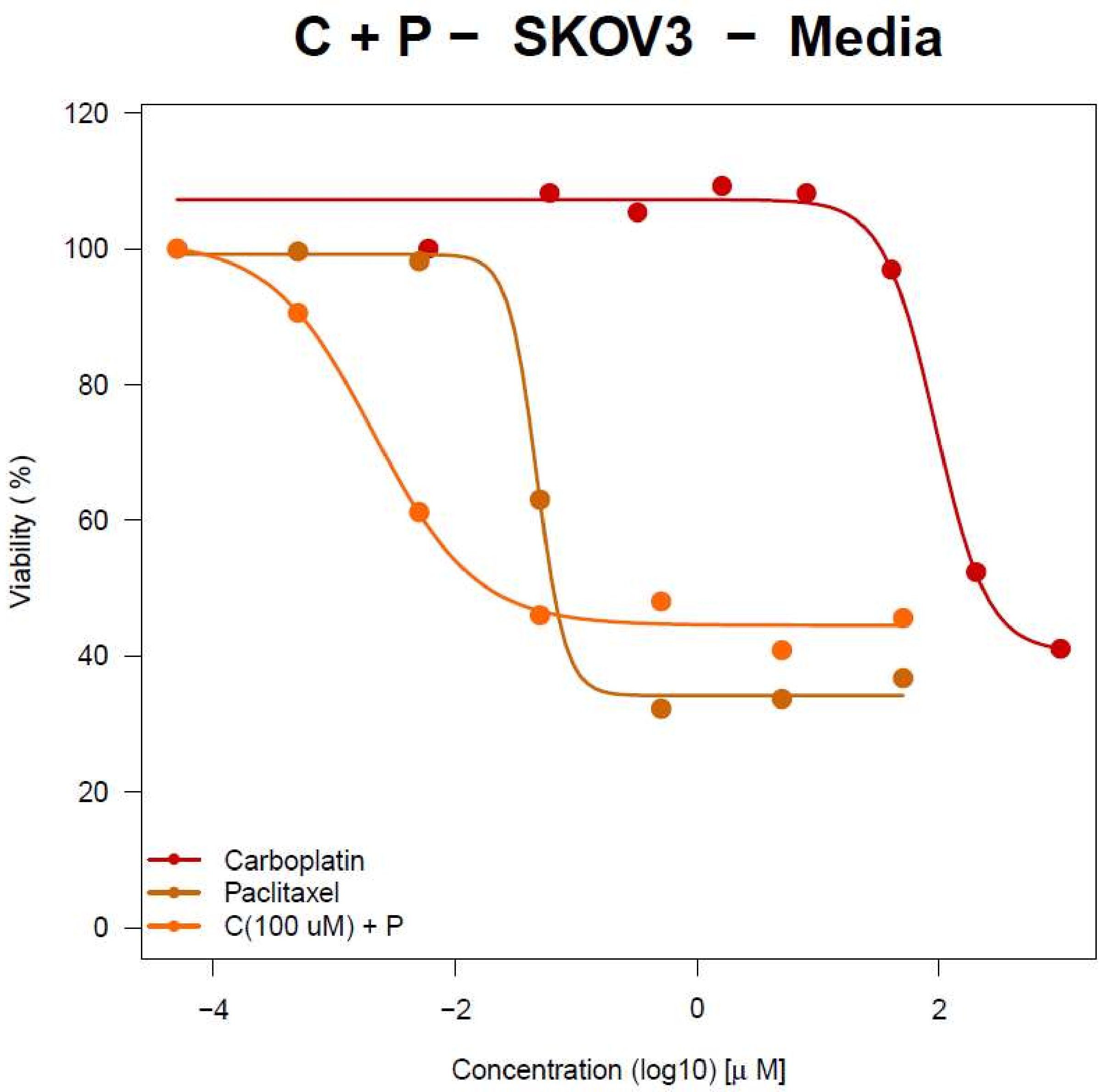

C + P − SKOV3 − Media
Viability (%)
120
100
80
60
40
20
0
−4
−2
0
2
Concentration (log10) [μ M]
Carboplatin
Paclitaxel
C(100 uM) + P

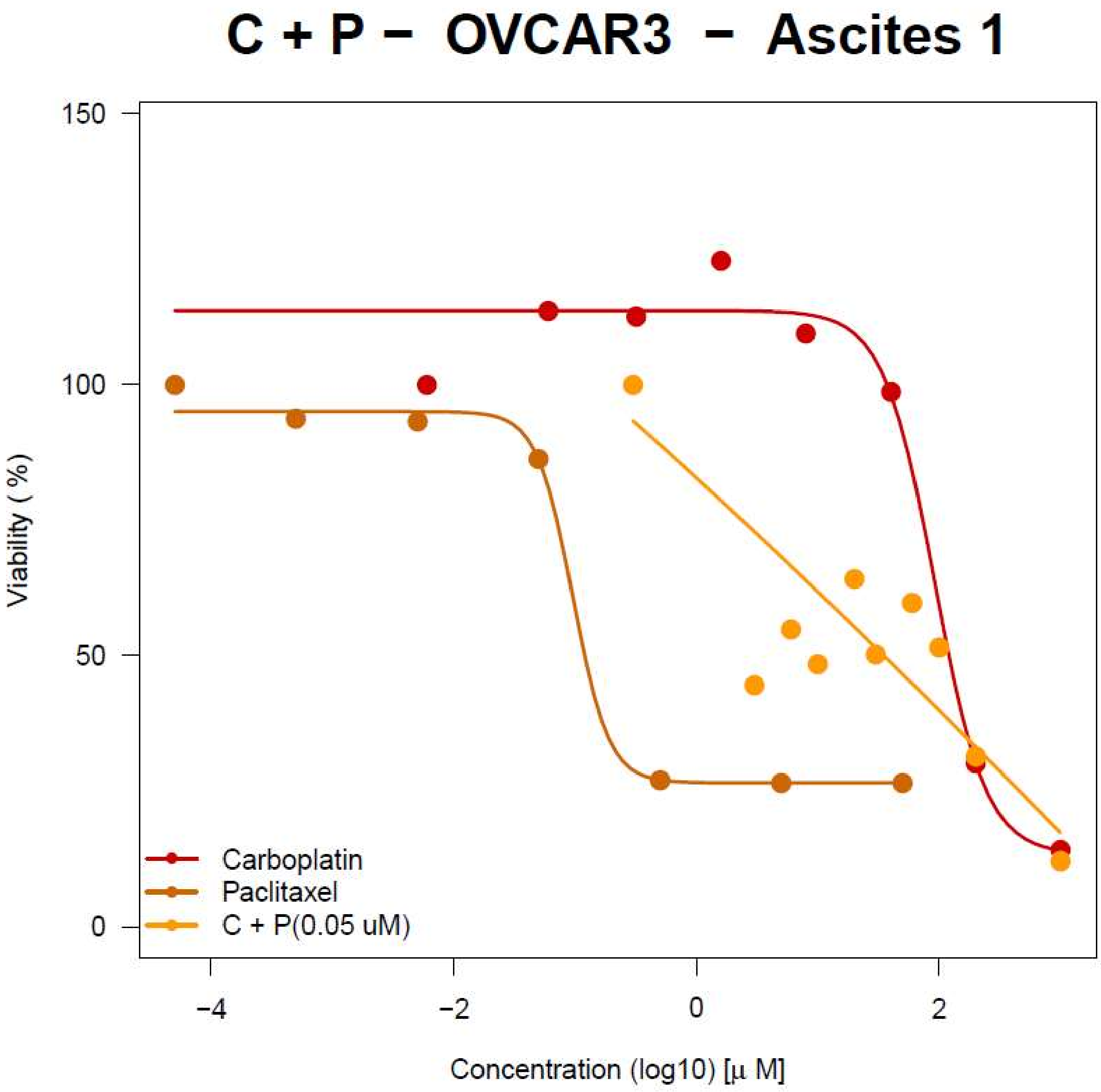
C + P – OVCAR3 – Ascites 1
150
100
50
0
Viability (%)
−4
−2
0
2
Concentration (log10) [μ M]
Carboplatin
Paclitaxel
C + P(0.05 uM)

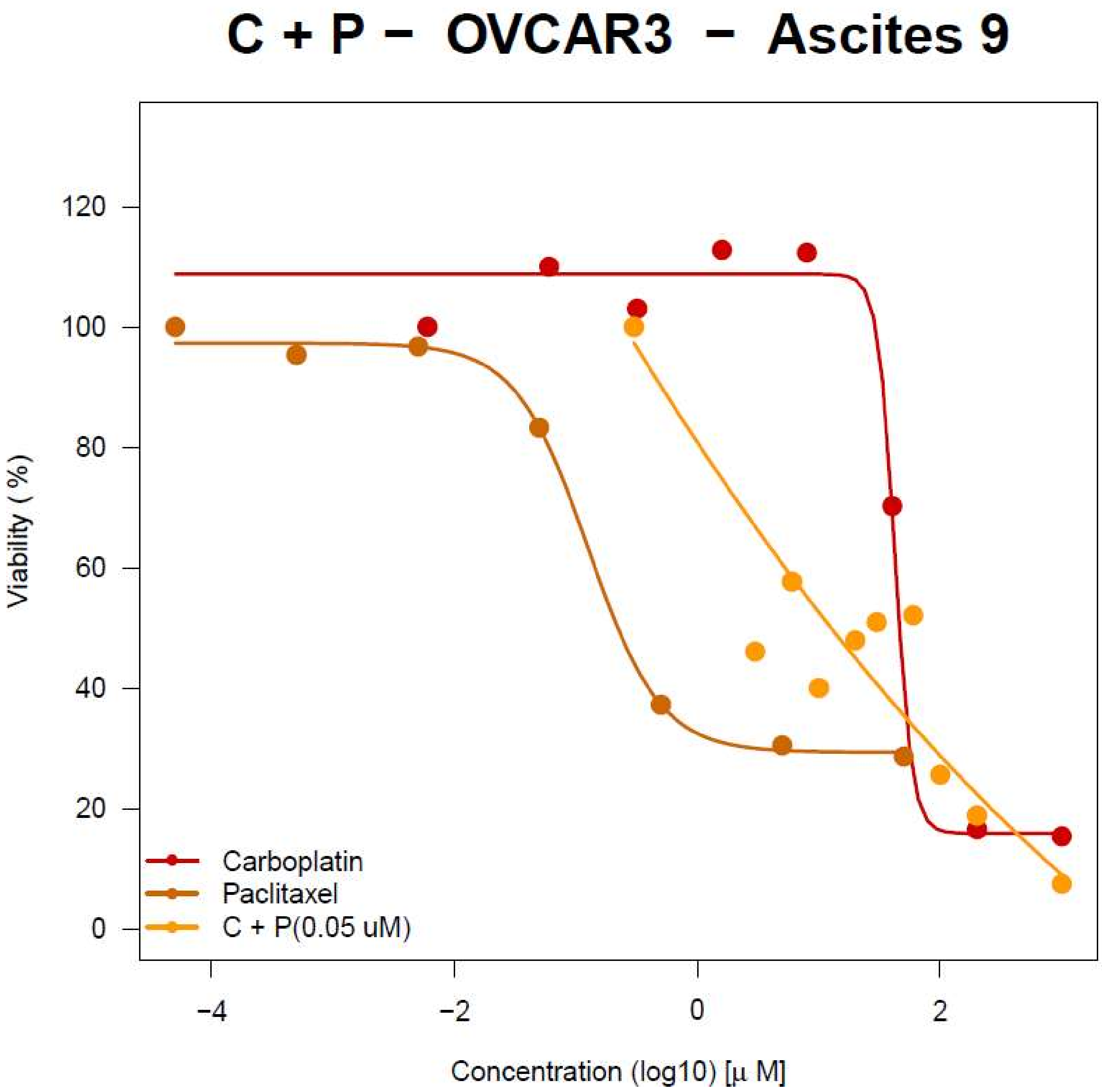

C + P − OVCAR3 − Ascites 9
120
100
80
60
40
20
0
Viability (%)
Carboplatin
Paclitaxel
C + P(0.05 uM)
−4
−2
0
2
Concentration (log10) [μ M]

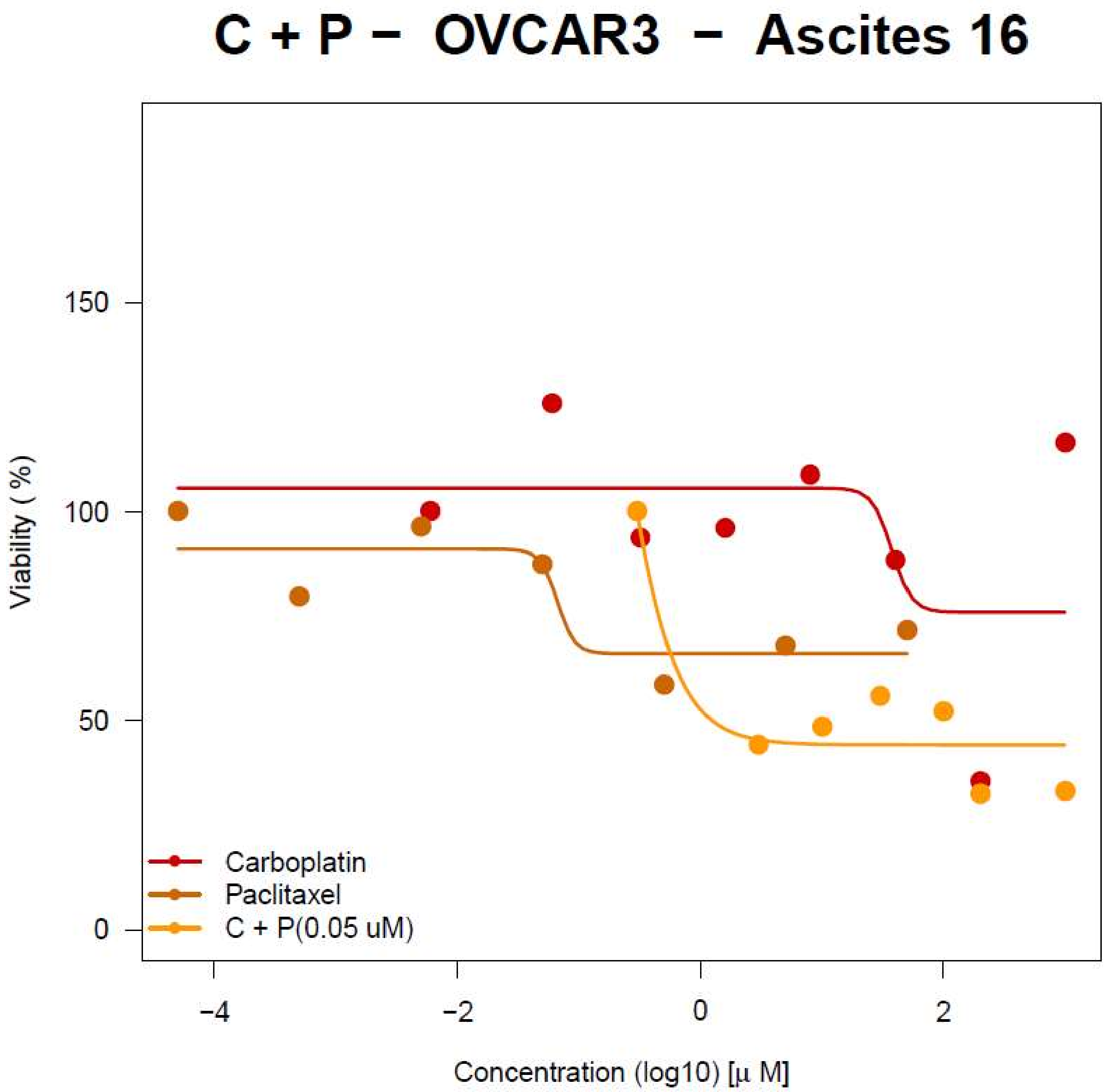

C + P − OVCAR3 − Ascites 16
Viability (%)
0
50
100
150
−4
−2
0
2
Concentration (log10) [µ M]
Carboplatin
Paclitaxel
C + P(0.05 uM)

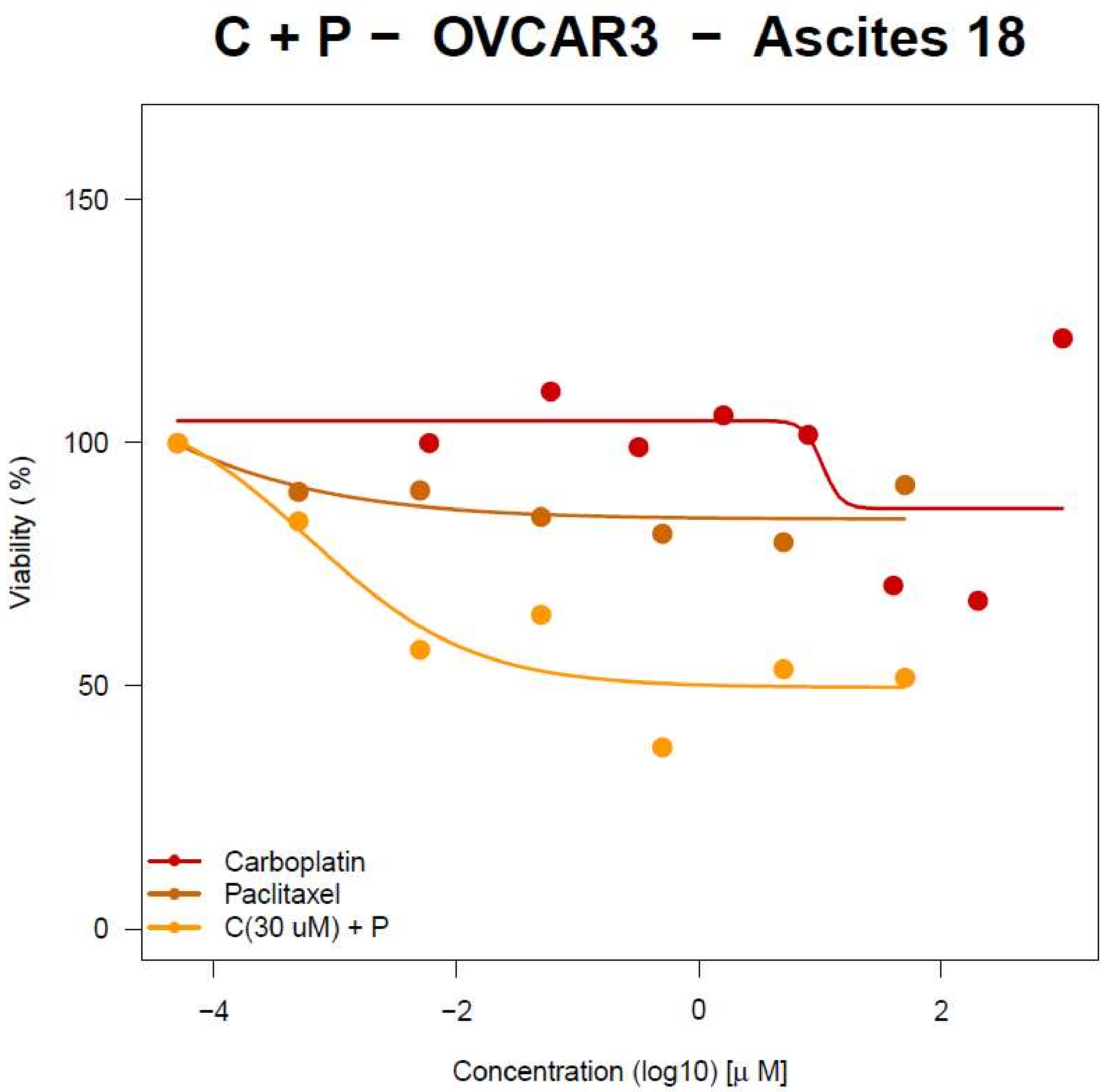
C + P – OVCAR3 – Ascites 18
150
100
50
0
Viability (%)
−4
−2
0
2
Concentration (log10) [μ M]
Carboplatin
Paclitaxel
C(30 uM) + P

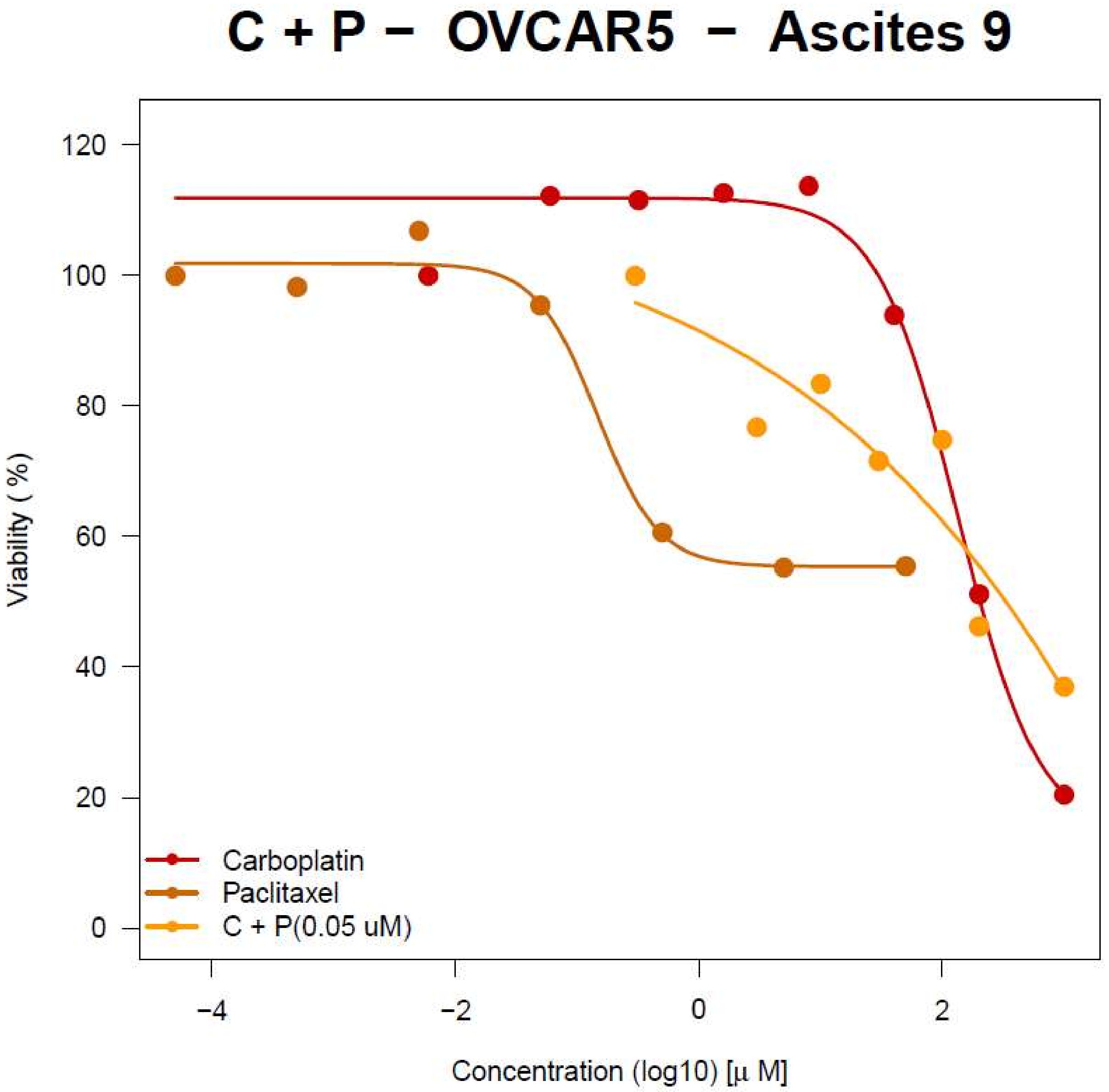
C + P − OVCAR5 − Ascites 9
Viability (%)
Concentration (log10) [μ M]
Carboplatin
Paclitaxel
C + P(0.05 uM)

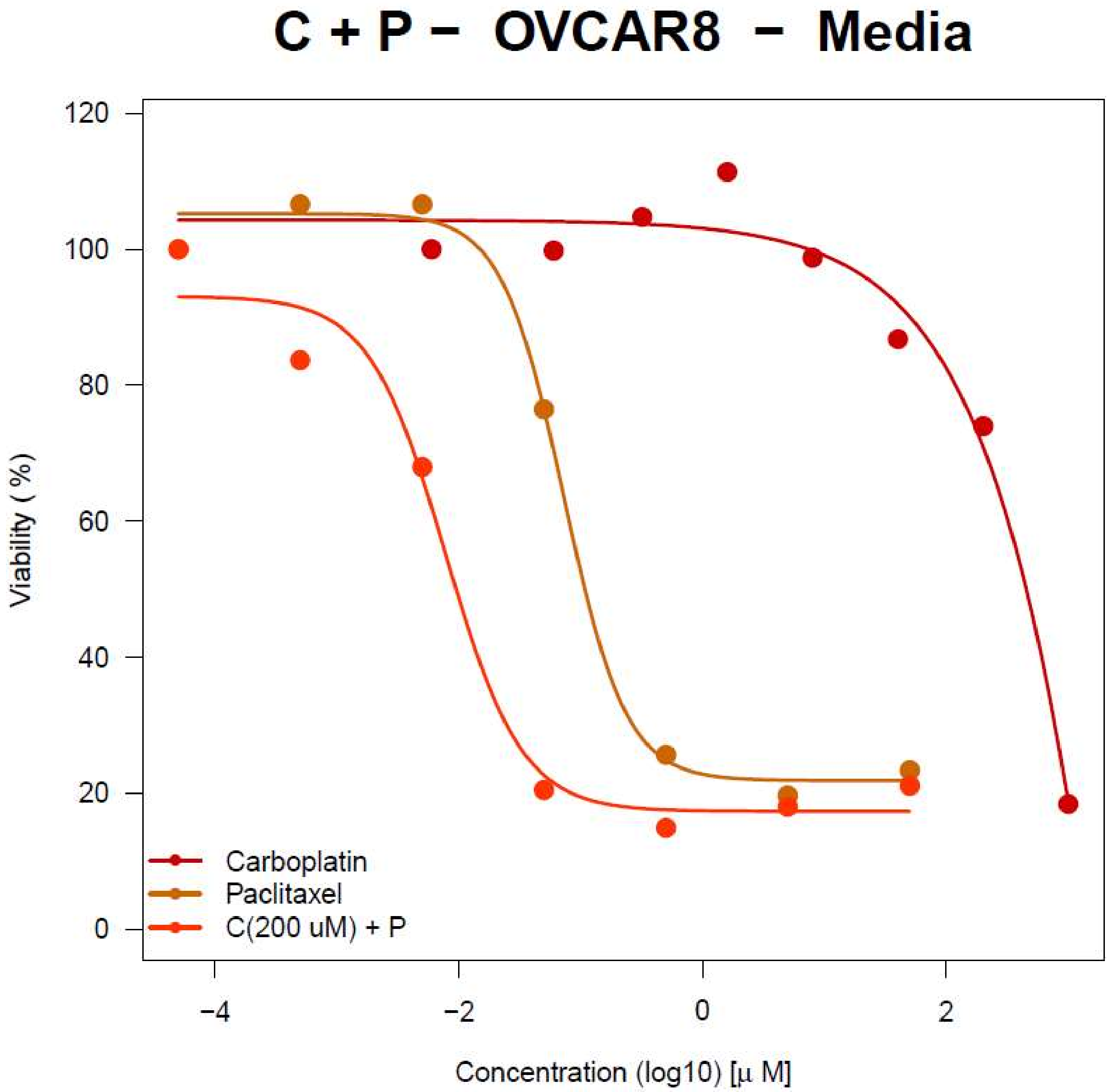
C + P − OVCAR8 − Media
Viability (%)
Concentration (log10) [μ M]
Carboplatin
Paclitaxel
C(200 uM) + P
120
100
80
60
40
20
0
−4
−2
0
2

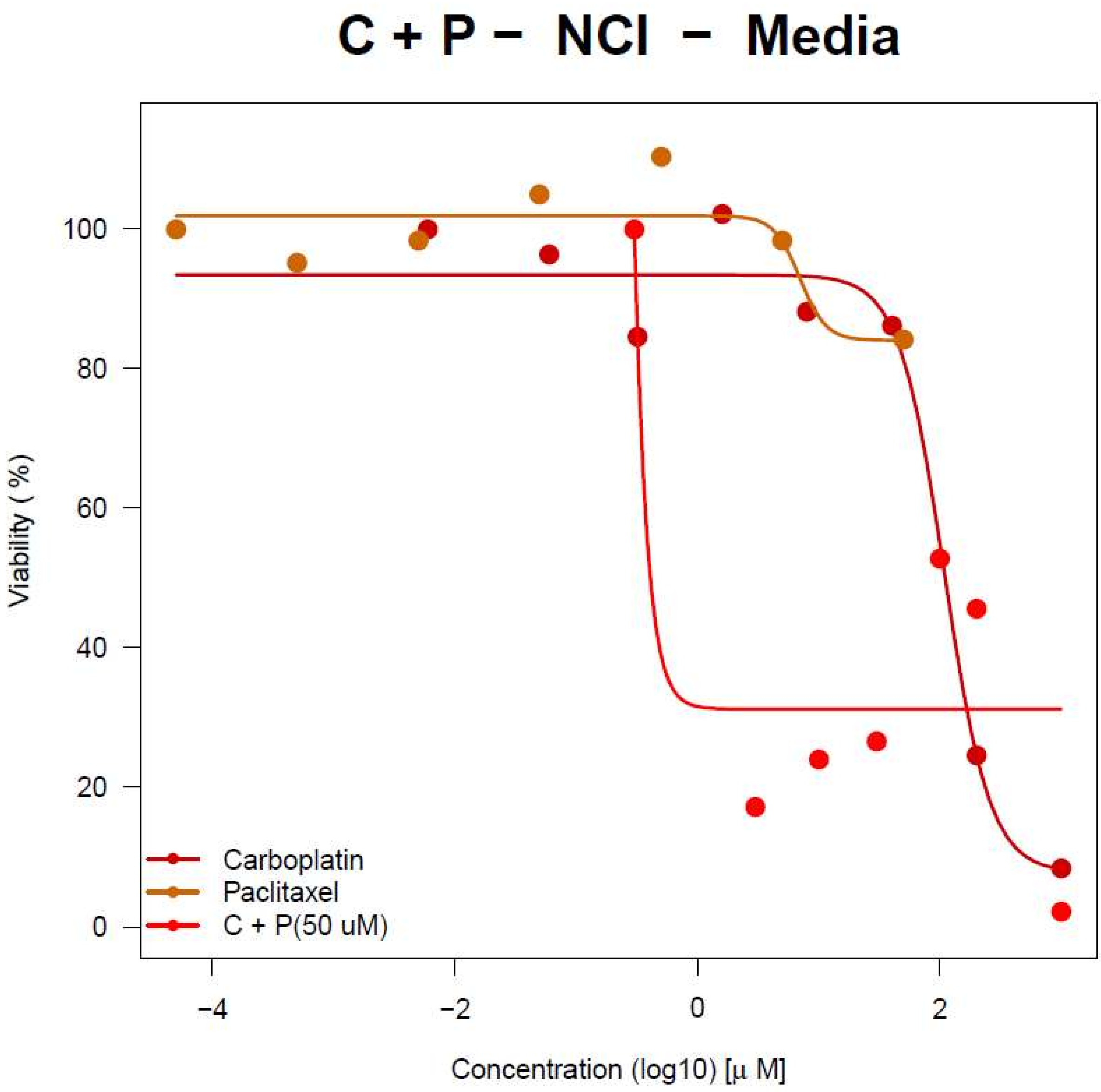

Supplementary Figure 9. Additional data from Table 4 are presented as graphs indicating response to treatment in ascending doses (log10 concentrations on the X axes) to novel therapeutics alone or together with standard-of-care drugs carboplatin (C) or paclitaxel (P) in cell lines after incubation with patient-derived ascites or media alone. In cases when the combination of paclitaxel and carboplatin was found to be the most effective, only the standard of care (single agent or in combination) were shown. When combing paclitaxel and carboplatin, one of the drugs was used at a defined concentration (IC20) for consistency.

Supplementary Figure 10

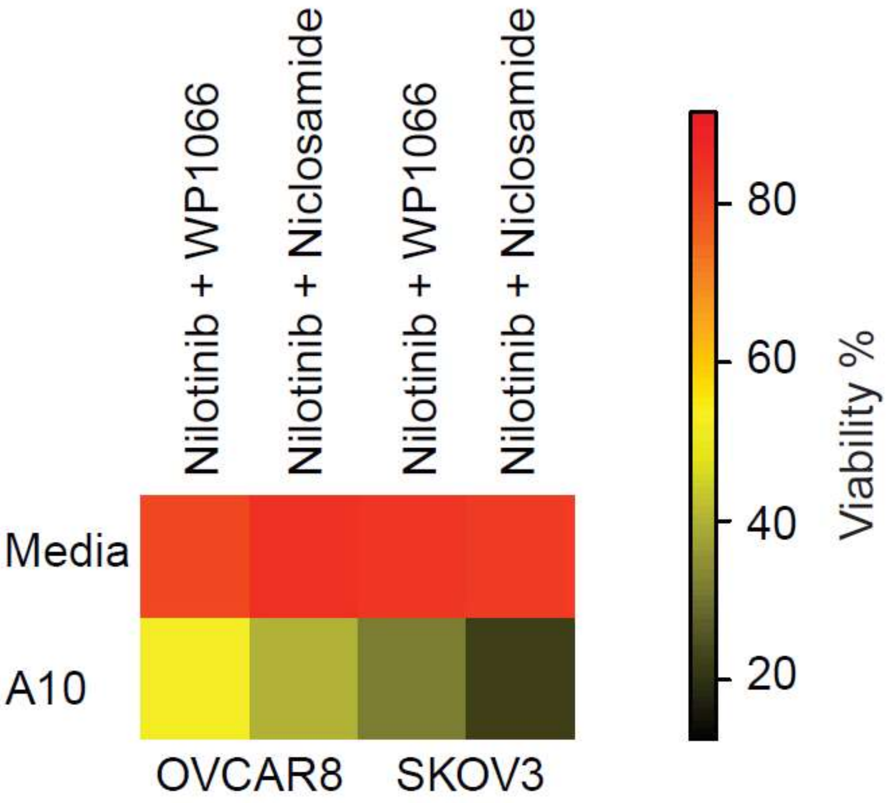

Supplementary Figure 10. Combination treatment with novel therapeutics.
Heatmap illustrating values of 100-VUS (volume under the surface) for combination treatment with STAT3 inhibitors Nilotinib and Niclosamide vs. treatment combining Nilotinib and the JAK inhibitor WP1066 in OVCAR8 and SKOV3 cells incubated with patient-derived ascites (A10) or media.
